# Supplementary material for: Brain tropism acquisition: The spatial dynamics and evolution of a measles virus collective infectious unit that drove lethal subacute sclerosing panencephalitis
Source: PLoS Pathog. 2023 Dec 21;19(12):e1011817. doi: 10.1371/journal.ppat.1011817 (PMC10735034; doi:10.1371/journal.ppat.1011817)
Supplement: S1 Table — (DOCX) [file ppat.1011817.s001.docx]

**Extended data Table 1. Total and MeV reads from deep sequencing.**

| **Sample** | **Reads (Millions)** | |
| --- | --- | --- |
|  | **Total** | **MeV** |
| SSPE1 | 7.4 | 1.3 |
| SSPE2 | 8.7 | 0.7 |
| Frontal Cortex 1 | 69.6 | 13.4 |
| Frontal Cortex 2 | 94.7 | 18.3 |
| Frontal Cortex 3 | 88.6 | 11.1 |
| Parietal lobe | 74.6 | 15.1 |
| Temporal lobe | 102 | 11.9 |
| Occipital lobe | 61.8 | 7.4 |
| Hippocampus | 71.6 | 6.7 |
| Internal capsule | 70.8 | 1.8 |
| Midbrain | 73.6 | 3 |
| Upper brainstem | 67.5 | 3.2 |
| Brainstem | 54.7 | 1.2 |
| Cerebellum | 98.1 | 0.1 |
| Cerebellum towards nucleus | 80.9 | 0.4 |
