# Supplementary material for: Brain tropism acquisition: The spatial dynamics and evolution of a measles virus collective infectious unit that drove lethal subacute sclerosing panencephalitis
Source: PLoS Pathog. 2023 Dec 21;19(12):e1011817. doi: 10.1371/journal.ppat.1011817 (PMC10735034; doi:10.1371/journal.ppat.1011817)
Supplement: S2 Table — (DOCX) [file ppat.1011817.s002.docx]

**Extended data Table 2. Frequency of SNVs in different brain regions**

| Tissue | Allele Frequency | Background | Haplotype | Reference | Position | Variant |
| --- | --- | --- | --- | --- | --- | --- |
| Temporal Lobe | 0.9056 | Genome-2 | Genome-2 | T | 537 | C |
| Parietal Lobe | 0.3525 | Genome-2 | Genome-2 | T | 537 | C |
| Upper Brain Stem | 0.1853 | Genome-2 | Genome-2 | T | 537 | C |
| Midbrain | 0.2455 | Genome-2 | Genome-2 | T | 537 | C |
| Frontal Cortex 1 | 0.3664 | Genome-2 | Genome-2 | T | 537 | C |
| SSPE 2 | 0.2795 | Genome-2 | Genome-2 | T | 537 | C |
| Brain Stem | 0.0985 | Genome-2 | Genome-2 | T | 537 | C |
| Occipital Lobe | 0.8739 | Genome-2 | Genome-2 | T | 537 | C |
| SSPE 1 | 0.3265 | Genome-2 | Genome-2 | T | 537 | C |
| Cerebellum | 0.1307 | Genome-2 | Genome-2 | T | 537 | C |
| Cerebellum Nucleus | 0.3061 | Genome-2 | Genome-2 | T | 537 | C |
| Frontal Cortex 2 | 0.4146 | Genome-2 | Genome-2 | T | 537 | C |
| Internal Capsule | 0.5717 | Genome-2 | Genome-2 | T | 537 | C |
| Frontal Cortex 3 | 0.3685 | Genome-2 | Genome-2 | T | 537 | C |
| Hippocampus | 0.7672 | Genome-2 | Genome-2 | T | 537 | C |
| Temporal Lobe | 0.035 | Genome-1 | cluster 5 | C | 811 | G |
| Parietal Lobe | 0.15 | Genome-1 | cluster 5 | C | 811 | G |
| Upper Brain Stem | 0.0641 | Genome-1 | cluster 5 | C | 811 | G |
| Midbrain | 0 | Genome-1 | cluster 5 | C | 811 | G |
| Frontal Cortex 1 | 0 | Genome-1 | cluster 5 | C | 811 | G |
| SSPE 2 | 0 | Genome-1 | cluster 5 | C | 811 | G |
| Brain Stem | 0.135 | Genome-1 | cluster 5 | C | 811 | G |
| Occipital Lobe | 0.0471 | Genome-1 | cluster 5 | C | 811 | G |
| SSPE 1 | 0 | Genome-1 | cluster 5 | C | 811 | G |
| Cerebellum | 0.0327 | Genome-1 | cluster 5 | C | 811 | G |
| Cerebellum Nucleus | 0.0311 | Genome-1 | cluster 5 | C | 811 | G |
| Frontal Cortex 2 | 0 | Genome-1 | cluster 5 | C | 811 | G |
| Internal Capsule | 0.1897 | Genome-1 | cluster 5 | C | 811 | G |
| Frontal Cortex 3 | 0 | Genome-1 | cluster 5 | C | 811 | G |
| Hippocampus | 0.0729 | Genome-1 | cluster 5 | C | 811 | G |
| Temporal Lobe | 0.0957 | Genome-1 | Genome-1 | T | 1328 | C |
| Parietal Lobe | 0.6289 | Genome-1 | Genome-1 | T | 1328 | C |
| Upper Brain Stem | 0.7992 | Genome-1 | Genome-1 | T | 1328 | C |
| Midbrain | 0.7274 | Genome-1 | Genome-1 | T | 1328 | C |
| Frontal Cortex 1 | 0.6427 | Genome-1 | Genome-1 | T | 1328 | C |
| SSPE 2 | 0.7119 | Genome-1 | Genome-1 | T | 1328 | C |
| Brain Stem | 0.9031 | Genome-1 | Genome-1 | T | 1328 | C |
| Occipital Lobe | 0.0763 | Genome-1 | Genome-1 | T | 1328 | C |
| SSPE 1 | 0.6809 | Genome-1 | Genome-1 | T | 1328 | C |
| Cerebellum | 0.8404 | Genome-1 | Genome-1 | T | 1328 | C |
| Cerebellum Nucleus | 0.6892 | Genome-1 | Genome-1 | T | 1328 | C |
| Frontal Cortex 2 | 0.0342 | Genome-1 | Genome-1 | T | 1328 | C |
| Internal Capsule | 0.4346 | Genome-1 | Genome-1 | T | 1328 | C |
| Frontal Cortex 3 | 0.61 | Genome-1 | Genome-1 | T | 1328 | C |
| Hippocampus | 0.2222 | Genome-1 | Genome-1 | T | 1328 | C |
| Temporal Lobe | 0.0207 | Genome-1 | cluster 2 | T | 1620 | C |
| Parietal Lobe | 0.0204 | Genome-1 | cluster 2 | T | 1620 | C |
| Upper Brain Stem | 0.1097 | Genome-1 | cluster 2 | T | 1620 | C |
| Midbrain | 0.322 | Genome-1 | cluster 2 | T | 1620 | C |
| Frontal Cortex 1 | 0 | Genome-1 | cluster 2 | T | 1620 | C |
| SSPE 2 | 0 | Genome-1 | cluster 2 | T | 1620 | C |
| Brain Stem | 0.4533 | Genome-1 | cluster 2 | T | 1620 | C |
| Occipital Lobe | 0.028 | Genome-1 | cluster 2 | T | 1620 | C |
| SSPE 1 | 0 | Genome-1 | cluster 2 | T | 1620 | C |
| Cerebellum | 0.4412 | Genome-1 | cluster 2 | T | 1620 | C |
| Cerebellum Nucleus | 0.3371 | Genome-1 | cluster 2 | T | 1620 | C |
| Frontal Cortex 2 | 0.0556 | Genome-1 | cluster 2 | T | 1620 | C |
| Internal Capsule | 0 | Genome-1 | cluster 2 | T | 1620 | C |
| Frontal Cortex 3 | 0 | Genome-1 | cluster 2 | T | 1620 | C |
| Hippocampus | 0 | Genome-1 | cluster 2 | T | 1620 | C |
| Temporal Lobe | 0.2536 | Genome-2 | cluster 7 | G | 1622 | A |
| Parietal Lobe | 0.1073 | Genome-2 | cluster 7 | G | 1622 | A |
| Upper Brain Stem | 0 | Genome-2 | cluster 7 | G | 1622 | A |
| Midbrain | 0 | Genome-2 | cluster 7 | G | 1622 | A |
| Frontal Cortex 1 | 0 | Genome-2 | cluster 7 | G | 1622 | A |
| SSPE 2 | 0 | Genome-2 | cluster 7 | G | 1622 | A |
| Brain Stem | 0 | Genome-2 | cluster 7 | G | 1622 | A |
| Occipital Lobe | 0.5432 | Genome-2 | cluster 7 | G | 1622 | A |
| SSPE 1 | 0 | Genome-2 | cluster 7 | G | 1622 | A |
| Cerebellum | 0.02019 | Genome-2 | cluster 7 | G | 1622 | A |
| Cerebellum Nucleus | 0.0456 | Genome-2 | cluster 7 | G | 1622 | A |
| Frontal Cortex 2 | 0.3831 | Genome-2 | cluster 7 | G | 1622 | A |
| Internal Capsule | 0.2289 | Genome-2 | cluster 7 | G | 1622 | A |
| Frontal Cortex 3 | 0 | Genome-2 | cluster 7 | G | 1622 | A |
| Hippocampus | 0.4367 | Genome-2 | cluster 7 | G | 1622 | A |
| Temporal Lobe | 0.0821 | Genome-1 | Genome-01 | T | 2139 | C |
| Parietal Lobe | 0.6231 | Genome-1 | Genome-01 | T | 2139 | C |
| Upper Brain Stem | 0.8059 | Genome-1 | Genome-01 | T | 2139 | C |
| Midbrain | 0.7466 | Genome-1 | Genome-01 | T | 2139 | C |
| Frontal Cortex 1 | 0.6261 | Genome-1 | Genome-01 | T | 2139 | C |
| SSPE 2 | 0.7135 | Genome-1 | Genome-01 | T | 2139 | C |
| Brain Stem | 0.9003 | Genome-1 | Genome-01 | T | 2139 | C |
| Occipital Lobe | 0.0829 | Genome-1 | Genome-01 | T | 2139 | C |
| SSPE 1 | 0.6473 | Genome-1 | Genome-01 | T | 2139 | C |
| Cerebellum | 0.8753 | Genome-1 | Genome-01 | T | 2139 | C |
| Cerebellum Nucleus | 0.7218 | Genome-1 | Genome-01 | T | 2139 | C |
| Frontal Cortex 2 | 0.5603 | Genome-1 | Genome-01 | T | 2139 | C |
| Internal Capsule | 0.4034 | Genome-1 | Genome-01 | T | 2139 | C |
| Frontal Cortex 3 | 0.6312 | Genome-1 | Genome-01 | T | 2139 | C |
| Hippocampus | 0.2252 | Genome-1 | Genome-01 | T | 2139 | C |
| Temporal Lobe | 0.9055 | Genome-2 | Genome-2 | C | 2406 | A |
| Parietal Lobe | 0.3764 | Genome-2 | Genome-2 | C | 2406 | A |
| Upper Brain Stem | 0.1698 | Genome-2 | Genome-2 | C | 2406 | A |
| Midbrain | 0.2257 | Genome-2 | Genome-2 | C | 2406 | A |
| Frontal Cortex 1 | 0.3441 | Genome-2 | Genome-2 | C | 2406 | A |
| SSPE 2 | 0.2798 | Genome-2 | Genome-2 | C | 2406 | A |
| Brain Stem | 0.0821 | Genome-2 | Genome-2 | C | 2406 | A |
| Occipital Lobe | 0.8747 | Genome-2 | Genome-2 | C | 2406 | A |
| SSPE 1 | 0.3352 | Genome-2 | Genome-2 | C | 2406 | A |
| Cerebellum | 0.1089 | Genome-2 | Genome-2 | C | 2406 | A |
| Cerebellum Nucleus | 0.2448 | Genome-2 | Genome-2 | C | 2406 | A |
| Frontal Cortex 2 | 0.4475 | Genome-2 | Genome-2 | C | 2406 | A |
| Internal Capsule | 0.5711 | Genome-2 | Genome-2 | C | 2406 | A |
| Frontal Cortex 3 | 0.3529 | Genome-2 | Genome-2 | C | 2406 | A |
| Hippocampus | 0.7755 | Genome-2 | Genome-2 | C | 2406 | A |
| Temporal Lobe | 0.0853 | Genome-1 | Genome-01 | A | 2429 | G |
| Parietal Lobe | 0.6092 | Genome-1 | Genome-01 | A | 2429 | G |
| Upper Brain Stem | 0.8188 | Genome-1 | Genome-01 | A | 2429 | G |
| Midbrain | 0.7646 | Genome-1 | Genome-01 | A | 2429 | G |
| Frontal Cortex 1 | 0.6334 | Genome-1 | Genome-01 | A | 2429 | G |
| SSPE 2 | 0.701 | Genome-1 | Genome-01 | A | 2429 | G |
| Brain Stem | 0.9082 | Genome-1 | Genome-01 | A | 2429 | G |
| Occipital Lobe | 0.0837 | Genome-1 | Genome-01 | A | 2429 | G |
| SSPE 1 | 0.6407 | Genome-1 | Genome-01 | A | 2429 | G |
| Cerebellum | 0.8805 | Genome-1 | Genome-01 | A | 2429 | G |
| Cerebellum Nucleus | 0.7337 | Genome-1 | Genome-01 | A | 2429 | G |
| Frontal Cortex 2 | 0.5198 | Genome-1 | Genome-01 | A | 2429 | G |
| Internal Capsule | 0.4022 | Genome-1 | Genome-01 | A | 2429 | G |
| Frontal Cortex 3 | 0.6275 | Genome-1 | Genome-01 | A | 2429 | G |
| Hippocampus | 0.2079 | Genome-1 | Genome-01 | A | 2429 | G |
| Temporal Lobe | 0.9192 | Genome-2 | Genome-2 | T | 2725 | C |
| Parietal Lobe | 0.3838 | Genome-2 | Genome-2 | T | 2725 | C |
| Upper Brain Stem | 0.1991 | Genome-2 | Genome-2 | T | 2725 | C |
| Midbrain | 0.2616 | Genome-2 | Genome-2 | T | 2725 | C |
| Frontal Cortex 1 | 0.3836 | Genome-2 | Genome-2 | T | 2725 | C |
| SSPE 2 | 0.2851 | Genome-2 | Genome-2 | T | 2725 | C |
| Brain Stem | 0.1003 | Genome-2 | Genome-2 | T | 2725 | C |
| Occipital Lobe | 0.8917 | Genome-2 | Genome-2 | T | 2725 | C |
| SSPE 1 | 0.3707 | Genome-2 | Genome-2 | T | 2725 | C |
| Cerebellum | 0.1384 | Genome-2 | Genome-2 | T | 2725 | C |
| Cerebellum Nucleus | 0.3154 | Genome-2 | Genome-2 | T | 2725 | C |
| Frontal Cortex 2 | 0.4478 | Genome-2 | Genome-2 | T | 2725 | C |
| Internal Capsule | 0.6012 | Genome-2 | Genome-2 | T | 2725 | C |
| Frontal Cortex 3 | 0.3849 | Genome-2 | Genome-2 | T | 2725 | C |
| Hippocampus | 0.7905 | Genome-2 | Genome-2 | T | 2725 | C |
| Temporal Lobe | 0.9108 | Genome-2 | Genome-2 | A | 3139 | G |
| Parietal Lobe | 0.327 | Genome-2 | Genome-2 | A | 3139 | G |
| Upper Brain Stem | 0.1684 | Genome-2 | Genome-2 | A | 3139 | G |
| Midbrain | 0.2404 | Genome-2 | Genome-2 | A | 3139 | G |
| Frontal Cortex 1 | 0.3426 | Genome-2 | Genome-2 | A | 3139 | G |
| SSPE 2 | 0.2708 | Genome-2 | Genome-2 | A | 3139 | G |
| Brain Stem | 0.0881 | Genome-2 | Genome-2 | A | 3139 | G |
| Occipital Lobe | 0.8821 | Genome-2 | Genome-2 | A | 3139 | G |
| SSPE 1 | 0.3499 | Genome-2 | Genome-2 | A | 3139 | G |
| Cerebellum | 0.0794 | Genome-2 | Genome-2 | A | 3139 | G |
| Cerebellum Nucleus | 0.257 | Genome-2 | Genome-2 | A | 3139 | G |
| Frontal Cortex 2 | 0.3812 | Genome-2 | Genome-2 | A | 3139 | G |
| Internal Capsule | 0.556 | Genome-2 | Genome-2 | A | 3139 | G |
| Frontal Cortex 3 | 0.3174 | Genome-2 | Genome-2 | A | 3139 | G |
| Hippocampus | 0.754 | Genome-2 | Genome-2 | A | 3139 | G |
| Temporal Lobe | 0.9098 | Genome-2 | Genome-2 | A | 3140 | G |
| Parietal Lobe | 0.3257 | Genome-2 | Genome-2 | A | 3140 | G |
| Upper Brain Stem | 0.1678 | Genome-2 | Genome-2 | A | 3140 | G |
| Midbrain | 0.2386 | Genome-2 | Genome-2 | A | 3140 | G |
| Frontal Cortex 1 | 0.3426 | Genome-2 | Genome-2 | A | 3140 | G |
| SSPE 2 | 0.2701 | Genome-2 | Genome-2 | A | 3140 | G |
| Brain Stem | 0.0883 | Genome-2 | Genome-2 | A | 3140 | G |
| Occipital Lobe | 0.881 | Genome-2 | Genome-2 | A | 3140 | G |
| SSPE 1 | 0.3497 | Genome-2 | Genome-2 | A | 3140 | G |
| Cerebellum | 0.0787 | Genome-2 | Genome-2 | A | 3140 | G |
| Cerebellum Nucleus | 0.2552 | Genome-2 | Genome-2 | A | 3140 | G |
| Frontal Cortex 2 | 0.3806 | Genome-2 | Genome-2 | A | 3140 | G |
| Internal Capsule | 0.5527 | Genome-2 | Genome-2 | A | 3140 | G |
| Frontal Cortex 3 | 0.317 | Genome-2 | Genome-2 | A | 3140 | G |
| Hippocampus | 0.7495 | Genome-2 | Genome-2 | A | 3140 | G |
| Temporal Lobe | 0.0758 | Genome-1 | Genome-01 | C | 3220 | T |
| Parietal Lobe | 0.6568 | Genome-1 | Genome-01 | C | 3220 | T |
| Upper Brain Stem | 0.8003 | Genome-1 | Genome-01 | C | 3220 | T |
| Midbrain | 0.738 | Genome-1 | Genome-01 | C | 3220 | T |
| Frontal Cortex 1 | 0.6563 | Genome-1 | Genome-01 | C | 3220 | T |
| SSPE 2 | 0.6934 | Genome-1 | Genome-01 | C | 3220 | T |
| Brain Stem | 0.8911 | Genome-1 | Genome-01 | C | 3220 | T |
| Occipital Lobe | 0.0761 | Genome-1 | Genome-01 | C | 3220 | T |
| SSPE 1 | 0.62 | Genome-1 | Genome-01 | C | 3220 | T |
| Cerebellum | 0.9078 | Genome-1 | Genome-01 | C | 3220 | T |
| Cerebellum Nucleus | 0.7105 | Genome-1 | Genome-01 | C | 3220 | T |
| Frontal Cortex 2 | 0.5669 | Genome-1 | Genome-01 | C | 3220 | T |
| Internal Capsule | 0.398 | Genome-1 | Genome-01 | C | 3220 | T |
| Frontal Cortex 3 | 0.6887 | Genome-1 | Genome-01 | C | 3220 | T |
| Hippocampus | 0.217 | Genome-1 | Genome-01 | C | 3220 | T |
| Temporal Lobe | 0.1108 | Genome-1 | Genome-01 | T | 3393 | C |
| Parietal Lobe | 0.6705 | Genome-1 | Genome-01 | T | 3393 | C |
| Upper Brain Stem | 0.8395 | Genome-1 | Genome-01 | T | 3393 | C |
| Midbrain | 0.7918 | Genome-1 | Genome-01 | T | 3393 | C |
| Frontal Cortex 1 | 0.7089 | Genome-1 | Genome-01 | T | 3393 | C |
| SSPE 2 | 0.8312 | Genome-1 | Genome-01 | T | 3393 | C |
| Brain Stem | 0.9334 | Genome-1 | Genome-01 | T | 3393 | C |
| Occipital Lobe | 0.103 | Genome-1 | Genome-01 | T | 3393 | C |
| SSPE 1 | 0.8014 | Genome-1 | Genome-01 | T | 3393 | C |
| Cerebellum | 0.9162 | Genome-1 | Genome-01 | T | 3393 | C |
| Cerebellum Nucleus | 0.752 | Genome-1 | Genome-01 | T | 3393 | C |
| Frontal Cortex 2 | 0.5666 | Genome-1 | Genome-01 | T | 3393 | C |
| Internal Capsule | 0.4629 | Genome-1 | Genome-01 | T | 3393 | C |
| Frontal Cortex 3 | 0.7056 | Genome-1 | Genome-01 | T | 3393 | C |
| Hippocampus | 0.2246 | Genome-1 | Genome-01 | T | 3393 | C |
| Temporal Lobe | 0.114 | Genome-1 | Genome-01 | T | 3396 | C |
| Parietal Lobe | 0.6771 | Genome-1 | Genome-01 | T | 3396 | C |
| Upper Brain Stem | 0.8465 | Genome-1 | Genome-01 | T | 3396 | C |
| Midbrain | 0.7999 | Genome-1 | Genome-01 | T | 3396 | C |
| Frontal Cortex 1 | 0.7167 | Genome-1 | Genome-01 | T | 3396 | C |
| SSPE 2 | 0.8564 | Genome-1 | Genome-01 | T | 3396 | C |
| Brain Stem | 0.9354 | Genome-1 | Genome-01 | T | 3396 | C |
| Occipital Lobe | 0.1099 | Genome-1 | Genome-01 | T | 3396 | C |
| SSPE 1 | 0.8447 | Genome-1 | Genome-01 | T | 3396 | C |
| Cerebellum | 0.9325 | Genome-1 | Genome-01 | T | 3396 | C |
| Cerebellum Nucleus | 0.7624 | Genome-1 | Genome-01 | T | 3396 | C |
| Frontal Cortex 2 | 0.5736 | Genome-1 | Genome-01 | T | 3396 | C |
| Internal Capsule | 0.4743 | Genome-1 | Genome-01 | T | 3396 | C |
| Frontal Cortex 3 | 0.7176 | Genome-1 | Genome-01 | T | 3396 | C |
| Hippocampus | 0.2314 | Genome-1 | Genome-01 | T | 3396 | C |
| Temporal Lobe | 0.1052 | Genome-1 | Genome-1 | T | 3586 | C |
| Parietal Lobe | 0.5911 | Genome-1 | Genome-1 | T | 3586 | C |
| Upper Brain Stem | 0.7815 | Genome-1 | Genome-1 | T | 3586 | C |
| Midbrain | 0.7621 | Genome-1 | Genome-1 | T | 3586 | C |
| Frontal Cortex 1 | 0.6146 | Genome-1 | Genome-1 | T | 3586 | C |
| SSPE 2 | 0.7172 | Genome-1 | Genome-1 | T | 3586 | C |
| Brain Stem | 0.9107 | Genome-1 | Genome-1 | T | 3586 | C |
| Occipital Lobe | 0.0918 | Genome-1 | Genome-1 | T | 3586 | C |
| SSPE 1 | 0.6695 | Genome-1 | Genome-1 | T | 3586 | C |
| Cerebellum | 0.8824 | Genome-1 | Genome-1 | T | 3586 | C |
| Cerebellum Nucleus | 0.6768 | Genome-1 | Genome-1 | T | 3586 | C |
| Frontal Cortex 2 | 0.0874 | Genome-1 | Genome-1 | T | 3586 | C |
| Internal Capsule | 0.3112 | Genome-1 | Genome-1 | T | 3586 | C |
| Frontal Cortex 3 | 0.6086 | Genome-1 | Genome-1 | T | 3586 | C |
| Hippocampus | 0.1725 | Genome-1 | Genome-1 | T | 3586 | C |
| Temporal Lobe | 0.0604 | Genome-1 | Genome-1 | T | 3802 | C |
| Parietal Lobe | 0.5149 | Genome-1 | Genome-1 | T | 3802 | C |
| Upper Brain Stem | 0.7493 | Genome-1 | Genome-1 | T | 3802 | C |
| Midbrain | 0.7052 | Genome-1 | Genome-1 | T | 3802 | C |
| Frontal Cortex 1 | 0.5528 | Genome-1 | Genome-1 | T | 3802 | C |
| SSPE 2 | 0.7056 | Genome-1 | Genome-1 | T | 3802 | C |
| Brain Stem | 0.8715 | Genome-1 | Genome-1 | T | 3802 | C |
| Occipital Lobe | 0.0417 | Genome-1 | Genome-1 | T | 3802 | C |
| SSPE 1 | 0.6408 | Genome-1 | Genome-1 | T | 3802 | C |
| Cerebellum | 0.8397 | Genome-1 | Genome-1 | T | 3802 | C |
| Cerebellum Nucleus | 0.6125 | Genome-1 | Genome-1 | T | 3802 | C |
| Frontal Cortex 2 | 0.022432 | Genome-1 | Genome-1 | T | 3802 | C |
| Internal Capsule | 0.302 | Genome-1 | Genome-1 | T | 3802 | C |
| Frontal Cortex 3 | 0.5764 | Genome-1 | Genome-1 | T | 3802 | C |
| Hippocampus | 0.1645 | Genome-1 | Genome-1 | T | 3802 | C |
| Temporal Lobe | 0.1104 | Genome-1 | Genome-1 | T | 3869 | C |
| Parietal Lobe | 0.5518 | Genome-1 | Genome-1 | T | 3869 | C |
| Upper Brain Stem | 0.775 | Genome-1 | Genome-1 | T | 3869 | C |
| Midbrain | 0.7149 | Genome-1 | Genome-1 | T | 3869 | C |
| Frontal Cortex 1 | 0.6159 | Genome-1 | Genome-1 | T | 3869 | C |
| SSPE 2 | 0.7421 | Genome-1 | Genome-1 | T | 3869 | C |
| Brain Stem | 0.9047 | Genome-1 | Genome-1 | T | 3869 | C |
| Occipital Lobe | 0.1141 | Genome-1 | Genome-1 | T | 3869 | C |
| SSPE 1 | 0.6922 | Genome-1 | Genome-1 | T | 3869 | C |
| Cerebellum | 0.8569 | Genome-1 | Genome-1 | T | 3869 | C |
| Cerebellum Nucleus | 0.6199 | Genome-1 | Genome-1 | T | 3869 | C |
| Frontal Cortex 2 | 0.1278 | Genome-1 | Genome-1 | T | 3869 | C |
| Internal Capsule | 0.3608 | Genome-1 | Genome-1 | T | 3869 | C |
| Frontal Cortex 3 | 0.5966 | Genome-1 | Genome-1 | T | 3869 | C |
| Hippocampus | 0.1992 | Genome-1 | Genome-1 | T | 3869 | C |
| Temporal Lobe | 0.1036 | Genome-1 | Genome-01 | T | 3907 | C |
| Parietal Lobe | 0.5565 | Genome-1 | Genome-01 | T | 3907 | C |
| Upper Brain Stem | 0.8076 | Genome-1 | Genome-01 | T | 3907 | C |
| Midbrain | 0.7356 | Genome-1 | Genome-01 | T | 3907 | C |
| Frontal Cortex 1 | 0.688 | Genome-1 | Genome-01 | T | 3907 | C |
| SSPE 2 | 0.7425 | Genome-1 | Genome-01 | T | 3907 | C |
| Brain Stem | 0.9197 | Genome-1 | Genome-01 | T | 3907 | C |
| Occipital Lobe | 0.0817 | Genome-1 | Genome-01 | T | 3907 | C |
| SSPE 1 | 0.6964 | Genome-1 | Genome-01 | T | 3907 | C |
| Cerebellum | 0.8677 | Genome-1 | Genome-01 | T | 3907 | C |
| Cerebellum Nucleus | 0.6945 | Genome-1 | Genome-01 | T | 3907 | C |
| Frontal Cortex 2 | 0.4619 | Genome-1 | Genome-01 | T | 3907 | C |
| Internal Capsule | 0.3999 | Genome-1 | Genome-01 | T | 3907 | C |
| Frontal Cortex 3 | 0.6659 | Genome-1 | Genome-01 | T | 3907 | C |
| Hippocampus | 0.2005 | Genome-1 | Genome-01 | T | 3907 | C |
| Temporal Lobe | 0.0918 | Genome-1 | Genome-01 | T | 3908 | C |
| Parietal Lobe | 0.5486 | Genome-1 | Genome-01 | T | 3908 | C |
| Upper Brain Stem | 0.7876 | Genome-1 | Genome-01 | T | 3908 | C |
| Midbrain | 0.7272 | Genome-1 | Genome-01 | T | 3908 | C |
| Frontal Cortex 1 | 0.6384 | Genome-1 | Genome-01 | T | 3908 | C |
| SSPE 2 | 0.7378 | Genome-1 | Genome-01 | T | 3908 | C |
| Brain Stem | 0.9014 | Genome-1 | Genome-01 | T | 3908 | C |
| Occipital Lobe | 0.0741 | Genome-1 | Genome-01 | T | 3908 | C |
| SSPE 1 | 0.6904 | Genome-1 | Genome-01 | T | 3908 | C |
| Cerebellum | 0.8463 | Genome-1 | Genome-01 | T | 3908 | C |
| Cerebellum Nucleus | 0.6709 | Genome-1 | Genome-01 | T | 3908 | C |
| Frontal Cortex 2 | 0.4534 | Genome-1 | Genome-01 | T | 3908 | C |
| Internal Capsule | 0.3903 | Genome-1 | Genome-01 | T | 3908 | C |
| Frontal Cortex 3 | 0.6124 | Genome-1 | Genome-01 | T | 3908 | C |
| Hippocampus | 0.1934 | Genome-1 | Genome-01 | T | 3908 | C |
| Temporal Lobe | 0.0933 | Genome-1 | Genome-01 | T | 3912 | C |
| Parietal Lobe | 0.558 | Genome-1 | Genome-01 | T | 3912 | C |
| Upper Brain Stem | 0.8065 | Genome-1 | Genome-01 | T | 3912 | C |
| Midbrain | 0.7367 | Genome-1 | Genome-01 | T | 3912 | C |
| Frontal Cortex 1 | 0.6859 | Genome-1 | Genome-01 | T | 3912 | C |
| SSPE 2 | 0.7387 | Genome-1 | Genome-01 | T | 3912 | C |
| Brain Stem | 0.9155 | Genome-1 | Genome-01 | T | 3912 | C |
| Occipital Lobe | 0.0849 | Genome-1 | Genome-01 | T | 3912 | C |
| SSPE 1 | 0.6929 | Genome-1 | Genome-01 | T | 3912 | C |
| Cerebellum | 0.8603 | Genome-1 | Genome-01 | T | 3912 | C |
| Cerebellum Nucleus | 0.6931 | Genome-1 | Genome-01 | T | 3912 | C |
| Frontal Cortex 2 | 0.4658 | Genome-1 | Genome-01 | T | 3912 | C |
| Internal Capsule | 0.4005 | Genome-1 | Genome-01 | T | 3912 | C |
| Frontal Cortex 3 | 0.6651 | Genome-1 | Genome-01 | T | 3912 | C |
| Hippocampus | 0.2038 | Genome-1 | Genome-01 | T | 3912 | C |
| Temporal Lobe | 0.1265 | Genome-1 | Genome-01 | T | 3938 | C |
| Parietal Lobe | 0.6047 | Genome-1 | Genome-01 | T | 3938 | C |
| Upper Brain Stem | 0.8461 | Genome-1 | Genome-01 | T | 3938 | C |
| Midbrain | 0.7812 | Genome-1 | Genome-01 | T | 3938 | C |
| Frontal Cortex 1 | 0.6835 | Genome-1 | Genome-01 | T | 3938 | C |
| SSPE 2 | 0.7737 | Genome-1 | Genome-01 | T | 3938 | C |
| Brain Stem | 0.9336 | Genome-1 | Genome-01 | T | 3938 | C |
| Occipital Lobe | 0.1045 | Genome-1 | Genome-01 | T | 3938 | C |
| SSPE 1 | 0.7228 | Genome-1 | Genome-01 | T | 3938 | C |
| Cerebellum | 0.9089 | Genome-1 | Genome-01 | T | 3938 | C |
| Cerebellum Nucleus | 0.7441 | Genome-1 | Genome-01 | T | 3938 | C |
| Frontal Cortex 2 | 0.5226 | Genome-1 | Genome-01 | T | 3938 | C |
| Internal Capsule | 0.4563 | Genome-1 | Genome-01 | T | 3938 | C |
| Frontal Cortex 3 | 0.6586 | Genome-1 | Genome-01 | T | 3938 | C |
| Hippocampus | 0.2385 | Genome-1 | Genome-01 | T | 3938 | C |
| Temporal Lobe | 0.0997 | Genome-1 | Genome-01 | T | 3945 | C |
| Parietal Lobe | 0.5568 | Genome-1 | Genome-01 | T | 3945 | C |
| Upper Brain Stem | 0.8179 | Genome-1 | Genome-01 | T | 3945 | C |
| Midbrain | 0.748 | Genome-1 | Genome-01 | T | 3945 | C |
| Frontal Cortex 1 | 0.7005 | Genome-1 | Genome-01 | T | 3945 | C |
| SSPE 2 | 0.7384 | Genome-1 | Genome-01 | T | 3945 | C |
| Brain Stem | 0.9223 | Genome-1 | Genome-01 | T | 3945 | C |
| Occipital Lobe | 0.0868 | Genome-1 | Genome-01 | T | 3945 | C |
| SSPE 1 | 0.6803 | Genome-1 | Genome-01 | T | 3945 | C |
| Cerebellum | 0.8861 | Genome-1 | Genome-01 | T | 3945 | C |
| Cerebellum Nucleus | 0.7035 | Genome-1 | Genome-01 | T | 3945 | C |
| Frontal Cortex 2 | 0.4743 | Genome-1 | Genome-01 | T | 3945 | C |
| Internal Capsule | 0.4016 | Genome-1 | Genome-01 | T | 3945 | C |
| Frontal Cortex 3 | 0.6675 | Genome-1 | Genome-01 | T | 3945 | C |
| Hippocampus | 0.1997 | Genome-1 | Genome-01 | T | 3945 | C |
| Temporal Lobe | 0.1054 | Genome-1 | Genome-01 | T | 3947 | C |
| Parietal Lobe | 0.5521 | Genome-1 | Genome-01 | T | 3947 | C |
| Upper Brain Stem | 0.8145 | Genome-1 | Genome-01 | T | 3947 | C |
| Midbrain | 0.7446 | Genome-1 | Genome-01 | T | 3947 | C |
| Frontal Cortex 1 | 0.697 | Genome-1 | Genome-01 | T | 3947 | C |
| SSPE 2 | 0.7375 | Genome-1 | Genome-01 | T | 3947 | C |
| Brain Stem | 0.9213 | Genome-1 | Genome-01 | T | 3947 | C |
| Occipital Lobe | 0.0841 | Genome-1 | Genome-01 | T | 3947 | C |
| SSPE 1 | 0.6787 | Genome-1 | Genome-01 | T | 3947 | C |
| Cerebellum | 0.8809 | Genome-1 | Genome-01 | T | 3947 | C |
| Cerebellum Nucleus | 0.7051 | Genome-1 | Genome-01 | T | 3947 | C |
| Frontal Cortex 2 | 0.464 | Genome-1 | Genome-01 | T | 3947 | C |
| Internal Capsule | 0.3964 | Genome-1 | Genome-01 | T | 3947 | C |
| Frontal Cortex 3 | 0.6626 | Genome-1 | Genome-01 | T | 3947 | C |
| Hippocampus | 0.197 | Genome-1 | Genome-01 | T | 3947 | C |
| Temporal Lobe | 0.1026 | Genome-1 | Genome-01 | T | 3948 | C |
| Parietal Lobe | 0.5493 | Genome-1 | Genome-01 | T | 3948 | C |
| Upper Brain Stem | 0.8115 | Genome-1 | Genome-01 | T | 3948 | C |
| Midbrain | 0.7438 | Genome-1 | Genome-01 | T | 3948 | C |
| Frontal Cortex 1 | 0.6439 | Genome-1 | Genome-01 | T | 3948 | C |
| SSPE 2 | 0.7353 | Genome-1 | Genome-01 | T | 3948 | C |
| Brain Stem | 0.9211 | Genome-1 | Genome-01 | T | 3948 | C |
| Occipital Lobe | 0.0834 | Genome-1 | Genome-01 | T | 3948 | C |
| SSPE 1 | 0.6733 | Genome-1 | Genome-01 | T | 3948 | C |
| Cerebellum | 0.8822 | Genome-1 | Genome-01 | T | 3948 | C |
| Cerebellum Nucleus | 0.7023 | Genome-1 | Genome-01 | T | 3948 | C |
| Frontal Cortex 2 | 0.4624 | Genome-1 | Genome-01 | T | 3948 | C |
| Internal Capsule | 0.3916 | Genome-1 | Genome-01 | T | 3948 | C |
| Frontal Cortex 3 | 0.6068 | Genome-1 | Genome-01 | T | 3948 | C |
| Hippocampus | 0.1898 | Genome-1 | Genome-01 | T | 3948 | C |
| Temporal Lobe | 0.0827 | Genome-1 | Genome-01 | T | 4024 | C |
| Parietal Lobe | 0.541 | Genome-1 | Genome-01 | T | 4024 | C |
| Upper Brain Stem | 0.787 | Genome-1 | Genome-01 | T | 4024 | C |
| Midbrain | 0.722 | Genome-1 | Genome-01 | T | 4024 | C |
| Frontal Cortex 1 | 0.6354 | Genome-1 | Genome-01 | T | 4024 | C |
| SSPE 2 | 0.7248 | Genome-1 | Genome-01 | T | 4024 | C |
| Brain Stem | 0.9057 | Genome-1 | Genome-01 | T | 4024 | C |
| Occipital Lobe | 0.0804 | Genome-1 | Genome-01 | T | 4024 | C |
| SSPE 1 | 0.6782 | Genome-1 | Genome-01 | T | 4024 | C |
| Cerebellum | 0.8351 | Genome-1 | Genome-01 | T | 4024 | C |
| Cerebellum Nucleus | 0.6685 | Genome-1 | Genome-01 | T | 4024 | C |
| Frontal Cortex 2 | 0.48 | Genome-1 | Genome-01 | T | 4024 | C |
| Internal Capsule | 0.3749 | Genome-1 | Genome-01 | T | 4024 | C |
| Frontal Cortex 3 | 0.591 | Genome-1 | Genome-01 | T | 4024 | C |
| Hippocampus | 0.1772 | Genome-1 | Genome-01 | T | 4024 | C |
| Temporal Lobe | 0.0767 | Genome-1 | Genome-1 | T | 4087 | C |
| Parietal Lobe | 0.5373 | Genome-1 | Genome-1 | T | 4087 | C |
| Upper Brain Stem | 0.6905 | Genome-1 | Genome-1 | T | 4087 | C |
| Midbrain | 0.6669 | Genome-1 | Genome-1 | T | 4087 | C |
| Frontal Cortex 1 | 0.6065 | Genome-1 | Genome-1 | T | 4087 | C |
| SSPE 2 | 0.6643 | Genome-1 | Genome-1 | T | 4087 | C |
| Brain Stem | 0.877 | Genome-1 | Genome-1 | T | 4087 | C |
| Occipital Lobe | 0.058 | Genome-1 | Genome-1 | T | 4087 | C |
| SSPE 1 | 0.6254 | Genome-1 | Genome-1 | T | 4087 | C |
| Cerebellum | 0.7761 | Genome-1 | Genome-1 | T | 4087 | C |
| Cerebellum Nucleus | 0.5486 | Genome-1 | Genome-1 | T | 4087 | C |
| Frontal Cortex 2 | 0.0365 | Genome-1 | Genome-1 | T | 4087 | C |
| Internal Capsule | 0.317 | Genome-1 | Genome-1 | T | 4087 | C |
| Frontal Cortex 3 | 0.5759 | Genome-1 | Genome-1 | T | 4087 | C |
| Hippocampus | 0.1574 | Genome-1 | Genome-1 | T | 4087 | C |
| Temporal Lobe | 0.0814 | Genome-1 | Genome-01 | T | 4088 | C |
| Parietal Lobe | 0.5542 | Genome-1 | Genome-01 | T | 4088 | C |
| Upper Brain Stem | 0.6964 | Genome-1 | Genome-01 | T | 4088 | C |
| Midbrain | 0.6697 | Genome-1 | Genome-01 | T | 4088 | C |
| Frontal Cortex 1 | 0.6312 | Genome-1 | Genome-01 | T | 4088 | C |
| SSPE 2 | 0.6741 | Genome-1 | Genome-01 | T | 4088 | C |
| Brain Stem | 0.877 | Genome-1 | Genome-01 | T | 4088 | C |
| Occipital Lobe | 0.076 | Genome-1 | Genome-01 | T | 4088 | C |
| SSPE 1 | 0.6268 | Genome-1 | Genome-01 | T | 4088 | C |
| Cerebellum | 0.8075 | Genome-1 | Genome-01 | T | 4088 | C |
| Cerebellum Nucleus | 0.6003 | Genome-1 | Genome-01 | T | 4088 | C |
| Frontal Cortex 2 | 0.4701 | Genome-1 | Genome-01 | T | 4088 | C |
| Internal Capsule | 0.3679 | Genome-1 | Genome-01 | T | 4088 | C |
| Frontal Cortex 3 | 0.6002 | Genome-1 | Genome-01 | T | 4088 | C |
| Hippocampus | 0.169 | Genome-1 | Genome-01 | T | 4088 | C |
| Temporal Lobe | 0.0659 | Genome-1 | Genome-01 | T | 4090 | C |
| Parietal Lobe | 0.5458 | Genome-1 | Genome-01 | T | 4090 | C |
| Upper Brain Stem | 0.7176 | Genome-1 | Genome-01 | T | 4090 | C |
| Midbrain | 0.6705 | Genome-1 | Genome-01 | T | 4090 | C |
| Frontal Cortex 1 | 0.5834 | Genome-1 | Genome-01 | T | 4090 | C |
| SSPE 2 | 0.6709 | Genome-1 | Genome-01 | T | 4090 | C |
| Brain Stem | 0.8819 | Genome-1 | Genome-01 | T | 4090 | C |
| Occipital Lobe | 0.068 | Genome-1 | Genome-01 | T | 4090 | C |
| SSPE 1 | 0.6233 | Genome-1 | Genome-01 | T | 4090 | C |
| Cerebellum | 0.8106 | Genome-1 | Genome-01 | T | 4090 | C |
| Cerebellum Nucleus | 0.5966 | Genome-1 | Genome-01 | T | 4090 | C |
| Frontal Cortex 2 | 0.4562 | Genome-1 | Genome-01 | T | 4090 | C |
| Internal Capsule | 0.3566 | Genome-1 | Genome-01 | T | 4090 | C |
| Frontal Cortex 3 | 0.5482 | Genome-1 | Genome-01 | T | 4090 | C |
| Hippocampus | 0.1479 | Genome-1 | Genome-01 | T | 4090 | C |
| Temporal Lobe | 0.0211 | Genome-1 | Genome-FC2 | T | 4216 | C |
| Parietal Lobe | 0.0308 | Genome-1 | Genome-FC2 | T | 4216 | C |
| Upper Brain Stem | 0.0604 | Genome-1 | Genome-FC2 | T | 4216 | C |
| Midbrain | 0 | Genome-1 | Genome-FC2 | T | 4216 | C |
| Frontal Cortex 1 | 0.0424 | Genome-1 | Genome-FC2 | T | 4216 | C |
| SSPE 2 | 0.0289 | Genome-1 | Genome-FC2 | T | 4216 | C |
| Brain Stem | 0.0336 | Genome-1 | Genome-FC2 | T | 4216 | C |
| Occipital Lobe | 0.0488 | Genome-1 | Genome-FC2 | T | 4216 | C |
| SSPE 1 | 0.0414 | Genome-1 | Genome-FC2 | T | 4216 | C |
| Cerebellum | 0.0305 | Genome-1 | Genome-FC2 | T | 4216 | C |
| Cerebellum Nucleus | 0.0219 | Genome-1 | Genome-FC2 | T | 4216 | C |
| Frontal Cortex 2 | 0.5491 | Genome-1 | Genome-FC2 | T | 4216 | C |
| Internal Capsule | 0.0406 | Genome-1 | Genome-FC2 | T | 4216 | C |
| Frontal Cortex 3 | 0.044 | Genome-1 | Genome-FC2 | T | 4216 | C |
| Hippocampus | 0.0239 | Genome-1 | Genome-FC2 | T | 4216 | C |
| Temporal Lobe | 0.152 | Genome-1 | Genome-01 | T | 4275 | C |
| Parietal Lobe | 0.6038 | Genome-1 | Genome-01 | T | 4275 | C |
| Upper Brain Stem | 0.801 | Genome-1 | Genome-01 | T | 4275 | C |
| Midbrain | 0.7318 | Genome-1 | Genome-01 | T | 4275 | C |
| Frontal Cortex 1 | 0.687 | Genome-1 | Genome-01 | T | 4275 | C |
| SSPE 2 | 0.7253 | Genome-1 | Genome-01 | T | 4275 | C |
| Brain Stem | 0.9124 | Genome-1 | Genome-01 | T | 4275 | C |
| Occipital Lobe | 0.0915 | Genome-1 | Genome-01 | T | 4275 | C |
| SSPE 1 | 0.6692 | Genome-1 | Genome-01 | T | 4275 | C |
| Cerebellum | 0.9098 | Genome-1 | Genome-01 | T | 4275 | C |
| Cerebellum Nucleus | 0.7046 | Genome-1 | Genome-01 | T | 4275 | C |
| Frontal Cortex 2 | 0.564 | Genome-1 | Genome-01 | T | 4275 | C |
| Internal Capsule | 0.434 | Genome-1 | Genome-01 | T | 4275 | C |
| Frontal Cortex 3 | 0.6828 | Genome-1 | Genome-01 | T | 4275 | C |
| Hippocampus | 0.2238 | Genome-1 | Genome-01 | T | 4275 | C |
| Temporal Lobe | 0.1244 | Genome-1 | Genome-01 | T | 4276 | C |
| Parietal Lobe | 0.601 | Genome-1 | Genome-01 | T | 4276 | C |
| Upper Brain Stem | 0.8002 | Genome-1 | Genome-01 | T | 4276 | C |
| Midbrain | 0.7307 | Genome-1 | Genome-01 | T | 4276 | C |
| Frontal Cortex 1 | 0.6434 | Genome-1 | Genome-01 | T | 4276 | C |
| SSPE 2 | 0.7238 | Genome-1 | Genome-01 | T | 4276 | C |
| Brain Stem | 0.9115 | Genome-1 | Genome-01 | T | 4276 | C |
| Occipital Lobe | 0.0822 | Genome-1 | Genome-01 | T | 4276 | C |
| SSPE 1 | 0.6671 | Genome-1 | Genome-01 | T | 4276 | C |
| Cerebellum | 0.9112 | Genome-1 | Genome-01 | T | 4276 | C |
| Cerebellum Nucleus | 0.6984 | Genome-1 | Genome-01 | T | 4276 | C |
| Frontal Cortex 2 | 0.5589 | Genome-1 | Genome-01 | T | 4276 | C |
| Internal Capsule | 0.4257 | Genome-1 | Genome-01 | T | 4276 | C |
| Frontal Cortex 3 | 0.6428 | Genome-1 | Genome-01 | T | 4276 | C |
| Hippocampus | 0.212 | Genome-1 | Genome-01 | T | 4276 | C |
| Temporal Lobe | 0.4322 | Genome-2 | cluster 9 | A | 4304 | G |
| Parietal Lobe | 0 | Genome-2 | cluster 9 | A | 4304 | G |
| Upper Brain Stem | 0.0501 | Genome-2 | cluster 9 | A | 4304 | G |
| Midbrain | 0.021176 | Genome-2 | cluster 9 | A | 4304 | G |
| Frontal Cortex 1 | 0 | Genome-2 | cluster 9 | A | 4304 | G |
| SSPE 2 | 0 | Genome-2 | cluster 9 | A | 4304 | G |
| Brain Stem | 0.020145 | Genome-2 | cluster 9 | A | 4304 | G |
| Occipital Lobe | 0.224 | Genome-2 | cluster 9 | A | 4304 | G |
| SSPE 1 | 0 | Genome-2 | cluster 9 | A | 4304 | G |
| Cerebellum | 0 | Genome-2 | cluster 9 | A | 4304 | G |
| Cerebellum Nucleus | 0 | Genome-2 | cluster 9 | A | 4304 | G |
| Frontal Cortex 2 | 0 | Genome-2 | cluster 9 | A | 4304 | G |
| Internal Capsule | 0.02 | Genome-2 | cluster 9 | A | 4304 | G |
| Frontal Cortex 3 | 0 | Genome-2 | cluster 9 | A | 4304 | G |
| Hippocampus | 0 | Genome-2 | cluster 9 | A | 4304 | G |
| Temporal Lobe | 0.1028 | Genome-1 | Genome-01 | T | 4309 | C |
| Parietal Lobe | 0.629 | Genome-1 | Genome-01 | T | 4309 | C |
| Upper Brain Stem | 0.8 | Genome-1 | Genome-01 | T | 4309 | C |
| Midbrain | 0.7425 | Genome-1 | Genome-01 | T | 4309 | C |
| Frontal Cortex 1 | 0.6309 | Genome-1 | Genome-01 | T | 4309 | C |
| SSPE 2 | 0.7208 | Genome-1 | Genome-01 | T | 4309 | C |
| Brain Stem | 0.9078 | Genome-1 | Genome-01 | T | 4309 | C |
| Occipital Lobe | 0.09 | Genome-1 | Genome-01 | T | 4309 | C |
| SSPE 1 | 0.6762 | Genome-1 | Genome-01 | T | 4309 | C |
| Cerebellum | 0.905 | Genome-1 | Genome-01 | T | 4309 | C |
| Cerebellum Nucleus | 0.6965 | Genome-1 | Genome-01 | T | 4309 | C |
| Frontal Cortex 2 | 0.5751 | Genome-1 | Genome-01 | T | 4309 | C |
| Internal Capsule | 0.4495 | Genome-1 | Genome-01 | T | 4309 | C |
| Frontal Cortex 3 | 0.6346 | Genome-1 | Genome-01 | T | 4309 | C |
| Hippocampus | 0.2244 | Genome-1 | Genome-01 | T | 4309 | C |
| Temporal Lobe | 0.1143 | Genome-1 | Genome-01 | T | 4310 | C |
| Parietal Lobe | 0.6252 | Genome-1 | Genome-01 | T | 4310 | C |
| Upper Brain Stem | 0.8011 | Genome-1 | Genome-01 | T | 4310 | C |
| Midbrain | 0.7592 | Genome-1 | Genome-01 | T | 4310 | C |
| Frontal Cortex 1 | 0.6347 | Genome-1 | Genome-01 | T | 4310 | C |
| SSPE 2 | 0.7213 | Genome-1 | Genome-01 | T | 4310 | C |
| Brain Stem | 0.909 | Genome-1 | Genome-01 | T | 4310 | C |
| Occipital Lobe | 0.0932 | Genome-1 | Genome-01 | T | 4310 | C |
| SSPE 1 | 0.6773 | Genome-1 | Genome-01 | T | 4310 | C |
| Cerebellum | 0.9104 | Genome-1 | Genome-01 | T | 4310 | C |
| Cerebellum Nucleus | 0.7005 | Genome-1 | Genome-01 | T | 4310 | C |
| Frontal Cortex 2 | 0.5714 | Genome-1 | Genome-01 | T | 4310 | C |
| Internal Capsule | 0.4442 | Genome-1 | Genome-01 | T | 4310 | C |
| Frontal Cortex 3 | 0.638 | Genome-1 | Genome-01 | T | 4310 | C |
| Hippocampus | 0.2278 | Genome-1 | Genome-01 | T | 4310 | C |
| Temporal Lobe | 0.1157 | Genome-1 | Genome-1 | T | 4345 | C |
| Parietal Lobe | 0.641 | Genome-1 | Genome-1 | T | 4345 | C |
| Upper Brain Stem | 0.7749 | Genome-1 | Genome-1 | T | 4345 | C |
| Midbrain | 0.7225 | Genome-1 | Genome-1 | T | 4345 | C |
| Frontal Cortex 1 | 0.6447 | Genome-1 | Genome-1 | T | 4345 | C |
| SSPE 2 | 0.7043 | Genome-1 | Genome-1 | T | 4345 | C |
| Brain Stem | 0.907 | Genome-1 | Genome-1 | T | 4345 | C |
| Occipital Lobe | 0.0941 | Genome-1 | Genome-1 | T | 4345 | C |
| SSPE 1 | 0.6716 | Genome-1 | Genome-1 | T | 4345 | C |
| Cerebellum | 0.8767 | Genome-1 | Genome-1 | T | 4345 | C |
| Cerebellum Nucleus | 0.6627 | Genome-1 | Genome-1 | T | 4345 | C |
| Frontal Cortex 2 | 0.0606 | Genome-1 | Genome-1 | T | 4345 | C |
| Internal Capsule | 0.4324 | Genome-1 | Genome-1 | T | 4345 | C |
| Frontal Cortex 3 | 0.6209 | Genome-1 | Genome-1 | T | 4345 | C |
| Hippocampus | 0.216 | Genome-1 | Genome-1 | T | 4345 | C |
| Temporal Lobe | 0.0931 | Genome-1 | Genome-01 | T | 4361 | C |
| Parietal Lobe | 0.6318 | Genome-1 | Genome-01 | T | 4361 | C |
| Upper Brain Stem | 0.7808 | Genome-1 | Genome-01 | T | 4361 | C |
| Midbrain | 0.7272 | Genome-1 | Genome-01 | T | 4361 | C |
| Frontal Cortex 1 | 0.6476 | Genome-1 | Genome-01 | T | 4361 | C |
| SSPE 2 | 0.7063 | Genome-1 | Genome-01 | T | 4361 | C |
| Brain Stem | 0.9137 | Genome-1 | Genome-01 | T | 4361 | C |
| Occipital Lobe | 0.0918 | Genome-1 | Genome-01 | T | 4361 | C |
| SSPE 1 | 0.6715 | Genome-1 | Genome-01 | T | 4361 | C |
| Cerebellum | 0.8853 | Genome-1 | Genome-01 | T | 4361 | C |
| Cerebellum Nucleus | 0.6705 | Genome-1 | Genome-01 | T | 4361 | C |
| Frontal Cortex 2 | 0.5314 | Genome-1 | Genome-01 | T | 4361 | C |
| Internal Capsule | 0.4309 | Genome-1 | Genome-01 | T | 4361 | C |
| Frontal Cortex 3 | 0.6215 | Genome-1 | Genome-01 | T | 4361 | C |
| Hippocampus | 0.2047 | Genome-1 | Genome-01 | T | 4361 | C |
| Temporal Lobe | 0.0829 | Genome-1 | Genome-1 | T | 4441 | C |
| Parietal Lobe | 0.5692 | Genome-1 | Genome-1 | T | 4441 | C |
| Upper Brain Stem | 0.7783 | Genome-1 | Genome-1 | T | 4441 | C |
| Midbrain | 0.7095 | Genome-1 | Genome-1 | T | 4441 | C |
| Frontal Cortex 1 | 0.6339 | Genome-1 | Genome-1 | T | 4441 | C |
| SSPE 2 | 0.6994 | Genome-1 | Genome-1 | T | 4441 | C |
| Brain Stem | 0.9004 | Genome-1 | Genome-1 | T | 4441 | C |
| Occipital Lobe | 0.0587 | Genome-1 | Genome-1 | T | 4441 | C |
| SSPE 1 | 0.6606 | Genome-1 | Genome-1 | T | 4441 | C |
| Cerebellum | 0.8753 | Genome-1 | Genome-1 | T | 4441 | C |
| Cerebellum Nucleus | 0.6378 | Genome-1 | Genome-1 | T | 4441 | C |
| Frontal Cortex 2 | 0.03 | Genome-1 | Genome-1 | T | 4441 | C |
| Internal Capsule | 0.3852 | Genome-1 | Genome-1 | T | 4441 | C |
| Frontal Cortex 3 | 0.5816 | Genome-1 | Genome-1 | T | 4441 | C |
| Hippocampus | 0.195 | Genome-1 | Genome-1 | T | 4441 | C |
| Temporal Lobe | 0.9024 | Genome-2 | Genome-2 | C | 4502 | T |
| Parietal Lobe | 0.3971 | Genome-2 | Genome-2 | C | 4502 | T |
| Upper Brain Stem | 0.163 | Genome-2 | Genome-2 | C | 4502 | T |
| Midbrain | 0.204 | Genome-2 | Genome-2 | C | 4502 | T |
| Frontal Cortex 1 | 0.3304 | Genome-2 | Genome-2 | C | 4502 | T |
| SSPE 2 | 0.2755 | Genome-2 | Genome-2 | C | 4502 | T |
| Brain Stem | 0.0866 | Genome-2 | Genome-2 | C | 4502 | T |
| Occipital Lobe | 0.879 | Genome-2 | Genome-2 | C | 4502 | T |
| SSPE 1 | 0.3178 | Genome-2 | Genome-2 | C | 4502 | T |
| Cerebellum | 0 | Genome-2 | Genome-2 | C | 4502 | T |
| Cerebellum Nucleus | 0.2364 | Genome-2 | Genome-2 | C | 4502 | T |
| Frontal Cortex 2 | 0.4181 | Genome-2 | Genome-2 | C | 4502 | T |
| Internal Capsule | 0.5214 | Genome-2 | Genome-2 | C | 4502 | T |
| Frontal Cortex 3 | 0.3467 | Genome-2 | Genome-2 | C | 4502 | T |
| Hippocampus | 0.7507 | Genome-2 | Genome-2 | C | 4502 | T |
| Temporal Lobe | 0.0767 | Genome-1 | Genome-01 | C | 4573 | T |
| Parietal Lobe | 0.551 | Genome-1 | Genome-01 | C | 4573 | T |
| Upper Brain Stem | 0.7862 | Genome-1 | Genome-01 | C | 4573 | T |
| Midbrain | 0.7464 | Genome-1 | Genome-01 | C | 4573 | T |
| Frontal Cortex 1 | 0.6119 | Genome-1 | Genome-01 | C | 4573 | T |
| SSPE 2 | 0.6919 | Genome-1 | Genome-01 | C | 4573 | T |
| Brain Stem | 0.8638 | Genome-1 | Genome-01 | C | 4573 | T |
| Occipital Lobe | 0.1006 | Genome-1 | Genome-01 | C | 4573 | T |
| SSPE 1 | 0.6535 | Genome-1 | Genome-01 | C | 4573 | T |
| Cerebellum | 0.795455 | Genome-1 | Genome-01 | C | 4573 | T |
| Cerebellum Nucleus | 0.7375 | Genome-1 | Genome-01 | C | 4573 | T |
| Frontal Cortex 2 | 0.5533 | Genome-1 | Genome-01 | C | 4573 | T |
| Internal Capsule | 0.453 | Genome-1 | Genome-01 | C | 4573 | T |
| Frontal Cortex 3 | 0.5989 | Genome-1 | Genome-01 | C | 4573 | T |
| Hippocampus | 0.1931 | Genome-1 | Genome-01 | C | 4573 | T |
| Temporal Lobe | 0.1033 | Genome-2 | cluster 11 | C | 4803 | A |
| Parietal Lobe | 0.0832 | Genome-2 | cluster 11 | C | 4803 | A |
| Upper Brain Stem | 0.0772 | Genome-2 | cluster 11 | C | 4803 | A |
| Midbrain | 0 | Genome-2 | cluster 11 | C | 4803 | A |
| Frontal Cortex 1 | 0 | Genome-2 | cluster 11 | C | 4803 | A |
| SSPE 2 | 0 | Genome-2 | cluster 11 | C | 4803 | A |
| Brain Stem | 0 | Genome-2 | cluster 11 | C | 4803 | A |
| Occipital Lobe | 0 | Genome-2 | cluster 11 | C | 4803 | A |
| SSPE 1 | 0 | Genome-2 | cluster 11 | C | 4803 | A |
| Cerebellum | 0 | Genome-2 | cluster 11 | C | 4803 | A |
| Cerebellum Nucleus | 0 | Genome-2 | cluster 11 | C | 4803 | A |
| Frontal Cortex 2 | 0 | Genome-2 | cluster 11 | C | 4803 | A |
| Internal Capsule | 0.0318 | Genome-2 | cluster 11 | C | 4803 | A |
| Frontal Cortex 3 | 0 | Genome-2 | cluster 11 | C | 4803 | A |
| Hippocampus | 0.0554 | Genome-2 | cluster 11 | C | 4803 | A |
| Temporal Lobe | 0.8959 | Genome-2 | Genome-2 | C | 5742 | T |
| Parietal Lobe | 0.3248 | Genome-2 | Genome-2 | C | 5742 | T |
| Upper Brain Stem | 0.1881 | Genome-2 | Genome-2 | C | 5742 | T |
| Midbrain | 0.2354 | Genome-2 | Genome-2 | C | 5742 | T |
| Frontal Cortex 1 | 0.3322 | Genome-2 | Genome-2 | C | 5742 | T |
| SSPE 2 | 0.2459 | Genome-2 | Genome-2 | C | 5742 | T |
| Brain Stem | 0.0744 | Genome-2 | Genome-2 | C | 5742 | T |
| Occipital Lobe | 0.8744 | Genome-2 | Genome-2 | C | 5742 | T |
| SSPE 1 | 0.295 | Genome-2 | Genome-2 | C | 5742 | T |
| Cerebellum | 0.0946 | Genome-2 | Genome-2 | C | 5742 | T |
| Cerebellum Nucleus | 0.3125 | Genome-2 | Genome-2 | C | 5742 | T |
| Frontal Cortex 2 | 0.5423 | Genome-2 | Genome-2 | C | 5742 | T |
| Internal Capsule | 0.5401 | Genome-2 | Genome-2 | C | 5742 | T |
| Frontal Cortex 3 | 0.334 | Genome-2 | Genome-2 | C | 5742 | T |
| Hippocampus | 0.7704 | Genome-2 | Genome-2 | C | 5742 | T |
| Temporal Lobe | 0.106 | Genome-2 | cluster 11 | C | 5997 | T |
| Parietal Lobe | 0.0624 | Genome-2 | cluster 11 | C | 5997 | T |
| Upper Brain Stem | 0.1114 | Genome-2 | cluster 11 | C | 5997 | T |
| Midbrain | 0 | Genome-2 | cluster 11 | C | 5997 | T |
| Frontal Cortex 1 | 0 | Genome-2 | cluster 11 | C | 5997 | T |
| SSPE 2 | 0 | Genome-2 | cluster 11 | C | 5997 | T |
| Brain Stem | 0 | Genome-2 | cluster 11 | C | 5997 | T |
| Occipital Lobe | 0 | Genome-2 | cluster 11 | C | 5997 | T |
| SSPE 1 | 0 | Genome-2 | cluster 11 | C | 5997 | T |
| Cerebellum | 0 | Genome-2 | cluster 11 | C | 5997 | T |
| Cerebellum Nucleus | 0 | Genome-2 | cluster 11 | C | 5997 | T |
| Frontal Cortex 2 | 0 | Genome-2 | cluster 11 | C | 5997 | T |
| Internal Capsule | 0.03 | Genome-2 | cluster 11 | C | 5997 | T |
| Frontal Cortex 3 | 0 | Genome-2 | cluster 11 | C | 5997 | T |
| Hippocampus | 0.0458 | Genome-2 | cluster 11 | C | 5997 | T |
| Temporal Lobe | 0.0748 | Genome-1 | Genome-1 | G | 6336 | A |
| Parietal Lobe | 0.6355 | Genome-1 | Genome-1 | G | 6336 | A |
| Upper Brain Stem | 0.7641 | Genome-1 | Genome-1 | G | 6336 | A |
| Midbrain | 0.7188 | Genome-1 | Genome-1 | G | 6336 | A |
| Frontal Cortex 1 | 0.6105 | Genome-1 | Genome-1 | G | 6336 | A |
| SSPE 2 | 0.7332 | Genome-1 | Genome-1 | G | 6336 | A |
| Brain Stem | 0.8934 | Genome-1 | Genome-1 | G | 6336 | A |
| Occipital Lobe | 0.0543 | Genome-1 | Genome-1 | G | 6336 | A |
| SSPE 1 | 0.6667 | Genome-1 | Genome-1 | G | 6336 | A |
| Cerebellum | 0.9004 | Genome-1 | Genome-1 | G | 6336 | A |
| Cerebellum Nucleus | 0.6363 | Genome-1 | Genome-1 | G | 6336 | A |
| Frontal Cortex 2 | 0.02 | Genome-1 | Genome-1 | G | 6336 | A |
| Internal Capsule | 0.4036 | Genome-1 | Genome-1 | G | 6336 | A |
| Frontal Cortex 3 | 0.6141 | Genome-1 | Genome-1 | G | 6336 | A |
| Hippocampus | 0.1907 | Genome-1 | Genome-1 | G | 6336 | A |
| Temporal Lobe | 0.103 | Genome-2 | cluster 11 | G | 6828 | A |
| Parietal Lobe | 0.0647 | Genome-2 | cluster 11 | G | 6828 | A |
| Upper Brain Stem | 0.1079 | Genome-2 | cluster 11 | G | 6828 | A |
| Midbrain | 0 | Genome-2 | cluster 11 | G | 6828 | A |
| Frontal Cortex 1 | 0 | Genome-2 | cluster 11 | G | 6828 | A |
| SSPE 2 | 0 | Genome-2 | cluster 11 | G | 6828 | A |
| Brain Stem | 0 | Genome-2 | cluster 11 | G | 6828 | A |
| Occipital Lobe | 0 | Genome-2 | cluster 11 | G | 6828 | A |
| SSPE 1 | 0 | Genome-2 | cluster 11 | G | 6828 | A |
| Cerebellum | 0 | Genome-2 | cluster 11 | G | 6828 | A |
| Cerebellum Nucleus | 0 | Genome-2 | cluster 11 | G | 6828 | A |
| Frontal Cortex 2 | 0 | Genome-2 | cluster 11 | G | 6828 | A |
| Internal Capsule | 0.0282 | Genome-2 | cluster 11 | G | 6828 | A |
| Frontal Cortex 3 | 0 | Genome-2 | cluster 11 | G | 6828 | A |
| Hippocampus | 0.0495 | Genome-2 | cluster 11 | G | 6828 | A |
| Temporal Lobe | 0.9216 | Genome-2 | Genome-2 | T | 7333 | C |
| Parietal Lobe | 0.3519 | Genome-2 | Genome-2 | T | 7333 | C |
| Upper Brain Stem | 0.2298 | Genome-2 | Genome-2 | T | 7333 | C |
| Midbrain | 0.2828 | Genome-2 | Genome-2 | T | 7333 | C |
| Frontal Cortex 1 | 0.3668 | Genome-2 | Genome-2 | T | 7333 | C |
| SSPE 2 | 0.2546 | Genome-2 | Genome-2 | T | 7333 | C |
| Brain Stem | 0.0855 | Genome-2 | Genome-2 | T | 7333 | C |
| Occipital Lobe | 0.9172 | Genome-2 | Genome-2 | T | 7333 | C |
| SSPE 1 | 0.3216 | Genome-2 | Genome-2 | T | 7333 | C |
| Cerebellum | 0.0954 | Genome-2 | Genome-2 | T | 7333 | C |
| Cerebellum Nucleus | 0.3934 | Genome-2 | Genome-2 | T | 7333 | C |
| Frontal Cortex 2 | 0.478 | Genome-2 | Genome-2 | T | 7333 | C |
| Internal Capsule | 0.5888 | Genome-2 | Genome-2 | T | 7333 | C |
| Frontal Cortex 3 | 0.3714 | Genome-2 | Genome-2 | T | 7333 | C |
| Hippocampus | 0.8063 | Genome-2 | Genome-2 | T | 7333 | C |
| Temporal Lobe | 0.0737 | Genome-1 | Genome-01 | A | 7824 | G |
| Parietal Lobe | 0.6219 | Genome-1 | Genome-01 | A | 7824 | G |
| Upper Brain Stem | 0.7619 | Genome-1 | Genome-01 | A | 7824 | G |
| Midbrain | 0.7015 | Genome-1 | Genome-01 | A | 7824 | G |
| Frontal Cortex 1 | 0.6121 | Genome-1 | Genome-01 | A | 7824 | G |
| SSPE 2 | 0.7226 | Genome-1 | Genome-01 | A | 7824 | G |
| Brain Stem | 0.9052 | Genome-1 | Genome-01 | A | 7824 | G |
| Occipital Lobe | 0.0764 | Genome-1 | Genome-01 | A | 7824 | G |
| SSPE 1 | 0.6578 | Genome-1 | Genome-01 | A | 7824 | G |
| Cerebellum | 0.8793 | Genome-1 | Genome-01 | A | 7824 | G |
| Cerebellum Nucleus | 0.6153 | Genome-1 | Genome-01 | A | 7824 | G |
| Frontal Cortex 2 | 0.4767 | Genome-1 | Genome-01 | A | 7824 | G |
| Internal Capsule | 0.3986 | Genome-1 | Genome-01 | A | 7824 | G |
| Frontal Cortex 3 | 0.6024 | Genome-1 | Genome-01 | A | 7824 | G |
| Hippocampus | 0.1751 | Genome-1 | Genome-01 | A | 7824 | G |
| Temporal Lobe | 0.4481 | Genome-2 | cluster 9 | G | 8059 | A |
| Parietal Lobe | 0 | Genome-2 | cluster 9 | G | 8059 | A |
| Upper Brain Stem | 0.0599 | Genome-2 | cluster 9 | G | 8059 | A |
| Midbrain | 0.0256 | Genome-2 | cluster 9 | G | 8059 | A |
| Frontal Cortex 1 | 0 | Genome-2 | cluster 9 | G | 8059 | A |
| SSPE 2 | 0 | Genome-2 | cluster 9 | G | 8059 | A |
| Brain Stem | 0 | Genome-2 | cluster 9 | G | 8059 | A |
| Occipital Lobe | 0.2377 | Genome-2 | cluster 9 | G | 8059 | A |
| SSPE 1 | 0 | Genome-2 | cluster 9 | G | 8059 | A |
| Cerebellum | 0 | Genome-2 | cluster 9 | G | 8059 | A |
| Cerebellum Nucleus | 0 | Genome-2 | cluster 9 | G | 8059 | A |
| Frontal Cortex 2 | 0 | Genome-2 | cluster 9 | G | 8059 | A |
| Internal Capsule | 0.0211 | Genome-2 | cluster 9 | G | 8059 | A |
| Frontal Cortex 3 | 0 | Genome-2 | cluster 9 | G | 8059 | A |
| Hippocampus | 0 | Genome-2 | cluster 9 | G | 8059 | A |
| Temporal Lobe | 0.9231 | Genome-2 | Genome-2 | T | 8428 | C |
| Parietal Lobe | 0.3579 | Genome-2 | Genome-2 | T | 8428 | C |
| Upper Brain Stem | 0.2094 | Genome-2 | Genome-2 | T | 8428 | C |
| Midbrain | 0.2667 | Genome-2 | Genome-2 | T | 8428 | C |
| Frontal Cortex 1 | 0.3823 | Genome-2 | Genome-2 | T | 8428 | C |
| SSPE 2 | 0.2556 | Genome-2 | Genome-2 | T | 8428 | C |
| Brain Stem | 0.1067 | Genome-2 | Genome-2 | T | 8428 | C |
| Occipital Lobe | 0.8968 | Genome-2 | Genome-2 | T | 8428 | C |
| SSPE 1 | 0.3107 | Genome-2 | Genome-2 | T | 8428 | C |
| Cerebellum | 0.0799 | Genome-2 | Genome-2 | T | 8428 | C |
| Cerebellum Nucleus | 0.3381 | Genome-2 | Genome-2 | T | 8428 | C |
| Frontal Cortex 2 | 0.5028 | Genome-2 | Genome-2 | T | 8428 | C |
| Internal Capsule | 0.5614 | Genome-2 | Genome-2 | T | 8428 | C |
| Frontal Cortex 3 | 0.3639 | Genome-2 | Genome-2 | T | 8428 | C |
| Hippocampus | 0.7874 | Genome-2 | Genome-2 | T | 8428 | C |
| Temporal Lobe | 0.9212 | Genome-2 | Genome-2 | T | 8434 | C |
| Parietal Lobe | 0.3589 | Genome-2 | Genome-2 | T | 8434 | C |
| Upper Brain Stem | 0.2091 | Genome-2 | Genome-2 | T | 8434 | C |
| Midbrain | 0.2662 | Genome-2 | Genome-2 | T | 8434 | C |
| Frontal Cortex 1 | 0.3819 | Genome-2 | Genome-2 | T | 8434 | C |
| SSPE 2 | 0.2585 | Genome-2 | Genome-2 | T | 8434 | C |
| Brain Stem | 0.1062 | Genome-2 | Genome-2 | T | 8434 | C |
| Occipital Lobe | 0.8954 | Genome-2 | Genome-2 | T | 8434 | C |
| SSPE 1 | 0.3148 | Genome-2 | Genome-2 | T | 8434 | C |
| Cerebellum | 0.0875 | Genome-2 | Genome-2 | T | 8434 | C |
| Cerebellum Nucleus | 0.3371 | Genome-2 | Genome-2 | T | 8434 | C |
| Frontal Cortex 2 | 0.5011 | Genome-2 | Genome-2 | T | 8434 | C |
| Internal Capsule | 0.5577 | Genome-2 | Genome-2 | T | 8434 | C |
| Frontal Cortex 3 | 0.3629 | Genome-2 | Genome-2 | T | 8434 | C |
| Hippocampus | 0.7857 | Genome-2 | Genome-2 | T | 8434 | C |
| Temporal Lobe | 0.9135 | Genome-2 | Genome-2 | T | 8507 | C |
| Parietal Lobe | 0.3356 | Genome-2 | Genome-2 | T | 8507 | C |
| Upper Brain Stem | 0.2024 | Genome-2 | Genome-2 | T | 8507 | C |
| Midbrain | 0.2524 | Genome-2 | Genome-2 | T | 8507 | C |
| Frontal Cortex 1 | 0.3461 | Genome-2 | Genome-2 | T | 8507 | C |
| SSPE 2 | 0.2333 | Genome-2 | Genome-2 | T | 8507 | C |
| Brain Stem | 0.0837 | Genome-2 | Genome-2 | T | 8507 | C |
| Occipital Lobe | 0.883 | Genome-2 | Genome-2 | T | 8507 | C |
| SSPE 1 | 0.3343 | Genome-2 | Genome-2 | T | 8507 | C |
| Cerebellum | 0.0816 | Genome-2 | Genome-2 | T | 8507 | C |
| Cerebellum Nucleus | 0.3124 | Genome-2 | Genome-2 | T | 8507 | C |
| Frontal Cortex 2 | 0.4746 | Genome-2 | Genome-2 | T | 8507 | C |
| Internal Capsule | 0.5338 | Genome-2 | Genome-2 | T | 8507 | C |
| Frontal Cortex 3 | 0.3399 | Genome-2 | Genome-2 | T | 8507 | C |
| Hippocampus | 0.765 | Genome-2 | Genome-2 | T | 8507 | C |
| Temporal Lobe | 0.918 | Genome-2 | Genome-2 | T | 8539 | C |
| Parietal Lobe | 0.361 | Genome-2 | Genome-2 | T | 8539 | C |
| Upper Brain Stem | 0.222 | Genome-2 | Genome-2 | T | 8539 | C |
| Midbrain | 0.2729 | Genome-2 | Genome-2 | T | 8539 | C |
| Frontal Cortex 1 | 0.3602 | Genome-2 | Genome-2 | T | 8539 | C |
| SSPE 2 | 0.2584 | Genome-2 | Genome-2 | T | 8539 | C |
| Brain Stem | 0.092 | Genome-2 | Genome-2 | T | 8539 | C |
| Occipital Lobe | 0.8861 | Genome-2 | Genome-2 | T | 8539 | C |
| SSPE 1 | 0.3548 | Genome-2 | Genome-2 | T | 8539 | C |
| Cerebellum | 0.0867 | Genome-2 | Genome-2 | T | 8539 | C |
| Cerebellum Nucleus | 0.3499 | Genome-2 | Genome-2 | T | 8539 | C |
| Frontal Cortex 2 | 0.4998 | Genome-2 | Genome-2 | T | 8539 | C |
| Internal Capsule | 0.5672 | Genome-2 | Genome-2 | T | 8539 | C |
| Frontal Cortex 3 | 0.3777 | Genome-2 | Genome-2 | T | 8539 | C |
| Hippocampus | 0.796 | Genome-2 | Genome-2 | T | 8539 | C |
| Temporal Lobe | 0.4517 | Genome-2 | cluster 9 | C | 8714 | T |
| Parietal Lobe | 0 | Genome-2 | cluster 9 | C | 8714 | T |
| Upper Brain Stem | 0.058 | Genome-2 | cluster 9 | C | 8714 | T |
| Midbrain | 0.0264 | Genome-2 | cluster 9 | C | 8714 | T |
| Frontal Cortex 1 | 0 | Genome-2 | cluster 9 | C | 8714 | T |
| SSPE 2 | 0 | Genome-2 | cluster 9 | C | 8714 | T |
| Brain Stem | 0 | Genome-2 | cluster 9 | C | 8714 | T |
| Occipital Lobe | 0.2391 | Genome-2 | cluster 9 | C | 8714 | T |
| SSPE 1 | 0 | Genome-2 | cluster 9 | C | 8714 | T |
| Cerebellum | 0 | Genome-2 | cluster 9 | C | 8714 | T |
| Cerebellum Nucleus | 0 | Genome-2 | cluster 9 | C | 8714 | T |
| Frontal Cortex 2 | 0 | Genome-2 | cluster 9 | C | 8714 | T |
| Internal Capsule | 0.0232 | Genome-2 | cluster 9 | C | 8714 | T |
| Frontal Cortex 3 | 0 | Genome-2 | cluster 9 | C | 8714 | T |
| Hippocampus | 0 | Genome-2 | cluster 9 | C | 8714 | T |
| Temporal Lobe | 0.9228 | Genome-2 | Genome-2 | T | 8920 | C |
| Parietal Lobe | 0.373 | Genome-2 | Genome-2 | T | 8920 | C |
| Upper Brain Stem | 0.2435 | Genome-2 | Genome-2 | T | 8920 | C |
| Midbrain | 0.2894 | Genome-2 | Genome-2 | T | 8920 | C |
| Frontal Cortex 1 | 0.3819 | Genome-2 | Genome-2 | T | 8920 | C |
| SSPE 2 | 0.255 | Genome-2 | Genome-2 | T | 8920 | C |
| Brain Stem | 0.0968 | Genome-2 | Genome-2 | T | 8920 | C |
| Occipital Lobe | 0.8957 | Genome-2 | Genome-2 | T | 8920 | C |
| SSPE 1 | 0.3464 | Genome-2 | Genome-2 | T | 8920 | C |
| Cerebellum | 0.0985 | Genome-2 | Genome-2 | T | 8920 | C |
| Cerebellum Nucleus | 0.3744 | Genome-2 | Genome-2 | T | 8920 | C |
| Frontal Cortex 2 | 0.5111 | Genome-2 | Genome-2 | T | 8920 | C |
| Internal Capsule | 0.5936 | Genome-2 | Genome-2 | T | 8920 | C |
| Frontal Cortex 3 | 0.395 | Genome-2 | Genome-2 | T | 8920 | C |
| Hippocampus | 0.8136 | Genome-2 | Genome-2 | T | 8920 | C |
| Temporal Lobe | 0.0743 | Genome-1 | Genome-01 | C | 9243 | T |
| Parietal Lobe | 0.6265 | Genome-1 | Genome-01 | C | 9243 | T |
| Upper Brain Stem | 0.7503 | Genome-1 | Genome-01 | C | 9243 | T |
| Midbrain | 0.7037 | Genome-1 | Genome-01 | C | 9243 | T |
| Frontal Cortex 1 | 0.6078 | Genome-1 | Genome-01 | C | 9243 | T |
| SSPE 2 | 0.717 | Genome-1 | Genome-01 | C | 9243 | T |
| Brain Stem | 0.9053 | Genome-1 | Genome-01 | C | 9243 | T |
| Occipital Lobe | 0.0778 | Genome-1 | Genome-01 | C | 9243 | T |
| SSPE 1 | 0.6521 | Genome-1 | Genome-01 | C | 9243 | T |
| Cerebellum | 0.8609 | Genome-1 | Genome-01 | C | 9243 | T |
| Cerebellum Nucleus | 0.6202 | Genome-1 | Genome-01 | C | 9243 | T |
| Frontal Cortex 2 | 0.465 | Genome-1 | Genome-01 | C | 9243 | T |
| Internal Capsule | 0.3879 | Genome-1 | Genome-01 | C | 9243 | T |
| Frontal Cortex 3 | 0.6 | Genome-1 | Genome-01 | C | 9243 | T |
| Hippocampus | 0.1736 | Genome-1 | Genome-01 | C | 9243 | T |
| Temporal Lobe | 0.9174 | Genome-2 | Genome-2 | C | 9848 | T |
| Parietal Lobe | 0.3232 | Genome-2 | Genome-2 | C | 9848 | T |
| Upper Brain Stem | 0.2206 | Genome-2 | Genome-2 | C | 9848 | T |
| Midbrain | 0.2724 | Genome-2 | Genome-2 | C | 9848 | T |
| Frontal Cortex 1 | 0.3635 | Genome-2 | Genome-2 | C | 9848 | T |
| SSPE 2 | 0.2632 | Genome-2 | Genome-2 | C | 9848 | T |
| Brain Stem | 0.0892 | Genome-2 | Genome-2 | C | 9848 | T |
| Occipital Lobe | 0.8868 | Genome-2 | Genome-2 | C | 9848 | T |
| SSPE 1 | 0.3237 | Genome-2 | Genome-2 | C | 9848 | T |
| Cerebellum | 0.0838 | Genome-2 | Genome-2 | C | 9848 | T |
| Cerebellum Nucleus | 0.3606 | Genome-2 | Genome-2 | C | 9848 | T |
| Frontal Cortex 2 | 0.454 | Genome-2 | Genome-2 | C | 9848 | T |
| Internal Capsule | 0.5705 | Genome-2 | Genome-2 | C | 9848 | T |
| Frontal Cortex 3 | 0.3586 | Genome-2 | Genome-2 | C | 9848 | T |
| Hippocampus | 0.7918 | Genome-2 | Genome-2 | C | 9848 | T |
| Temporal Lobe | 0.2627 | Genome-2 | cluster 7 | A | 10514 | G |
| Parietal Lobe | 0.1002 | Genome-2 | cluster 7 | A | 10514 | G |
| Upper Brain Stem | 0 | Genome-2 | cluster 7 | A | 10514 | G |
| Midbrain | 0 | Genome-2 | cluster 7 | A | 10514 | G |
| Frontal Cortex 1 | 0 | Genome-2 | cluster 7 | A | 10514 | G |
| SSPE 2 | 0 | Genome-2 | cluster 7 | A | 10514 | G |
| Brain Stem | 0 | Genome-2 | cluster 7 | A | 10514 | G |
| Occipital Lobe | 0.559 | Genome-2 | cluster 7 | A | 10514 | G |
| SSPE 1 | 0 | Genome-2 | cluster 7 | A | 10514 | G |
| Cerebellum | 0 | Genome-2 | cluster 7 | A | 10514 | G |
| Cerebellum Nucleus | 0.0575 | Genome-2 | cluster 7 | A | 10514 | G |
| Frontal Cortex 2 | 0.4927 | Genome-2 | cluster 7 | A | 10514 | G |
| Internal Capsule | 0.234 | Genome-2 | cluster 7 | A | 10514 | G |
| Frontal Cortex 3 | 0 | Genome-2 | cluster 7 | A | 10514 | G |
| Hippocampus | 0.4564 | Genome-2 | cluster 7 | A | 10514 | G |
| Temporal Lobe | 0.9135 | Genome-2 | Genome-2 | A | 10718 | G |
| Parietal Lobe | 0.3471 | Genome-2 | Genome-2 | A | 10718 | G |
| Upper Brain Stem | 0.2276 | Genome-2 | Genome-2 | A | 10718 | G |
| Midbrain | 0.2709 | Genome-2 | Genome-2 | A | 10718 | G |
| Frontal Cortex 1 | 0.3634 | Genome-2 | Genome-2 | A | 10718 | G |
| SSPE 2 | 0.2742 | Genome-2 | Genome-2 | A | 10718 | G |
| Brain Stem | 0.0837 | Genome-2 | Genome-2 | A | 10718 | G |
| Occipital Lobe | 0.8833 | Genome-2 | Genome-2 | A | 10718 | G |
| SSPE 1 | 0.3369 | Genome-2 | Genome-2 | A | 10718 | G |
| Cerebellum | 0.1079 | Genome-2 | Genome-2 | A | 10718 | G |
| Cerebellum Nucleus | 0.3806 | Genome-2 | Genome-2 | A | 10718 | G |
| Frontal Cortex 2 | 0.4795 | Genome-2 | Genome-2 | A | 10718 | G |
| Internal Capsule | 0.5815 | Genome-2 | Genome-2 | A | 10718 | G |
| Frontal Cortex 3 | 0.3711 | Genome-2 | Genome-2 | A | 10718 | G |
| Hippocampus | 0.804 | Genome-2 | Genome-2 | A | 10718 | G |
| Temporal Lobe | 0.4056 | Genome-2 | cluster 9 | G | 10785 | A |
| Parietal Lobe | 0 | Genome-2 | cluster 9 | G | 10785 | A |
| Upper Brain Stem | 0.0473 | Genome-2 | cluster 9 | G | 10785 | A |
| Midbrain | 0.021885 | Genome-2 | cluster 9 | G | 10785 | A |
| Frontal Cortex 1 | 0 | Genome-2 | cluster 9 | G | 10785 | A |
| SSPE 2 | 0 | Genome-2 | cluster 9 | G | 10785 | A |
| Brain Stem | 0 | Genome-2 | cluster 9 | G | 10785 | A |
| Occipital Lobe | 0.2085 | Genome-2 | cluster 9 | G | 10785 | A |
| SSPE 1 | 0 | Genome-2 | cluster 9 | G | 10785 | A |
| Cerebellum | 0 | Genome-2 | cluster 9 | G | 10785 | A |
| Cerebellum Nucleus | 0 | Genome-2 | cluster 9 | G | 10785 | A |
| Frontal Cortex 2 | 0 | Genome-2 | cluster 9 | G | 10785 | A |
| Internal Capsule | 0.020994 | Genome-2 | cluster 9 | G | 10785 | A |
| Frontal Cortex 3 | 0 | Genome-2 | cluster 9 | G | 10785 | A |
| Hippocampus | 0 | Genome-2 | cluster 9 | G | 10785 | A |
| Temporal Lobe | 0.0809 | Genome-1 | Genome-01 | A | 11249 | G |
| Parietal Lobe | 0.6529 | Genome-1 | Genome-01 | A | 11249 | G |
| Upper Brain Stem | 0.7565 | Genome-1 | Genome-01 | A | 11249 | G |
| Midbrain | 0.716 | Genome-1 | Genome-01 | A | 11249 | G |
| Frontal Cortex 1 | 0.6233 | Genome-1 | Genome-01 | A | 11249 | G |
| SSPE 2 | 0.7151 | Genome-1 | Genome-01 | A | 11249 | G |
| Brain Stem | 0.9109 | Genome-1 | Genome-01 | A | 11249 | G |
| Occipital Lobe | 0.0864 | Genome-1 | Genome-01 | A | 11249 | G |
| SSPE 1 | 0.661 | Genome-1 | Genome-01 | A | 11249 | G |
| Cerebellum | 0.9119 | Genome-1 | Genome-01 | A | 11249 | G |
| Cerebellum Nucleus | 0.624 | Genome-1 | Genome-01 | A | 11249 | G |
| Frontal Cortex 2 | 0.5038 | Genome-1 | Genome-01 | A | 11249 | G |
| Internal Capsule | 0.3978 | Genome-1 | Genome-01 | A | 11249 | G |
| Frontal Cortex 3 | 0.6079 | Genome-1 | Genome-01 | A | 11249 | G |
| Hippocampus | 0.1864 | Genome-1 | Genome-01 | A | 11249 | G |
| Temporal Lobe | 0.0735 | Genome-1 | Genome-1 | T | 12137 | C |
| Parietal Lobe | 0.6194 | Genome-1 | Genome-1 | T | 12137 | C |
| Upper Brain Stem | 0.7469 | Genome-1 | Genome-1 | T | 12137 | C |
| Midbrain | 0.7111 | Genome-1 | Genome-1 | T | 12137 | C |
| Frontal Cortex 1 | 0.6116 | Genome-1 | Genome-1 | T | 12137 | C |
| SSPE 2 | 0.7083 | Genome-1 | Genome-1 | T | 12137 | C |
| Brain Stem | 0.9031 | Genome-1 | Genome-1 | T | 12137 | C |
| Occipital Lobe | 0.0622 | Genome-1 | Genome-1 | T | 12137 | C |
| SSPE 1 | 0.642 | Genome-1 | Genome-1 | T | 12137 | C |
| Cerebellum | 0.8876 | Genome-1 | Genome-1 | T | 12137 | C |
| Cerebellum Nucleus | 0.5849 | Genome-1 | Genome-1 | T | 12137 | C |
| Frontal Cortex 2 | 0.02051 | Genome-1 | Genome-1 | T | 12137 | C |
| Internal Capsule | 0.4001 | Genome-1 | Genome-1 | T | 12137 | C |
| Frontal Cortex 3 | 0.6059 | Genome-1 | Genome-1 | T | 12137 | C |
| Hippocampus | 0.1798 | Genome-1 | Genome-1 | T | 12137 | C |
| Temporal Lobe | 0.917 | Genome-2 | Genome-2 | A | 12391 | G |
| Parietal Lobe | 0.3435 | Genome-2 | Genome-2 | A | 12391 | G |
| Upper Brain Stem | 0.2346 | Genome-2 | Genome-2 | A | 12391 | G |
| Midbrain | 0.2625 | Genome-2 | Genome-2 | A | 12391 | G |
| Frontal Cortex 1 | 0.3675 | Genome-2 | Genome-2 | A | 12391 | G |
| SSPE 2 | 0.2676 | Genome-2 | Genome-2 | A | 12391 | G |
| Brain Stem | 0.0895 | Genome-2 | Genome-2 | A | 12391 | G |
| Occipital Lobe | 0.8806 | Genome-2 | Genome-2 | A | 12391 | G |
| SSPE 1 | 0.3583 | Genome-2 | Genome-2 | A | 12391 | G |
| Cerebellum | 0.1009 | Genome-2 | Genome-2 | A | 12391 | G |
| Cerebellum Nucleus | 0.3656 | Genome-2 | Genome-2 | A | 12391 | G |
| Frontal Cortex 2 | 0.4851 | Genome-2 | Genome-2 | A | 12391 | G |
| Internal Capsule | 0.578 | Genome-2 | Genome-2 | A | 12391 | G |
| Frontal Cortex 3 | 0.3704 | Genome-2 | Genome-2 | A | 12391 | G |
| Hippocampus | 0.8 | Genome-2 | Genome-2 | A | 12391 | G |
| Temporal Lobe | 0.9049 | Genome-2 | Genome-2 | G | 13208 | A |
| Parietal Lobe | 0.3321 | Genome-2 | Genome-2 | G | 13208 | A |
| Upper Brain Stem | 0.222 | Genome-2 | Genome-2 | G | 13208 | A |
| Midbrain | 0.2608 | Genome-2 | Genome-2 | G | 13208 | A |
| Frontal Cortex 1 | 0.3598 | Genome-2 | Genome-2 | G | 13208 | A |
| SSPE 2 | 0.2573 | Genome-2 | Genome-2 | G | 13208 | A |
| Brain Stem | 0.0852 | Genome-2 | Genome-2 | G | 13208 | A |
| Occipital Lobe | 0.8736 | Genome-2 | Genome-2 | G | 13208 | A |
| SSPE 1 | 0.3268 | Genome-2 | Genome-2 | G | 13208 | A |
| Cerebellum | 0.1012 | Genome-2 | Genome-2 | G | 13208 | A |
| Cerebellum Nucleus | 0.3324 | Genome-2 | Genome-2 | G | 13208 | A |
| Frontal Cortex 2 | 0.4719 | Genome-2 | Genome-2 | G | 13208 | A |
| Internal Capsule | 0.5639 | Genome-2 | Genome-2 | G | 13208 | A |
| Frontal Cortex 3 | 0.359 | Genome-2 | Genome-2 | G | 13208 | A |
| Hippocampus | 0.7974 | Genome-2 | Genome-2 | G | 13208 | A |
| Temporal Lobe | 0.9047 | Genome-2 | Genome-2 | T | 13558 | A |
| Parietal Lobe | 0.3517 | Genome-2 | Genome-2 | T | 13558 | A |
| Upper Brain Stem | 0.252 | Genome-2 | Genome-2 | T | 13558 | A |
| Midbrain | 0.2873 | Genome-2 | Genome-2 | T | 13558 | A |
| Frontal Cortex 1 | 0.3667 | Genome-2 | Genome-2 | T | 13558 | A |
| SSPE 2 | 0.2737 | Genome-2 | Genome-2 | T | 13558 | A |
| Brain Stem | 0.0906 | Genome-2 | Genome-2 | T | 13558 | A |
| Occipital Lobe | 0.8776 | Genome-2 | Genome-2 | T | 13558 | A |
| SSPE 1 | 0.3617 | Genome-2 | Genome-2 | T | 13558 | A |
| Cerebellum | 0.1338 | Genome-2 | Genome-2 | T | 13558 | A |
| Cerebellum Nucleus | 0.3782 | Genome-2 | Genome-2 | T | 13558 | A |
| Frontal Cortex 2 | 0.5155 | Genome-2 | Genome-2 | T | 13558 | A |
| Internal Capsule | 0.5971 | Genome-2 | Genome-2 | T | 13558 | A |
| Frontal Cortex 3 | 0.3958 | Genome-2 | Genome-2 | T | 13558 | A |
| Hippocampus | 0.8148 | Genome-2 | Genome-2 | T | 13558 | A |
| Temporal Lobe | 0.3696 | Genome-2 | cluster 9 | C | 14513 | T |
| Parietal Lobe | 0 | Genome-2 | cluster 9 | C | 14513 | T |
| Upper Brain Stem | 0.0551 | Genome-2 | cluster 9 | C | 14513 | T |
| Midbrain | 0.0237 | Genome-2 | cluster 9 | C | 14513 | T |
| Frontal Cortex 1 | 0 | Genome-2 | cluster 9 | C | 14513 | T |
| SSPE 2 | 0 | Genome-2 | cluster 9 | C | 14513 | T |
| Brain Stem | 0 | Genome-2 | cluster 9 | C | 14513 | T |
| Occipital Lobe | 0.2063 | Genome-2 | cluster 9 | C | 14513 | T |
| SSPE 1 | 0 | Genome-2 | cluster 9 | C | 14513 | T |
| Cerebellum | 0 | Genome-2 | cluster 9 | C | 14513 | T |
| Cerebellum Nucleus | 0 | Genome-2 | cluster 9 | C | 14513 | T |
| Frontal Cortex 2 | 0 | Genome-2 | cluster 9 | C | 14513 | T |
| Internal Capsule | 0.021239 | Genome-2 | cluster 9 | C | 14513 | T |
| Frontal Cortex 3 | 0 | Genome-2 | cluster 9 | C | 14513 | T |
| Hippocampus | 0 | Genome-2 | cluster 9 | C | 14513 | T |
| Temporal Lobe | 0.1138 | Genome-1 | Genome-01 | A | 14650 | G |
| Parietal Lobe | 0.6471 | Genome-1 | Genome-01 | A | 14650 | G |
| Upper Brain Stem | 0.7389 | Genome-1 | Genome-01 | A | 14650 | G |
| Midbrain | 0.7086 | Genome-1 | Genome-01 | A | 14650 | G |
| Frontal Cortex 1 | 0.6054 | Genome-1 | Genome-01 | A | 14650 | G |
| SSPE 2 | 0.6917 | Genome-1 | Genome-01 | A | 14650 | G |
| Brain Stem | 0.9003 | Genome-1 | Genome-01 | A | 14650 | G |
| Occipital Lobe | 0.1053 | Genome-1 | Genome-01 | A | 14650 | G |
| SSPE 1 | 0.6281 | Genome-1 | Genome-01 | A | 14650 | G |
| Cerebellum | 0.8531 | Genome-1 | Genome-01 | A | 14650 | G |
| Cerebellum Nucleus | 0.58 | Genome-1 | Genome-01 | A | 14650 | G |
| Frontal Cortex 2 | 0.4569 | Genome-1 | Genome-01 | A | 14650 | G |
| Internal Capsule | 0.402 | Genome-1 | Genome-01 | A | 14650 | G |
| Frontal Cortex 3 | 0.5955 | Genome-1 | Genome-01 | A | 14650 | G |
| Hippocampus | 0.1826 | Genome-1 | Genome-01 | A | 14650 | G |
| Temporal Lobe | 0.0765 | Genome-1 | Genome-1 | A | 15084 | G |
| Parietal Lobe | 0.6248 | Genome-1 | Genome-1 | A | 15084 | G |
| Upper Brain Stem | 0.6962 | Genome-1 | Genome-1 | A | 15084 | G |
| Midbrain | 0.6751 | Genome-1 | Genome-1 | A | 15084 | G |
| Frontal Cortex 1 | 0.512314 | Genome-1 | Genome-1 | A | 15084 | G |
| SSPE 2 | 0.6635 | Genome-1 | Genome-1 | A | 15084 | G |
| Brain Stem | 0.877669 | Genome-1 | Genome-1 | A | 15084 | G |
| Occipital Lobe | 0.0528 | Genome-1 | Genome-1 | A | 15084 | G |
| SSPE 1 | 0.6082 | Genome-1 | Genome-1 | A | 15084 | G |
| Cerebellum | 0.8278 | Genome-1 | Genome-1 | A | 15084 | G |
| Cerebellum Nucleus | 0.5132 | Genome-1 | Genome-1 | A | 15084 | G |
| Frontal Cortex 2 | 0 | Genome-1 | Genome-1 | A | 15084 | G |
| Internal Capsule | 0.3271 | Genome-1 | Genome-1 | A | 15084 | G |
| Frontal Cortex 3 | 0.4795 | Genome-1 | Genome-1 | A | 15084 | G |
| Hippocampus | 0.1743 | Genome-1 | Genome-1 | A | 15084 | G |
| Temporal Lobe | 0.8943 | Genome-2 | Genome-2 | A | 15086 | G |
| Parietal Lobe | 0.347 | Genome-2 | Genome-2 | A | 15086 | G |
| Upper Brain Stem | 0.2691 | Genome-2 | Genome-2 | A | 15086 | G |
| Midbrain | 0.3094 | Genome-2 | Genome-2 | A | 15086 | G |
| Frontal Cortex 1 | 0.4679 | Genome-2 | Genome-2 | A | 15086 | G |
| SSPE 2 | 0.3151 | Genome-2 | Genome-2 | A | 15086 | G |
| Brain Stem | 0.1173 | Genome-2 | Genome-2 | A | 15086 | G |
| Occipital Lobe | 0.7227 | Genome-2 | Genome-2 | A | 15086 | G |
| SSPE 1 | 0.3704 | Genome-2 | Genome-2 | A | 15086 | G |
| Cerebellum | 0.1401 | Genome-2 | Genome-2 | A | 15086 | G |
| Cerebellum Nucleus | 0.4186 | Genome-2 | Genome-2 | A | 15086 | G |
| Frontal Cortex 2 | 0.5547 | Genome-2 | Genome-2 | A | 15086 | G |
| Internal Capsule | 0.608 | Genome-2 | Genome-2 | A | 15086 | G |
| Frontal Cortex 3 | 0.4866 | Genome-2 | Genome-2 | A | 15086 | G |
| Hippocampus | 0.8053 | Genome-2 | Genome-2 | A | 15086 | G |
| Temporal Lobe | 0.1122 | Genome-1 | Genome-01 | A | 15293 | T |
| Parietal Lobe | 0.6757 | Genome-1 | Genome-01 | A | 15293 | T |
| Upper Brain Stem | 0.7358 | Genome-1 | Genome-01 | A | 15293 | T |
| Midbrain | 0.705 | Genome-1 | Genome-01 | A | 15293 | T |
| Frontal Cortex 1 | 0.5432 | Genome-1 | Genome-01 | A | 15293 | T |
| SSPE 2 | 0.7009 | Genome-1 | Genome-01 | A | 15293 | T |
| Brain Stem | 0.8792 | Genome-1 | Genome-01 | A | 15293 | T |
| Occipital Lobe | 0.2619 | Genome-1 | Genome-01 | A | 15293 | T |
| SSPE 1 | 0.6137 | Genome-1 | Genome-01 | A | 15293 | T |
| Cerebellum | 0.866 | Genome-1 | Genome-01 | A | 15293 | T |
| Cerebellum Nucleus | 0.5676 | Genome-1 | Genome-01 | A | 15293 | T |
| Frontal Cortex 2 | 0.4094 | Genome-1 | Genome-01 | A | 15293 | T |
| Internal Capsule | 0.4042 | Genome-1 | Genome-01 | A | 15293 | T |
| Frontal Cortex 3 | 0.5315 | Genome-1 | Genome-01 | A | 15293 | T |
| Hippocampus | 0.2962 | Genome-1 | Genome-01 | A | 15293 | T |
| Temporal Lobe | 0.8837 | Genome-2 | Genome-2 | T | 15377 | C |
| Parietal Lobe | 0.31 | Genome-2 | Genome-2 | T | 15377 | C |
| Upper Brain Stem | 0.2679 | Genome-2 | Genome-2 | T | 15377 | C |
| Midbrain | 0.2958 | Genome-2 | Genome-2 | T | 15377 | C |
| Frontal Cortex 1 | 0.459 | Genome-2 | Genome-2 | T | 15377 | C |
| SSPE 2 | 0.3365 | Genome-2 | Genome-2 | T | 15377 | C |
| Brain Stem | 0.1321 | Genome-2 | Genome-2 | T | 15377 | C |
| Occipital Lobe | 0.7297 | Genome-2 | Genome-2 | T | 15377 | C |
| SSPE 1 | 0.3942 | Genome-2 | Genome-2 | T | 15377 | C |
| Cerebellum | 0.168 | Genome-2 | Genome-2 | T | 15377 | C |
| Cerebellum Nucleus | 0.44 | Genome-2 | Genome-2 | T | 15377 | C |
| Frontal Cortex 2 | 0.5973 | Genome-2 | Genome-2 | T | 15377 | C |
| Internal Capsule | 0.6085 | Genome-2 | Genome-2 | T | 15377 | C |
| Frontal Cortex 3 | 0.4671 | Genome-2 | Genome-2 | T | 15377 | C |
| Hippocampus | 0.7079 | Genome-2 | Genome-2 | T | 15377 | C |
| Temporal Lobe | 0 | Genome-2 | cluster 8 | G | 1632 | A |
| Parietal Lobe | 0.1605 | Genome-2 | cluster 8 | G | 1632 | A |
| Upper Brain Stem | 0 | Genome-2 | cluster 8 | G | 1632 | A |
| Midbrain | 0 | Genome-2 | cluster 8 | G | 1632 | A |
| Frontal Cortex 1 | 0.3569 | Genome-2 | cluster 8 | G | 1632 | A |
| SSPE 2 | 0.2883 | Genome-2 | cluster 8 | G | 1632 | A |
| Brain Stem | 0 | Genome-2 | cluster 8 | G | 1632 | A |
| Occipital Lobe | 0 | Genome-2 | cluster 8 | G | 1632 | A |
| SSPE 1 | 0.3126 | Genome-2 | cluster 8 | G | 1632 | A |
| Cerebellum | 0.0375 | Genome-2 | cluster 8 | G | 1632 | A |
| Cerebellum Nucleus | 0.0493 | Genome-2 | cluster 8 | G | 1632 | A |
| Frontal Cortex 2 | 0 | Genome-2 | cluster 8 | G | 1632 | A |
| Internal Capsule | 0.0537 | Genome-2 | cluster 8 | G | 1632 | A |
| Frontal Cortex 3 | 0.3752 | Genome-2 | cluster 8 | G | 1632 | A |
| Hippocampus | 0.143 | Genome-2 | cluster 8 | G | 1632 | A |
| Temporal Lobe | 0 | Genome-1 | cluster 3 | G | 1686 | A |
| Parietal Lobe | 0.4023 | Genome-1 | cluster 3 | G | 1686 | A |
| Upper Brain Stem | 0.0278 | Genome-1 | cluster 3 | G | 1686 | A |
| Midbrain | 0 | Genome-1 | cluster 3 | G | 1686 | A |
| Frontal Cortex 1 | 0 | Genome-1 | cluster 3 | G | 1686 | A |
| SSPE 2 | 0 | Genome-1 | cluster 3 | G | 1686 | A |
| Brain Stem | 0 | Genome-1 | cluster 3 | G | 1686 | A |
| Occipital Lobe | 0 | Genome-1 | cluster 3 | G | 1686 | A |
| SSPE 1 | 0 | Genome-1 | cluster 3 | G | 1686 | A |
| Cerebellum | 0 | Genome-1 | cluster 3 | G | 1686 | A |
| Cerebellum Nucleus | 0 | Genome-1 | cluster 3 | G | 1686 | A |
| Frontal Cortex 2 | 0 | Genome-1 | cluster 3 | G | 1686 | A |
| Internal Capsule | 0.0607 | Genome-1 | cluster 3 | G | 1686 | A |
| Frontal Cortex 3 | 0 | Genome-1 | cluster 3 | G | 1686 | A |
| Hippocampus | 0.0388 | Genome-1 | cluster 3 | G | 1686 | A |
| Temporal Lobe | 0 | Genome-1 | cluster 5 | T | 3727 | C |
| Parietal Lobe | 0.0848 | Genome-1 | cluster 5 | T | 3727 | C |
| Upper Brain Stem | 0.0482 | Genome-1 | cluster 5 | T | 3727 | C |
| Midbrain | 0 | Genome-1 | cluster 5 | T | 3727 | C |
| Frontal Cortex 1 | 0 | Genome-1 | cluster 5 | T | 3727 | C |
| SSPE 2 | 0 | Genome-1 | cluster 5 | T | 3727 | C |
| Brain Stem | 0.1376 | Genome-1 | cluster 5 | T | 3727 | C |
| Occipital Lobe | 0.0223 | Genome-1 | cluster 5 | T | 3727 | C |
| SSPE 1 | 0 | Genome-1 | cluster 5 | T | 3727 | C |
| Cerebellum | 0 | Genome-1 | cluster 5 | T | 3727 | C |
| Cerebellum Nucleus | 0.02465 | Genome-1 | cluster 5 | T | 3727 | C |
| Frontal Cortex 2 | 0 | Genome-1 | cluster 5 | T | 3727 | C |
| Internal Capsule | 0.1361 | Genome-1 | cluster 5 | T | 3727 | C |
| Frontal Cortex 3 | 0 | Genome-1 | cluster 5 | T | 3727 | C |
| Hippocampus | 0.0422 | Genome-1 | cluster 5 | T | 3727 | C |
| Temporal Lobe | 0 | Genome-1 | cluster 5 | T | 4178 | C |
| Parietal Lobe | 0.1283 | Genome-1 | cluster 5 | T | 4178 | C |
| Upper Brain Stem | 0.0657 | Genome-1 | cluster 5 | T | 4178 | C |
| Midbrain | 0.0209 | Genome-1 | cluster 5 | T | 4178 | C |
| Frontal Cortex 1 | 0 | Genome-1 | cluster 5 | T | 4178 | C |
| SSPE 2 | 0 | Genome-1 | cluster 5 | T | 4178 | C |
| Brain Stem | 0.1497 | Genome-1 | cluster 5 | T | 4178 | C |
| Occipital Lobe | 0.0247 | Genome-1 | cluster 5 | T | 4178 | C |
| SSPE 1 | 0 | Genome-1 | cluster 5 | T | 4178 | C |
| Cerebellum | 0.0585 | Genome-1 | cluster 5 | T | 4178 | C |
| Cerebellum Nucleus | 0.0475 | Genome-1 | cluster 5 | T | 4178 | C |
| Frontal Cortex 2 | 0.0303 | Genome-1 | cluster 5 | T | 4178 | C |
| Internal Capsule | 0.2241 | Genome-1 | cluster 5 | T | 4178 | C |
| Frontal Cortex 3 | 0 | Genome-1 | cluster 5 | T | 4178 | C |
| Hippocampus | 0.0751 | Genome-1 | cluster 5 | T | 4178 | C |
| Temporal Lobe | 0 | Genome-1 | Genome-FC2 | C | 4761 | T |
| Parietal Lobe | 0.046 | Genome-1 | Genome-FC2 | C | 4761 | T |
| Upper Brain Stem | 0.0403 | Genome-1 | Genome-FC2 | C | 4761 | T |
| Midbrain | 0 | Genome-1 | Genome-FC2 | C | 4761 | T |
| Frontal Cortex 1 | 0 | Genome-1 | Genome-FC2 | C | 4761 | T |
| SSPE 2 | 0 | Genome-1 | Genome-FC2 | C | 4761 | T |
| Brain Stem | 0 | Genome-1 | Genome-FC2 | C | 4761 | T |
| Occipital Lobe | 0 | Genome-1 | Genome-FC2 | C | 4761 | T |
| SSPE 1 | 0 | Genome-1 | Genome-FC2 | C | 4761 | T |
| Cerebellum | 0 | Genome-1 | Genome-FC2 | C | 4761 | T |
| Cerebellum Nucleus | 0.1101 | Genome-1 | Genome-FC2 | C | 4761 | T |
| Frontal Cortex 2 | 0.4849 | Genome-1 | Genome-FC2 | C | 4761 | T |
| Internal Capsule | 0.0231 | Genome-1 | Genome-FC2 | C | 4761 | T |
| Frontal Cortex 3 | 0.027 | Genome-1 | Genome-FC2 | C | 4761 | T |
| Hippocampus | 0 | Genome-1 | Genome-FC2 | C | 4761 | T |
| Temporal Lobe | 0 | Genome-1 | cluster 3 | A | 4797 | G |
| Parietal Lobe | 0.3581 | Genome-1 | cluster 3 | A | 4797 | G |
| Upper Brain Stem | 0.0273 | Genome-1 | cluster 3 | A | 4797 | G |
| Midbrain | 0 | Genome-1 | cluster 3 | A | 4797 | G |
| Frontal Cortex 1 | 0 | Genome-1 | cluster 3 | A | 4797 | G |
| SSPE 2 | 0 | Genome-1 | cluster 3 | A | 4797 | G |
| Brain Stem | 0 | Genome-1 | cluster 3 | A | 4797 | G |
| Occipital Lobe | 0 | Genome-1 | cluster 3 | A | 4797 | G |
| SSPE 1 | 0 | Genome-1 | cluster 3 | A | 4797 | G |
| Cerebellum | 0 | Genome-1 | cluster 3 | A | 4797 | G |
| Cerebellum Nucleus | 0 | Genome-1 | cluster 3 | A | 4797 | G |
| Frontal Cortex 2 | 0 | Genome-1 | cluster 3 | A | 4797 | G |
| Internal Capsule | 0.0518 | Genome-1 | cluster 3 | A | 4797 | G |
| Frontal Cortex 3 | 0 | Genome-1 | cluster 3 | A | 4797 | G |
| Hippocampus | 0.0333 | Genome-1 | cluster 3 | A | 4797 | G |
| Temporal Lobe | 0 | Genome-1 | cluster 3 | G | 5126 | A |
| Parietal Lobe | 0.4965 | Genome-1 | cluster 3 | G | 5126 | A |
| Upper Brain Stem | 0.0493 | Genome-1 | cluster 3 | G | 5126 | A |
| Midbrain | 0 | Genome-1 | cluster 3 | G | 5126 | A |
| Frontal Cortex 1 | 0 | Genome-1 | cluster 3 | G | 5126 | A |
| SSPE 2 | 0 | Genome-1 | cluster 3 | G | 5126 | A |
| Brain Stem | 0 | Genome-1 | cluster 3 | G | 5126 | A |
| Occipital Lobe | 0 | Genome-1 | cluster 3 | G | 5126 | A |
| SSPE 1 | 0 | Genome-1 | cluster 3 | G | 5126 | A |
| Cerebellum | 0 | Genome-1 | cluster 3 | G | 5126 | A |
| Cerebellum Nucleus | 0 | Genome-1 | cluster 3 | G | 5126 | A |
| Frontal Cortex 2 | 0 | Genome-1 | cluster 3 | G | 5126 | A |
| Internal Capsule | 0.188811 | Genome-1 | cluster 3 | G | 5126 | A |
| Frontal Cortex 3 | 0 | Genome-1 | cluster 3 | G | 5126 | A |
| Hippocampus | 0.0913 | Genome-1 | cluster 3 | G | 5126 | A |
| Temporal Lobe | 0 | Genome-1 | cluster 3 | C | 5250 | T |
| Parietal Lobe | 0.5465 | Genome-1 | cluster 3 | C | 5250 | T |
| Upper Brain Stem | 0.0425 | Genome-1 | cluster 3 | C | 5250 | T |
| Midbrain | 0 | Genome-1 | cluster 3 | C | 5250 | T |
| Frontal Cortex 1 | 0 | Genome-1 | cluster 3 | C | 5250 | T |
| SSPE 2 | 0 | Genome-1 | cluster 3 | C | 5250 | T |
| Brain Stem | 0.0256 | Genome-1 | cluster 3 | C | 5250 | T |
| Occipital Lobe | 0 | Genome-1 | cluster 3 | C | 5250 | T |
| SSPE 1 | 0 | Genome-1 | cluster 3 | C | 5250 | T |
| Cerebellum | 0 | Genome-1 | cluster 3 | C | 5250 | T |
| Cerebellum Nucleus | 0 | Genome-1 | cluster 3 | C | 5250 | T |
| Frontal Cortex 2 | 0 | Genome-1 | cluster 3 | C | 5250 | T |
| Internal Capsule | 0.1969 | Genome-1 | cluster 3 | C | 5250 | T |
| Frontal Cortex 3 | 0 | Genome-1 | cluster 3 | C | 5250 | T |
| Hippocampus | 0.0793 | Genome-1 | cluster 3 | C | 5250 | T |
| Temporal Lobe | 0 | Genome-1 | Genome-FC2 | T | 7304 | C |
| Parietal Lobe | 0.0374 | Genome-1 | Genome-FC2 | T | 7304 | C |
| Upper Brain Stem | 0 | Genome-1 | Genome-FC2 | T | 7304 | C |
| Midbrain | 0 | Genome-1 | Genome-FC2 | T | 7304 | C |
| Frontal Cortex 1 | 0 | Genome-1 | Genome-FC2 | T | 7304 | C |
| SSPE 2 | 0 | Genome-1 | Genome-FC2 | T | 7304 | C |
| Brain Stem | 0 | Genome-1 | Genome-FC2 | T | 7304 | C |
| Occipital Lobe | 0.024 | Genome-1 | Genome-FC2 | T | 7304 | C |
| SSPE 1 | 0 | Genome-1 | Genome-FC2 | T | 7304 | C |
| Cerebellum | 0 | Genome-1 | Genome-FC2 | T | 7304 | C |
| Cerebellum Nucleus | 0 | Genome-1 | Genome-FC2 | T | 7304 | C |
| Frontal Cortex 2 | 0.4469 | Genome-1 | Genome-FC2 | T | 7304 | C |
| Internal Capsule | 0.053 | Genome-1 | Genome-FC2 | T | 7304 | C |
| Frontal Cortex 3 | 0 | Genome-1 | Genome-FC2 | T | 7304 | C |
| Hippocampus | 0 | Genome-1 | Genome-FC2 | T | 7304 | C |
| Temporal Lobe | 0 | Genome-1 | cluster 3 | A | 7861 | C |
| Parietal Lobe | 0.4774 | Genome-1 | cluster 3 | A | 7861 | C |
| Upper Brain Stem | 0.028 | Genome-1 | cluster 3 | A | 7861 | C |
| Midbrain | 0 | Genome-1 | cluster 3 | A | 7861 | C |
| Frontal Cortex 1 | 0 | Genome-1 | cluster 3 | A | 7861 | C |
| SSPE 2 | 0 | Genome-1 | cluster 3 | A | 7861 | C |
| Brain Stem | 0.020585 | Genome-1 | cluster 3 | A | 7861 | C |
| Occipital Lobe | 0 | Genome-1 | cluster 3 | A | 7861 | C |
| SSPE 1 | 0 | Genome-1 | cluster 3 | A | 7861 | C |
| Cerebellum | 0 | Genome-1 | cluster 3 | A | 7861 | C |
| Cerebellum Nucleus | 0.0233 | Genome-1 | cluster 3 | A | 7861 | C |
| Frontal Cortex 2 | 0 | Genome-1 | cluster 3 | A | 7861 | C |
| Internal Capsule | 0.1152 | Genome-1 | cluster 3 | A | 7861 | C |
| Frontal Cortex 3 | 0 | Genome-1 | cluster 3 | A | 7861 | C |
| Hippocampus | 0.0516 | Genome-1 | cluster 3 | A | 7861 | C |
| Temporal Lobe | 0 | Genome-1 | cluster 5 | C | 8292 | T |
| Parietal Lobe | 0.1138 | Genome-1 | cluster 5 | C | 8292 | T |
| Upper Brain Stem | 0.0689 | Genome-1 | cluster 5 | C | 8292 | T |
| Midbrain | 0 | Genome-1 | cluster 5 | C | 8292 | T |
| Frontal Cortex 1 | 0 | Genome-1 | cluster 5 | C | 8292 | T |
| SSPE 2 | 0 | Genome-1 | cluster 5 | C | 8292 | T |
| Brain Stem | 0.1411 | Genome-1 | cluster 5 | C | 8292 | T |
| Occipital Lobe | 0 | Genome-1 | cluster 5 | C | 8292 | T |
| SSPE 1 | 0 | Genome-1 | cluster 5 | C | 8292 | T |
| Cerebellum | 0.043 | Genome-1 | cluster 5 | C | 8292 | T |
| Cerebellum Nucleus | 0.0457 | Genome-1 | cluster 5 | C | 8292 | T |
| Frontal Cortex 2 | 0 | Genome-1 | cluster 5 | C | 8292 | T |
| Internal Capsule | 0.1976 | Genome-1 | cluster 5 | C | 8292 | T |
| Frontal Cortex 3 | 0 | Genome-1 | cluster 5 | C | 8292 | T |
| Hippocampus | 0.0608 | Genome-1 | cluster 5 | C | 8292 | T |
| Temporal Lobe | 0 | Genome-2 | cluster 8 | A | 9563 | T |
| Parietal Lobe | 0.1493 | Genome-2 | cluster 8 | A | 9563 | T |
| Upper Brain Stem | 0 | Genome-2 | cluster 8 | A | 9563 | T |
| Midbrain | 0 | Genome-2 | cluster 8 | A | 9563 | T |
| Frontal Cortex 1 | 0.3135 | Genome-2 | cluster 8 | A | 9563 | T |
| SSPE 2 | 0.2542 | Genome-2 | cluster 8 | A | 9563 | T |
| Brain Stem | 0 | Genome-2 | cluster 8 | A | 9563 | T |
| Occipital Lobe | 0 | Genome-2 | cluster 8 | A | 9563 | T |
| SSPE 1 | 0.2635 | Genome-2 | cluster 8 | A | 9563 | T |
| Cerebellum | 0 | Genome-2 | cluster 8 | A | 9563 | T |
| Cerebellum Nucleus | 0.0368 | Genome-2 | cluster 8 | A | 9563 | T |
| Frontal Cortex 2 | 0 | Genome-2 | cluster 8 | A | 9563 | T |
| Internal Capsule | 0.082 | Genome-2 | cluster 8 | A | 9563 | T |
| Frontal Cortex 3 | 0.3234 | Genome-2 | cluster 8 | A | 9563 | T |
| Hippocampus | 0.1384 | Genome-2 | cluster 8 | A | 9563 | T |
| Temporal Lobe | 0 | Genome-1 | cluster 3 | A | 14342 | C |
| Parietal Lobe | 0.4713 | Genome-1 | cluster 3 | A | 14342 | C |
| Upper Brain Stem | 0.031 | Genome-1 | cluster 3 | A | 14342 | C |
| Midbrain | 0 | Genome-1 | cluster 3 | A | 14342 | C |
| Frontal Cortex 1 | 0 | Genome-1 | cluster 3 | A | 14342 | C |
| SSPE 2 | 0 | Genome-1 | cluster 3 | A | 14342 | C |
| Brain Stem | 0.020357 | Genome-1 | cluster 3 | A | 14342 | C |
| Occipital Lobe | 0 | Genome-1 | cluster 3 | A | 14342 | C |
| SSPE 1 | 0 | Genome-1 | cluster 3 | A | 14342 | C |
| Cerebellum | 0 | Genome-1 | cluster 3 | A | 14342 | C |
| Cerebellum Nucleus | 0.023397 | Genome-1 | cluster 3 | A | 14342 | C |
| Frontal Cortex 2 | 0 | Genome-1 | cluster 3 | A | 14342 | C |
| Internal Capsule | 0.1124 | Genome-1 | cluster 3 | A | 14342 | C |
| Frontal Cortex 3 | 0 | Genome-1 | cluster 3 | A | 14342 | C |
| Hippocampus | 0.0483 | Genome-1 | cluster 3 | A | 14342 | C |
| Temporal Lobe | 0 | Genome-1 | cluster 6 | T | 332 | C |
| Parietal Lobe | 0 | Genome-1 | cluster 6 | T | 332 | C |
| Upper Brain Stem | 0.0278 | Genome-1 | cluster 6 | T | 332 | C |
| Midbrain | 0.0784 | Genome-1 | cluster 6 | T | 332 | C |
| Frontal Cortex 1 | 0 | Genome-1 | cluster 6 | T | 332 | C |
| SSPE 2 | 0 | Genome-1 | cluster 6 | T | 332 | C |
| Brain Stem | 0.1165 | Genome-1 | cluster 6 | T | 332 | C |
| Occipital Lobe | 0 | Genome-1 | cluster 6 | T | 332 | C |
| SSPE 1 | 0 | Genome-1 | cluster 6 | T | 332 | C |
| Cerebellum | 0.16 | Genome-1 | cluster 6 | T | 332 | C |
| Cerebellum Nucleus | 0.0628 | Genome-1 | cluster 6 | T | 332 | C |
| Frontal Cortex 2 | 0 | Genome-1 | cluster 6 | T | 332 | C |
| Internal Capsule | 0 | Genome-1 | cluster 6 | T | 332 | C |
| Frontal Cortex 3 | 0 | Genome-1 | cluster 6 | T | 332 | C |
| Hippocampus | 0 | Genome-1 | cluster 6 | T | 332 | C |
| Temporal Lobe | 0 | Genome-1 | cluster 4 | C | 684 | T |
| Parietal Lobe | 0 | Genome-1 | cluster 4 | C | 684 | T |
| Upper Brain Stem | 0.3793 | Genome-1 | cluster 4 | C | 684 | T |
| Midbrain | 0.1875 | Genome-1 | cluster 4 | C | 684 | T |
| Frontal Cortex 1 | 0.1233 | Genome-1 | cluster 4 | C | 684 | T |
| SSPE 2 | 0.0534 | Genome-1 | cluster 4 | C | 684 | T |
| Brain Stem | 0.1394 | Genome-1 | cluster 4 | C | 684 | T |
| Occipital Lobe | 0 | Genome-1 | cluster 4 | C | 684 | T |
| SSPE 1 | 0.114 | Genome-1 | cluster 4 | C | 684 | T |
| Cerebellum | 0.046 | Genome-1 | cluster 4 | C | 684 | T |
| Cerebellum Nucleus | 0.0814 | Genome-1 | cluster 4 | C | 684 | T |
| Frontal Cortex 2 | 0 | Genome-1 | cluster 4 | C | 684 | T |
| Internal Capsule | 0 | Genome-1 | cluster 4 | C | 684 | T |
| Frontal Cortex 3 | 0.122 | Genome-1 | cluster 4 | C | 684 | T |
| Hippocampus | 0 | Genome-1 | cluster 4 | C | 684 | T |
| Temporal Lobe | 0 | Genome-1 | cluster 2 | T | 2343 | C |
| Parietal Lobe | 0 | Genome-1 | cluster 2 | T | 2343 | C |
| Upper Brain Stem | 0.0972 | Genome-1 | cluster 2 | T | 2343 | C |
| Midbrain | 0.3052 | Genome-1 | cluster 2 | T | 2343 | C |
| Frontal Cortex 1 | 0 | Genome-1 | cluster 2 | T | 2343 | C |
| SSPE 2 | 0 | Genome-1 | cluster 2 | T | 2343 | C |
| Brain Stem | 0.416 | Genome-1 | cluster 2 | T | 2343 | C |
| Occipital Lobe | 0 | Genome-1 | cluster 2 | T | 2343 | C |
| SSPE 1 | 0.0537 | Genome-1 | cluster 2 | T | 2343 | C |
| Cerebellum | 0.4886 | Genome-1 | cluster 2 | T | 2343 | C |
| Cerebellum Nucleus | 0.35 | Genome-1 | cluster 2 | T | 2343 | C |
| Frontal Cortex 2 | 0 | Genome-1 | cluster 2 | T | 2343 | C |
| Internal Capsule | 0 | Genome-1 | cluster 2 | T | 2343 | C |
| Frontal Cortex 3 | 0 | Genome-1 | cluster 2 | T | 2343 | C |
| Hippocampus | 0 | Genome-1 | cluster 2 | T | 2343 | C |
| Temporal Lobe | 0 | Genome-1 | cluster 2 | T | 2349 | C |
| Parietal Lobe | 0 | Genome-1 | cluster 2 | T | 2349 | C |
| Upper Brain Stem | 0.0959 | Genome-1 | cluster 2 | T | 2349 | C |
| Midbrain | 0.3043 | Genome-1 | cluster 2 | T | 2349 | C |
| Frontal Cortex 1 | 0 | Genome-1 | cluster 2 | T | 2349 | C |
| SSPE 2 | 0 | Genome-1 | cluster 2 | T | 2349 | C |
| Brain Stem | 0.4175 | Genome-1 | cluster 2 | T | 2349 | C |
| Occipital Lobe | 0 | Genome-1 | cluster 2 | T | 2349 | C |
| SSPE 1 | 0 | Genome-1 | cluster 2 | T | 2349 | C |
| Cerebellum | 0.4819 | Genome-1 | cluster 2 | T | 2349 | C |
| Cerebellum Nucleus | 0.3526 | Genome-1 | cluster 2 | T | 2349 | C |
| Frontal Cortex 2 | 0 | Genome-1 | cluster 2 | T | 2349 | C |
| Internal Capsule | 0 | Genome-1 | cluster 2 | T | 2349 | C |
| Frontal Cortex 3 | 0 | Genome-1 | cluster 2 | T | 2349 | C |
| Hippocampus | 0 | Genome-1 | cluster 2 | T | 2349 | C |
| Temporal Lobe | 0 | Genome-1 | cluster 2 | C | 3153 | T |
| Parietal Lobe | 0 | Genome-1 | cluster 2 | C | 3153 | T |
| Upper Brain Stem | 0.055 | Genome-1 | cluster 2 | C | 3153 | T |
| Midbrain | 0.2652 | Genome-1 | cluster 2 | C | 3153 | T |
| Frontal Cortex 1 | 0 | Genome-1 | cluster 2 | C | 3153 | T |
| SSPE 2 | 0 | Genome-1 | cluster 2 | C | 3153 | T |
| Brain Stem | 0.2472 | Genome-1 | cluster 2 | C | 3153 | T |
| Occipital Lobe | 0 | Genome-1 | cluster 2 | C | 3153 | T |
| SSPE 1 | 0 | Genome-1 | cluster 2 | C | 3153 | T |
| Cerebellum | 0.5047 | Genome-1 | cluster 2 | C | 3153 | T |
| Cerebellum Nucleus | 0.3228 | Genome-1 | cluster 2 | C | 3153 | T |
| Frontal Cortex 2 | 0 | Genome-1 | cluster 2 | C | 3153 | T |
| Internal Capsule | 0 | Genome-1 | cluster 2 | C | 3153 | T |
| Frontal Cortex 3 | 0 | Genome-1 | cluster 2 | C | 3153 | T |
| Hippocampus | 0 | Genome-1 | cluster 2 | C | 3153 | T |
| Temporal Lobe | 0 | Genome-2 | cluster 10 | T | 3761 | C |
| Parietal Lobe | 0 | Genome-2 | cluster 10 | T | 3761 | C |
| Upper Brain Stem | 0.0228 | Genome-2 | cluster 10 | T | 3761 | C |
| Midbrain | 0.237 | Genome-2 | cluster 10 | T | 3761 | C |
| Frontal Cortex 1 | 0 | Genome-2 | cluster 10 | T | 3761 | C |
| SSPE 2 | 0 | Genome-2 | cluster 10 | T | 3761 | C |
| Brain Stem | 0.0288 | Genome-2 | cluster 10 | T | 3761 | C |
| Occipital Lobe | 0 | Genome-2 | cluster 10 | T | 3761 | C |
| SSPE 1 | 0 | Genome-2 | cluster 10 | T | 3761 | C |
| Cerebellum | 0.0446 | Genome-2 | cluster 10 | T | 3761 | C |
| Cerebellum Nucleus | 0.1643 | Genome-2 | cluster 10 | T | 3761 | C |
| Frontal Cortex 2 | 0 | Genome-2 | cluster 10 | T | 3761 | C |
| Internal Capsule | 0 | Genome-2 | cluster 10 | T | 3761 | C |
| Frontal Cortex 3 | 0 | Genome-2 | cluster 10 | T | 3761 | C |
| Hippocampus | 0 | Genome-2 | cluster 10 | T | 3761 | C |
| Temporal Lobe | 0 | Genome-1 | cluster 2 | A | 3827 | G |
| Parietal Lobe | 0 | Genome-1 | cluster 2 | A | 3827 | G |
| Upper Brain Stem | 0.0893 | Genome-1 | cluster 2 | A | 3827 | G |
| Midbrain | 0.2831 | Genome-1 | cluster 2 | A | 3827 | G |
| Frontal Cortex 1 | 0 | Genome-1 | cluster 2 | A | 3827 | G |
| SSPE 2 | 0 | Genome-1 | cluster 2 | A | 3827 | G |
| Brain Stem | 0.395 | Genome-1 | cluster 2 | A | 3827 | G |
| Occipital Lobe | 0 | Genome-1 | cluster 2 | A | 3827 | G |
| SSPE 1 | 0 | Genome-1 | cluster 2 | A | 3827 | G |
| Cerebellum | 0.4931 | Genome-1 | cluster 2 | A | 3827 | G |
| Cerebellum Nucleus | 0.3045 | Genome-1 | cluster 2 | A | 3827 | G |
| Frontal Cortex 2 | 0 | Genome-1 | cluster 2 | A | 3827 | G |
| Internal Capsule | 0 | Genome-1 | cluster 2 | A | 3827 | G |
| Frontal Cortex 3 | 0 | Genome-1 | cluster 2 | A | 3827 | G |
| Hippocampus | 0 | Genome-1 | cluster 2 | A | 3827 | G |
| Temporal Lobe | 0 | Genome-1 | Genome-FC2 | C | 3874 | T |
| Parietal Lobe | 0 | Genome-1 | Genome-FC2 | C | 3874 | T |
| Upper Brain Stem | 0.0211 | Genome-1 | Genome-FC2 | C | 3874 | T |
| Midbrain | 0 | Genome-1 | Genome-FC2 | C | 3874 | T |
| Frontal Cortex 1 | 0 | Genome-1 | Genome-FC2 | C | 3874 | T |
| SSPE 2 | 0 | Genome-1 | Genome-FC2 | C | 3874 | T |
| Brain Stem | 0 | Genome-1 | Genome-FC2 | C | 3874 | T |
| Occipital Lobe | 0 | Genome-1 | Genome-FC2 | C | 3874 | T |
| SSPE 1 | 0 | Genome-1 | Genome-FC2 | C | 3874 | T |
| Cerebellum | 0 | Genome-1 | Genome-FC2 | C | 3874 | T |
| Cerebellum Nucleus | 0 | Genome-1 | Genome-FC2 | C | 3874 | T |
| Frontal Cortex 2 | 0.3688 | Genome-1 | Genome-FC2 | C | 3874 | T |
| Internal Capsule | 0.0418 | Genome-1 | Genome-FC2 | C | 3874 | T |
| Frontal Cortex 3 | 0 | Genome-1 | Genome-FC2 | C | 3874 | T |
| Hippocampus | 0 | Genome-1 | Genome-FC2 | C | 3874 | T |
| Temporal Lobe | 0 | Genome-1 | Genome-FC2 | C | 4700 | T |
| Parietal Lobe | 0.020524 | Genome-1 | Genome-FC2 | C | 4700 | T |
| Upper Brain Stem | 0.0409 | Genome-1 | Genome-FC2 | C | 4700 | T |
| Midbrain | 0 | Genome-1 | Genome-FC2 | C | 4700 | T |
| Frontal Cortex 1 | 0.0261 | Genome-1 | Genome-FC2 | C | 4700 | T |
| SSPE 2 | 0 | Genome-1 | Genome-FC2 | C | 4700 | T |
| Brain Stem | 0 | Genome-1 | Genome-FC2 | C | 4700 | T |
| Occipital Lobe | 0.0205 | Genome-1 | Genome-FC2 | C | 4700 | T |
| SSPE 1 | 0 | Genome-1 | Genome-FC2 | C | 4700 | T |
| Cerebellum | 0 | Genome-1 | Genome-FC2 | C | 4700 | T |
| Cerebellum Nucleus | 0.101 | Genome-1 | Genome-FC2 | C | 4700 | T |
| Frontal Cortex 2 | 0.358 | Genome-1 | Genome-FC2 | C | 4700 | T |
| Internal Capsule | 0.0628 | Genome-1 | Genome-FC2 | C | 4700 | T |
| Frontal Cortex 3 | 0.0331 | Genome-1 | Genome-FC2 | C | 4700 | T |
| Hippocampus | 0.021277 | Genome-1 | Genome-FC2 | C | 4700 | T |
| Temporal Lobe | 0 | Genome-1 | cluster 2 | C | 4834 | T |
| Parietal Lobe | 0 | Genome-1 | cluster 2 | C | 4834 | T |
| Upper Brain Stem | 0.0962 | Genome-1 | cluster 2 | C | 4834 | T |
| Midbrain | 0.3109 | Genome-1 | cluster 2 | C | 4834 | T |
| Frontal Cortex 1 | 0 | Genome-1 | cluster 2 | C | 4834 | T |
| SSPE 2 | 0 | Genome-1 | cluster 2 | C | 4834 | T |
| Brain Stem | 0.4284 | Genome-1 | cluster 2 | C | 4834 | T |
| Occipital Lobe | 0 | Genome-1 | cluster 2 | C | 4834 | T |
| SSPE 1 | 0 | Genome-1 | cluster 2 | C | 4834 | T |
| Cerebellum | 0.523077 | Genome-1 | cluster 2 | C | 4834 | T |
| Cerebellum Nucleus | 0.3411 | Genome-1 | cluster 2 | C | 4834 | T |
| Frontal Cortex 2 | 0 | Genome-1 | cluster 2 | C | 4834 | T |
| Internal Capsule | 0 | Genome-1 | cluster 2 | C | 4834 | T |
| Frontal Cortex 3 | 0 | Genome-1 | cluster 2 | C | 4834 | T |
| Hippocampus | 0 | Genome-1 | cluster 2 | C | 4834 | T |
| Temporal Lobe | 0 | Genome-1 | cluster 2 | G | 5368 | T |
| Parietal Lobe | 0 | Genome-1 | cluster 2 | G | 5368 | T |
| Upper Brain Stem | 0.0663 | Genome-1 | cluster 2 | G | 5368 | T |
| Midbrain | 0.2368 | Genome-1 | cluster 2 | G | 5368 | T |
| Frontal Cortex 1 | 0 | Genome-1 | cluster 2 | G | 5368 | T |
| SSPE 2 | 0 | Genome-1 | cluster 2 | G | 5368 | T |
| Brain Stem | 0.376 | Genome-1 | cluster 2 | G | 5368 | T |
| Occipital Lobe | 0 | Genome-1 | cluster 2 | G | 5368 | T |
| SSPE 1 | 0 | Genome-1 | cluster 2 | G | 5368 | T |
| Cerebellum | 0.458824 | Genome-1 | cluster 2 | G | 5368 | T |
| Cerebellum Nucleus | 0.2228 | Genome-1 | cluster 2 | G | 5368 | T |
| Frontal Cortex 2 | 0 | Genome-1 | cluster 2 | G | 5368 | T |
| Internal Capsule | 0 | Genome-1 | cluster 2 | G | 5368 | T |
| Frontal Cortex 3 | 0 | Genome-1 | cluster 2 | G | 5368 | T |
| Hippocampus | 0 | Genome-1 | cluster 2 | G | 5368 | T |
| Temporal Lobe | 0 | Genome-1 | cluster 4 | T | 5398 | C |
| Parietal Lobe | 0 | Genome-1 | cluster 4 | T | 5398 | C |
| Upper Brain Stem | 0.315 | Genome-1 | cluster 4 | T | 5398 | C |
| Midbrain | 0.0683 | Genome-1 | cluster 4 | T | 5398 | C |
| Frontal Cortex 1 | 0.0688 | Genome-1 | cluster 4 | T | 5398 | C |
| SSPE 2 | 0 | Genome-1 | cluster 4 | T | 5398 | C |
| Brain Stem | 0.1142 | Genome-1 | cluster 4 | T | 5398 | C |
| Occipital Lobe | 0 | Genome-1 | cluster 4 | T | 5398 | C |
| SSPE 1 | 0 | Genome-1 | cluster 4 | T | 5398 | C |
| Cerebellum | 0.028 | Genome-1 | cluster 4 | T | 5398 | C |
| Cerebellum Nucleus | 0.0202 | Genome-1 | cluster 4 | T | 5398 | C |
| Frontal Cortex 2 | 0 | Genome-1 | cluster 4 | T | 5398 | C |
| Internal Capsule | 0 | Genome-1 | cluster 4 | T | 5398 | C |
| Frontal Cortex 3 | 0.0667 | Genome-1 | cluster 4 | T | 5398 | C |
| Hippocampus | 0 | Genome-1 | cluster 4 | T | 5398 | C |
| Temporal Lobe | 0 | Genome-1 | cluster 4 | T | 5409 | C |
| Parietal Lobe | 0 | Genome-1 | cluster 4 | T | 5409 | C |
| Upper Brain Stem | 0.3089 | Genome-1 | cluster 4 | T | 5409 | C |
| Midbrain | 0.0684 | Genome-1 | cluster 4 | T | 5409 | C |
| Frontal Cortex 1 | 0.0388 | Genome-1 | cluster 4 | T | 5409 | C |
| SSPE 2 | 0 | Genome-1 | cluster 4 | T | 5409 | C |
| Brain Stem | 0.1134 | Genome-1 | cluster 4 | T | 5409 | C |
| Occipital Lobe | 0 | Genome-1 | cluster 4 | T | 5409 | C |
| SSPE 1 | 0 | Genome-1 | cluster 4 | T | 5409 | C |
| Cerebellum | 0.025362 | Genome-1 | cluster 4 | T | 5409 | C |
| Cerebellum Nucleus | 0.027 | Genome-1 | cluster 4 | T | 5409 | C |
| Frontal Cortex 2 | 0 | Genome-1 | cluster 4 | T | 5409 | C |
| Internal Capsule | 0 | Genome-1 | cluster 4 | T | 5409 | C |
| Frontal Cortex 3 | 0.0392 | Genome-1 | cluster 4 | T | 5409 | C |
| Hippocampus | 0 | Genome-1 | cluster 4 | T | 5409 | C |
| Temporal Lobe | 0 | Genome-2 | cluster 10 | G | 5961 | A |
| Parietal Lobe | 0 | Genome-2 | cluster 10 | G | 5961 | A |
| Upper Brain Stem | 0.0259 | Genome-2 | cluster 10 | G | 5961 | A |
| Midbrain | 0.2437 | Genome-2 | cluster 10 | G | 5961 | A |
| Frontal Cortex 1 | 0 | Genome-2 | cluster 10 | G | 5961 | A |
| SSPE 2 | 0 | Genome-2 | cluster 10 | G | 5961 | A |
| Brain Stem | 0.0262 | Genome-2 | cluster 10 | G | 5961 | A |
| Occipital Lobe | 0 | Genome-2 | cluster 10 | G | 5961 | A |
| SSPE 1 | 0 | Genome-2 | cluster 10 | G | 5961 | A |
| Cerebellum | 0.0712 | Genome-2 | cluster 10 | G | 5961 | A |
| Cerebellum Nucleus | 0.2095 | Genome-2 | cluster 10 | G | 5961 | A |
| Frontal Cortex 2 | 0 | Genome-2 | cluster 10 | G | 5961 | A |
| Internal Capsule | 0 | Genome-2 | cluster 10 | G | 5961 | A |
| Frontal Cortex 3 | 0 | Genome-2 | cluster 10 | G | 5961 | A |
| Hippocampus | 0 | Genome-2 | cluster 10 | G | 5961 | A |
| Temporal Lobe | 0 | Genome-2 | cluster 10 | G | 6123 | A |
| Parietal Lobe | 0 | Genome-2 | cluster 10 | G | 6123 | A |
| Upper Brain Stem | 0.0282 | Genome-2 | cluster 10 | G | 6123 | A |
| Midbrain | 0.2458 | Genome-2 | cluster 10 | G | 6123 | A |
| Frontal Cortex 1 | 0 | Genome-2 | cluster 10 | G | 6123 | A |
| SSPE 2 | 0 | Genome-2 | cluster 10 | G | 6123 | A |
| Brain Stem | 0.0298 | Genome-2 | cluster 10 | G | 6123 | A |
| Occipital Lobe | 0 | Genome-2 | cluster 10 | G | 6123 | A |
| SSPE 1 | 0 | Genome-2 | cluster 10 | G | 6123 | A |
| Cerebellum | 0.0685 | Genome-2 | cluster 10 | G | 6123 | A |
| Cerebellum Nucleus | 0.2091 | Genome-2 | cluster 10 | G | 6123 | A |
| Frontal Cortex 2 | 0 | Genome-2 | cluster 10 | G | 6123 | A |
| Internal Capsule | 0 | Genome-2 | cluster 10 | G | 6123 | A |
| Frontal Cortex 3 | 0 | Genome-2 | cluster 10 | G | 6123 | A |
| Hippocampus | 0 | Genome-2 | cluster 10 | G | 6123 | A |
| Temporal Lobe | 0 | Genome-1 | cluster 2 | C | 6774 | A |
| Parietal Lobe | 0 | Genome-1 | cluster 2 | C | 6774 | A |
| Upper Brain Stem | 0.0869 | Genome-1 | cluster 2 | C | 6774 | A |
| Midbrain | 0.266 | Genome-1 | cluster 2 | C | 6774 | A |
| Frontal Cortex 1 | 0 | Genome-1 | cluster 2 | C | 6774 | A |
| SSPE 2 | 0 | Genome-1 | cluster 2 | C | 6774 | A |
| Brain Stem | 0.4255 | Genome-1 | cluster 2 | C | 6774 | A |
| Occipital Lobe | 0 | Genome-1 | cluster 2 | C | 6774 | A |
| SSPE 1 | 0 | Genome-1 | cluster 2 | C | 6774 | A |
| Cerebellum | 0.5537 | Genome-1 | cluster 2 | C | 6774 | A |
| Cerebellum Nucleus | 0.2692 | Genome-1 | cluster 2 | C | 6774 | A |
| Frontal Cortex 2 | 0 | Genome-1 | cluster 2 | C | 6774 | A |
| Internal Capsule | 0 | Genome-1 | cluster 2 | C | 6774 | A |
| Frontal Cortex 3 | 0 | Genome-1 | cluster 2 | C | 6774 | A |
| Hippocampus | 0 | Genome-1 | cluster 2 | C | 6774 | A |
| Temporal Lobe | 0 | Genome-2 | cluster 10 | C | 6776 | A |
| Parietal Lobe | 0 | Genome-2 | cluster 10 | C | 6776 | A |
| Upper Brain Stem | 0.0266 | Genome-2 | cluster 10 | C | 6776 | A |
| Midbrain | 0.2559 | Genome-2 | cluster 10 | C | 6776 | A |
| Frontal Cortex 1 | 0 | Genome-2 | cluster 10 | C | 6776 | A |
| SSPE 2 | 0 | Genome-2 | cluster 10 | C | 6776 | A |
| Brain Stem | 0.0275 | Genome-2 | cluster 10 | C | 6776 | A |
| Occipital Lobe | 0 | Genome-2 | cluster 10 | C | 6776 | A |
| SSPE 1 | 0 | Genome-2 | cluster 10 | C | 6776 | A |
| Cerebellum | 0.0539 | Genome-2 | cluster 10 | C | 6776 | A |
| Cerebellum Nucleus | 0.2233 | Genome-2 | cluster 10 | C | 6776 | A |
| Frontal Cortex 2 | 0 | Genome-2 | cluster 10 | C | 6776 | A |
| Internal Capsule | 0 | Genome-2 | cluster 10 | C | 6776 | A |
| Frontal Cortex 3 | 0 | Genome-2 | cluster 10 | C | 6776 | A |
| Hippocampus | 0 | Genome-2 | cluster 10 | C | 6776 | A |
| Temporal Lobe | 0 | Genome-1 | cluster 1a | T | 7061 | C |
| Parietal Lobe | 0 | Genome-1 | cluster 1a | T | 7061 | C |
| Upper Brain Stem | 0.0363 | Genome-1 | cluster 1a | T | 7061 | C |
| Midbrain | 0.0439 | Genome-1 | cluster 1a | T | 7061 | C |
| Frontal Cortex 1 | 0.1323 | Genome-1 | cluster 1a | T | 7061 | C |
| SSPE 2 | 0.4077 | Genome-1 | cluster 1a | T | 7061 | C |
| Brain Stem | 0.1043 | Genome-1 | cluster 1a | T | 7061 | C |
| Occipital Lobe | 0.0246 | Genome-1 | cluster 1a | T | 7061 | C |
| SSPE 1 | 0.094 | Genome-1 | cluster 1a | T | 7061 | C |
| Cerebellum | 0.1286 | Genome-1 | cluster 1a | T | 7061 | C |
| Cerebellum Nucleus | 0.0489 | Genome-1 | cluster 1a | T | 7061 | C |
| Frontal Cortex 2 | 0 | Genome-1 | cluster 1a | T | 7061 | C |
| Internal Capsule | 0 | Genome-1 | cluster 1a | T | 7061 | C |
| Frontal Cortex 3 | 0.1293 | Genome-1 | cluster 1a | T | 7061 | C |
| Hippocampus | 0.0251 | Genome-1 | cluster 1a | T | 7061 | C |
| Temporal Lobe | 0 | Genome-1 | cluster 2 | C | 8346 | T |
| Parietal Lobe | 0 | Genome-1 | cluster 2 | C | 8346 | T |
| Upper Brain Stem | 0.0741 | Genome-1 | cluster 2 | C | 8346 | T |
| Midbrain | 0.2365 | Genome-1 | cluster 2 | C | 8346 | T |
| Frontal Cortex 1 | 0 | Genome-1 | cluster 2 | C | 8346 | T |
| SSPE 2 | 0 | Genome-1 | cluster 2 | C | 8346 | T |
| Brain Stem | 0.3837 | Genome-1 | cluster 2 | C | 8346 | T |
| Occipital Lobe | 0 | Genome-1 | cluster 2 | C | 8346 | T |
| SSPE 1 | 0 | Genome-1 | cluster 2 | C | 8346 | T |
| Cerebellum | 0.4508 | Genome-1 | cluster 2 | C | 8346 | T |
| Cerebellum Nucleus | 0.2694 | Genome-1 | cluster 2 | C | 8346 | T |
| Frontal Cortex 2 | 0 | Genome-1 | cluster 2 | C | 8346 | T |
| Internal Capsule | 0 | Genome-1 | cluster 2 | C | 8346 | T |
| Frontal Cortex 3 | 0 | Genome-1 | cluster 2 | C | 8346 | T |
| Hippocampus | 0 | Genome-1 | cluster 2 | C | 8346 | T |
| Temporal Lobe | 0 | Genome-1 | cluster 2 | C | 8525 | A |
| Parietal Lobe | 0 | Genome-1 | cluster 2 | C | 8525 | A |
| Upper Brain Stem | 0.0751 | Genome-1 | cluster 2 | C | 8525 | A |
| Midbrain | 0.2493 | Genome-1 | cluster 2 | C | 8525 | A |
| Frontal Cortex 1 | 0 | Genome-1 | cluster 2 | C | 8525 | A |
| SSPE 2 | 0 | Genome-1 | cluster 2 | C | 8525 | A |
| Brain Stem | 0.4154 | Genome-1 | cluster 2 | C | 8525 | A |
| Occipital Lobe | 0 | Genome-1 | cluster 2 | C | 8525 | A |
| SSPE 1 | 0 | Genome-1 | cluster 2 | C | 8525 | A |
| Cerebellum | 0.4387 | Genome-1 | cluster 2 | C | 8525 | A |
| Cerebellum Nucleus | 0.2353 | Genome-1 | cluster 2 | C | 8525 | A |
| Frontal Cortex 2 | 0 | Genome-1 | cluster 2 | C | 8525 | A |
| Internal Capsule | 0 | Genome-1 | cluster 2 | C | 8525 | A |
| Frontal Cortex 3 | 0 | Genome-1 | cluster 2 | C | 8525 | A |
| Hippocampus | 0 | Genome-1 | cluster 2 | C | 8525 | A |
| Temporal Lobe | 0 | Genome-1 | cluster 2 | A | 9786 | G |
| Parietal Lobe | 0 | Genome-1 | cluster 2 | A | 9786 | G |
| Upper Brain Stem | 0.0793 | Genome-1 | cluster 2 | A | 9786 | G |
| Midbrain | 0.2564 | Genome-1 | cluster 2 | A | 9786 | G |
| Frontal Cortex 1 | 0 | Genome-1 | cluster 2 | A | 9786 | G |
| SSPE 2 | 0 | Genome-1 | cluster 2 | A | 9786 | G |
| Brain Stem | 0.4444 | Genome-1 | cluster 2 | A | 9786 | G |
| Occipital Lobe | 0 | Genome-1 | cluster 2 | A | 9786 | G |
| SSPE 1 | 0 | Genome-1 | cluster 2 | A | 9786 | G |
| Cerebellum | 0.4545 | Genome-1 | cluster 2 | A | 9786 | G |
| Cerebellum Nucleus | 0.2618 | Genome-1 | cluster 2 | A | 9786 | G |
| Frontal Cortex 2 | 0 | Genome-1 | cluster 2 | A | 9786 | G |
| Internal Capsule | 0 | Genome-1 | cluster 2 | A | 9786 | G |
| Frontal Cortex 3 | 0 | Genome-1 | cluster 2 | A | 9786 | G |
| Hippocampus | 0 | Genome-1 | cluster 2 | A | 9786 | G |
| Temporal Lobe | 0 | Genome-1 | cluster 4 | T | 10826 | C |
| Parietal Lobe | 0 | Genome-1 | cluster 4 | T | 10826 | C |
| Upper Brain Stem | 0.3177 | Genome-1 | cluster 4 | T | 10826 | C |
| Midbrain | 0.068 | Genome-1 | cluster 4 | T | 10826 | C |
| Frontal Cortex 1 | 0 | Genome-1 | cluster 4 | T | 10826 | C |
| SSPE 2 | 0 | Genome-1 | cluster 4 | T | 10826 | C |
| Brain Stem | 0.1351 | Genome-1 | cluster 4 | T | 10826 | C |
| Occipital Lobe | 0 | Genome-1 | cluster 4 | T | 10826 | C |
| SSPE 1 | 0 | Genome-1 | cluster 4 | T | 10826 | C |
| Cerebellum | 0 | Genome-1 | cluster 4 | T | 10826 | C |
| Cerebellum Nucleus | 0 | Genome-1 | cluster 4 | T | 10826 | C |
| Frontal Cortex 2 | 0 | Genome-1 | cluster 4 | T | 10826 | C |
| Internal Capsule | 0 | Genome-1 | cluster 4 | T | 10826 | C |
| Frontal Cortex 3 | 0 | Genome-1 | cluster 4 | T | 10826 | C |
| Hippocampus | 0 | Genome-1 | cluster 4 | T | 10826 | C |
| Temporal Lobe | 0 | Genome-1 | cluster 4 | C | 11372 | T |
| Parietal Lobe | 0 | Genome-1 | cluster 4 | C | 11372 | T |
| Upper Brain Stem | 0.3396 | Genome-1 | cluster 4 | C | 11372 | T |
| Midbrain | 0.068 | Genome-1 | cluster 4 | C | 11372 | T |
| Frontal Cortex 1 | 0 | Genome-1 | cluster 4 | C | 11372 | T |
| SSPE 2 | 0 | Genome-1 | cluster 4 | C | 11372 | T |
| Brain Stem | 0.1369 | Genome-1 | cluster 4 | C | 11372 | T |
| Occipital Lobe | 0 | Genome-1 | cluster 4 | C | 11372 | T |
| SSPE 1 | 0 | Genome-1 | cluster 4 | C | 11372 | T |
| Cerebellum | 0 | Genome-1 | cluster 4 | C | 11372 | T |
| Cerebellum Nucleus | 0 | Genome-1 | cluster 4 | C | 11372 | T |
| Frontal Cortex 2 | 0 | Genome-1 | cluster 4 | C | 11372 | T |
| Internal Capsule | 0 | Genome-1 | cluster 4 | C | 11372 | T |
| Frontal Cortex 3 | 0 | Genome-1 | cluster 4 | C | 11372 | T |
| Hippocampus | 0 | Genome-1 | cluster 4 | C | 11372 | T |
| Temporal Lobe | 0 | Genome-2 | cluster 10 | A | 11732 | G |
| Parietal Lobe | 0 | Genome-2 | cluster 10 | A | 11732 | G |
| Upper Brain Stem | 0.0297 | Genome-2 | cluster 10 | A | 11732 | G |
| Midbrain | 0.2537 | Genome-2 | cluster 10 | A | 11732 | G |
| Frontal Cortex 1 | 0 | Genome-2 | cluster 10 | A | 11732 | G |
| SSPE 2 | 0 | Genome-2 | cluster 10 | A | 11732 | G |
| Brain Stem | 0.0247 | Genome-2 | cluster 10 | A | 11732 | G |
| Occipital Lobe | 0 | Genome-2 | cluster 10 | A | 11732 | G |
| SSPE 1 | 0 | Genome-2 | cluster 10 | A | 11732 | G |
| Cerebellum | 0.0579 | Genome-2 | cluster 10 | A | 11732 | G |
| Cerebellum Nucleus | 0.2259 | Genome-2 | cluster 10 | A | 11732 | G |
| Frontal Cortex 2 | 0 | Genome-2 | cluster 10 | A | 11732 | G |
| Internal Capsule | 0 | Genome-2 | cluster 10 | A | 11732 | G |
| Frontal Cortex 3 | 0 | Genome-2 | cluster 10 | A | 11732 | G |
| Hippocampus | 0 | Genome-2 | cluster 10 | A | 11732 | G |
| Temporal Lobe | 0 | Genome-1 | cluster 4 | A | 15050 | G |
| Parietal Lobe | 0 | Genome-1 | cluster 4 | A | 15050 | G |
| Upper Brain Stem | 0.2821 | Genome-1 | cluster 4 | A | 15050 | G |
| Midbrain | 0.0584 | Genome-1 | cluster 4 | A | 15050 | G |
| Frontal Cortex 1 | 0 | Genome-1 | cluster 4 | A | 15050 | G |
| SSPE 2 | 0 | Genome-1 | cluster 4 | A | 15050 | G |
| Brain Stem | 0.1088 | Genome-1 | cluster 4 | A | 15050 | G |
| Occipital Lobe | 0 | Genome-1 | cluster 4 | A | 15050 | G |
| SSPE 1 | 0 | Genome-1 | cluster 4 | A | 15050 | G |
| Cerebellum | 0 | Genome-1 | cluster 4 | A | 15050 | G |
| Cerebellum Nucleus | 0 | Genome-1 | cluster 4 | A | 15050 | G |
| Frontal Cortex 2 | 0 | Genome-1 | cluster 4 | A | 15050 | G |
| Internal Capsule | 0 | Genome-1 | cluster 4 | A | 15050 | G |
| Frontal Cortex 3 | 0 | Genome-1 | cluster 4 | A | 15050 | G |
| Hippocampus | 0 | Genome-1 | cluster 4 | A | 15050 | G |
| Temporal Lobe | 0 | Genome-1 | cluster 4 | A | 15362 | G |
| Parietal Lobe | 0 | Genome-1 | cluster 4 | A | 15362 | G |
| Upper Brain Stem | 0.3701 | Genome-1 | cluster 4 | A | 15362 | G |
| Midbrain | 0.2183 | Genome-1 | cluster 4 | A | 15362 | G |
| Frontal Cortex 1 | 0.1119 | Genome-1 | cluster 4 | A | 15362 | G |
| SSPE 2 | 0.0788 | Genome-1 | cluster 4 | A | 15362 | G |
| Brain Stem | 0.126 | Genome-1 | cluster 4 | A | 15362 | G |
| Occipital Lobe | 0 | Genome-1 | cluster 4 | A | 15362 | G |
| SSPE 1 | 0.102 | Genome-1 | cluster 4 | A | 15362 | G |
| Cerebellum | 0.0494 | Genome-1 | cluster 4 | A | 15362 | G |
| Cerebellum Nucleus | 0.0675 | Genome-1 | cluster 4 | A | 15362 | G |
| Frontal Cortex 2 | 0 | Genome-1 | cluster 4 | A | 15362 | G |
| Internal Capsule | 0 | Genome-1 | cluster 4 | A | 15362 | G |
| Frontal Cortex 3 | 0.1084 | Genome-1 | cluster 4 | A | 15362 | G |
| Hippocampus | 0 | Genome-1 | cluster 4 | A | 15362 | G |
| Temporal Lobe | 0 | Genome-1 | cluster 6 | T | 2543 | C |
| Parietal Lobe | 0 | Genome-1 | cluster 6 | T | 2543 | C |
| Upper Brain Stem | 0 | Genome-1 | cluster 6 | T | 2543 | C |
| Midbrain | 0.0437 | Genome-1 | cluster 6 | T | 2543 | C |
| Frontal Cortex 1 | 0 | Genome-1 | cluster 6 | T | 2543 | C |
| SSPE 2 | 0 | Genome-1 | cluster 6 | T | 2543 | C |
| Brain Stem | 0.097 | Genome-1 | cluster 6 | T | 2543 | C |
| Occipital Lobe | 0 | Genome-1 | cluster 6 | T | 2543 | C |
| SSPE 1 | 0 | Genome-1 | cluster 6 | T | 2543 | C |
| Cerebellum | 0.1379 | Genome-1 | cluster 6 | T | 2543 | C |
| Cerebellum Nucleus | 0.0415 | Genome-1 | cluster 6 | T | 2543 | C |
| Frontal Cortex 2 | 0 | Genome-1 | cluster 6 | T | 2543 | C |
| Internal Capsule | 0 | Genome-1 | cluster 6 | T | 2543 | C |
| Frontal Cortex 3 | 0 | Genome-1 | cluster 6 | T | 2543 | C |
| Hippocampus | 0 | Genome-1 | cluster 6 | T | 2543 | C |
| Temporal Lobe | 0 | Genome-2 | cluster 10 | G | 3579 | A |
| Parietal Lobe | 0 | Genome-2 | cluster 10 | G | 3579 | A |
| Upper Brain Stem | 0 | Genome-2 | cluster 10 | G | 3579 | A |
| Midbrain | 0.1715 | Genome-2 | cluster 10 | G | 3579 | A |
| Frontal Cortex 1 | 0 | Genome-2 | cluster 10 | G | 3579 | A |
| SSPE 2 | 0 | Genome-2 | cluster 10 | G | 3579 | A |
| Brain Stem | 0 | Genome-2 | cluster 10 | G | 3579 | A |
| Occipital Lobe | 0 | Genome-2 | cluster 10 | G | 3579 | A |
| SSPE 1 | 0 | Genome-2 | cluster 10 | G | 3579 | A |
| Cerebellum | 0.0287 | Genome-2 | cluster 10 | G | 3579 | A |
| Cerebellum Nucleus | 0.1118 | Genome-2 | cluster 10 | G | 3579 | A |
| Frontal Cortex 2 | 0 | Genome-2 | cluster 10 | G | 3579 | A |
| Internal Capsule | 0 | Genome-2 | cluster 10 | G | 3579 | A |
| Frontal Cortex 3 | 0 | Genome-2 | cluster 10 | G | 3579 | A |
| Hippocampus | 0 | Genome-2 | cluster 10 | G | 3579 | A |
| Temporal Lobe | 0 | Genome-1 | cluster 6 | A | 4304 | T |
| Parietal Lobe | 0 | Genome-1 | cluster 6 | A | 4304 | T |
| Upper Brain Stem | 0 | Genome-1 | cluster 6 | A | 4304 | T |
| Midbrain | 0.0437 | Genome-1 | cluster 6 | A | 4304 | T |
| Frontal Cortex 1 | 0 | Genome-1 | cluster 6 | A | 4304 | T |
| SSPE 2 | 0 | Genome-1 | cluster 6 | A | 4304 | T |
| Brain Stem | 0.0829 | Genome-1 | cluster 6 | A | 4304 | T |
| Occipital Lobe | 0 | Genome-1 | cluster 6 | A | 4304 | T |
| SSPE 1 | 0 | Genome-1 | cluster 6 | A | 4304 | T |
| Cerebellum | 0.1319 | Genome-1 | cluster 6 | A | 4304 | T |
| Cerebellum Nucleus | 0.0314 | Genome-1 | cluster 6 | A | 4304 | T |
| Frontal Cortex 2 | 0 | Genome-1 | cluster 6 | A | 4304 | T |
| Internal Capsule | 0 | Genome-1 | cluster 6 | A | 4304 | T |
| Frontal Cortex 3 | 0 | Genome-1 | cluster 6 | A | 4304 | T |
| Hippocampus | 0 | Genome-1 | cluster 6 | A | 4304 | T |
| Temporal Lobe | 0 | Genome-1 | cluster 6 | C | 5405 | T |
| Parietal Lobe | 0 | Genome-1 | cluster 6 | C | 5405 | T |
| Upper Brain Stem | 0 | Genome-1 | cluster 6 | C | 5405 | T |
| Midbrain | 0.0392 | Genome-1 | cluster 6 | C | 5405 | T |
| Frontal Cortex 1 | 0 | Genome-1 | cluster 6 | C | 5405 | T |
| SSPE 2 | 0 | Genome-1 | cluster 6 | C | 5405 | T |
| Brain Stem | 0.0785 | Genome-1 | cluster 6 | C | 5405 | T |
| Occipital Lobe | 0 | Genome-1 | cluster 6 | C | 5405 | T |
| SSPE 1 | 0 | Genome-1 | cluster 6 | C | 5405 | T |
| Cerebellum | 0.117188 | Genome-1 | cluster 6 | C | 5405 | T |
| Cerebellum Nucleus | 0.0327 | Genome-1 | cluster 6 | C | 5405 | T |
| Frontal Cortex 2 | 0 | Genome-1 | cluster 6 | C | 5405 | T |
| Internal Capsule | 0 | Genome-1 | cluster 6 | C | 5405 | T |
| Frontal Cortex 3 | 0 | Genome-1 | cluster 6 | C | 5405 | T |
| Hippocampus | 0 | Genome-1 | cluster 6 | C | 5405 | T |
| Temporal Lobe | 0 | Genome-1 | cluster 6 | T | 10790 | C |
| Parietal Lobe | 0 | Genome-1 | cluster 6 | T | 10790 | C |
| Upper Brain Stem | 0 | Genome-1 | cluster 6 | T | 10790 | C |
| Midbrain | 0.0358 | Genome-1 | cluster 6 | T | 10790 | C |
| Frontal Cortex 1 | 0 | Genome-1 | cluster 6 | T | 10790 | C |
| SSPE 2 | 0 | Genome-1 | cluster 6 | T | 10790 | C |
| Brain Stem | 0.0706 | Genome-1 | cluster 6 | T | 10790 | C |
| Occipital Lobe | 0 | Genome-1 | cluster 6 | T | 10790 | C |
| SSPE 1 | 0 | Genome-1 | cluster 6 | T | 10790 | C |
| Cerebellum | 0.1015 | Genome-1 | cluster 6 | T | 10790 | C |
| Cerebellum Nucleus | 0.0381 | Genome-1 | cluster 6 | T | 10790 | C |
| Frontal Cortex 2 | 0 | Genome-1 | cluster 6 | T | 10790 | C |
| Internal Capsule | 0 | Genome-1 | cluster 6 | T | 10790 | C |
| Frontal Cortex 3 | 0 | Genome-1 | cluster 6 | T | 10790 | C |
| Hippocampus | 0 | Genome-1 | cluster 6 | T | 10790 | C |
| Temporal Lobe | 0 | Genome-1 | cluster 6 | C | 13967 | T |
| Parietal Lobe | 0 | Genome-1 | cluster 6 | C | 13967 | T |
| Upper Brain Stem | 0 | Genome-1 | cluster 6 | C | 13967 | T |
| Midbrain | 0.0353 | Genome-1 | cluster 6 | C | 13967 | T |
| Frontal Cortex 1 | 0 | Genome-1 | cluster 6 | C | 13967 | T |
| SSPE 2 | 0 | Genome-1 | cluster 6 | C | 13967 | T |
| Brain Stem | 0.0683 | Genome-1 | cluster 6 | C | 13967 | T |
| Occipital Lobe | 0 | Genome-1 | cluster 6 | C | 13967 | T |
| SSPE 1 | 0 | Genome-1 | cluster 6 | C | 13967 | T |
| Cerebellum | 0.1177 | Genome-1 | cluster 6 | C | 13967 | T |
| Cerebellum Nucleus | 0.0263 | Genome-1 | cluster 6 | C | 13967 | T |
| Frontal Cortex 2 | 0 | Genome-1 | cluster 6 | C | 13967 | T |
| Internal Capsule | 0 | Genome-1 | cluster 6 | C | 13967 | T |
| Frontal Cortex 3 | 0 | Genome-1 | cluster 6 | C | 13967 | T |
| Hippocampus | 0 | Genome-1 | cluster 6 | C | 13967 | T |
| Temporal Lobe | 0 | Genome-1 | cluster 1a | A | 5455 | G |
| Parietal Lobe | 0 | Genome-1 | cluster 1a | A | 5455 | G |
| Upper Brain Stem | 0 | Genome-1 | cluster 1a | A | 5455 | G |
| Midbrain | 0 | Genome-1 | cluster 1a | A | 5455 | G |
| Frontal Cortex 1 | 0.0238 | Genome-1 | cluster 1a | A | 5455 | G |
| SSPE 2 | 0.3862 | Genome-1 | cluster 1a | A | 5455 | G |
| Brain Stem | 0 | Genome-1 | cluster 1a | A | 5455 | G |
| Occipital Lobe | 0 | Genome-1 | cluster 1a | A | 5455 | G |
| SSPE 1 | 0.0835 | Genome-1 | cluster 1a | A | 5455 | G |
| Cerebellum | 0 | Genome-1 | cluster 1a | A | 5455 | G |
| Cerebellum Nucleus | 0 | Genome-1 | cluster 1a | A | 5455 | G |
| Frontal Cortex 2 | 0 | Genome-1 | cluster 1a | A | 5455 | G |
| Internal Capsule | 0 | Genome-1 | cluster 1a | A | 5455 | G |
| Frontal Cortex 3 | 0.0238 | Genome-1 | cluster 1a | A | 5455 | G |
| Hippocampus | 0 | Genome-1 | cluster 1a | A | 5455 | G |
| Temporal Lobe | 0 | Genome-1 | cluster 1a | T | 7073 | C |
| Parietal Lobe | 0 | Genome-1 | cluster 1a | T | 7073 | C |
| Upper Brain Stem | 0 | Genome-1 | cluster 1a | T | 7073 | C |
| Midbrain | 0 | Genome-1 | cluster 1a | T | 7073 | C |
| Frontal Cortex 1 | 0.1198 | Genome-1 | cluster 1a | T | 7073 | C |
| SSPE 2 | 0.4049 | Genome-1 | cluster 1a | T | 7073 | C |
| Brain Stem | 0.0218 | Genome-1 | cluster 1a | T | 7073 | C |
| Occipital Lobe | 0.0243 | Genome-1 | cluster 1a | T | 7073 | C |
| SSPE 1 | 0.0922 | Genome-1 | cluster 1a | T | 7073 | C |
| Cerebellum | 0 | Genome-1 | cluster 1a | T | 7073 | C |
| Cerebellum Nucleus | 0 | Genome-1 | cluster 1a | T | 7073 | C |
| Frontal Cortex 2 | 0 | Genome-1 | cluster 1a | T | 7073 | C |
| Internal Capsule | 0 | Genome-1 | cluster 1a | T | 7073 | C |
| Frontal Cortex 3 | 0.1153 | Genome-1 | cluster 1a | T | 7073 | C |
| Hippocampus | 0 | Genome-1 | cluster 1a | T | 7073 | C |
| Temporal Lobe | 0 | Genome-1 | cluster 1a | T | 7093 | C |
| Parietal Lobe | 0 | Genome-1 | cluster 1a | T | 7093 | C |
| Upper Brain Stem | 0 | Genome-1 | cluster 1a | T | 7093 | C |
| Midbrain | 0 | Genome-1 | cluster 1a | T | 7093 | C |
| Frontal Cortex 1 | 0.0409 | Genome-1 | cluster 1a | T | 7093 | C |
| SSPE 2 | 0.4051 | Genome-1 | cluster 1a | T | 7093 | C |
| Brain Stem | 0 | Genome-1 | cluster 1a | T | 7093 | C |
| Occipital Lobe | 0 | Genome-1 | cluster 1a | T | 7093 | C |
| SSPE 1 | 0.0872 | Genome-1 | cluster 1a | T | 7093 | C |
| Cerebellum | 0 | Genome-1 | cluster 1a | T | 7093 | C |
| Cerebellum Nucleus | 0 | Genome-1 | cluster 1a | T | 7093 | C |
| Frontal Cortex 2 | 0 | Genome-1 | cluster 1a | T | 7093 | C |
| Internal Capsule | 0 | Genome-1 | cluster 1a | T | 7093 | C |
| Frontal Cortex 3 | 0.0422 | Genome-1 | cluster 1a | T | 7093 | C |
| Hippocampus | 0 | Genome-1 | cluster 1a | T | 7093 | C |
| Temporal Lobe | 0 | Genome-1 | cluster 1 | G | 9383 | A |
| Parietal Lobe | 0 | Genome-1 | cluster 1 | G | 9383 | A |
| Upper Brain Stem | 0 | Genome-1 | cluster 1 | G | 9383 | A |
| Midbrain | 0 | Genome-1 | cluster 1 | G | 9383 | A |
| Frontal Cortex 1 | 0.1007 | Genome-1 | cluster 1 | G | 9383 | A |
| SSPE 2 | 0.5877 | Genome-1 | cluster 1 | G | 9383 | A |
| Brain Stem | 0 | Genome-1 | cluster 1 | G | 9383 | A |
| Occipital Lobe | 0 | Genome-1 | cluster 1 | G | 9383 | A |
| SSPE 1 | 0.4747 | Genome-1 | cluster 1 | G | 9383 | A |
| Cerebellum | 0 | Genome-1 | cluster 1 | G | 9383 | A |
| Cerebellum Nucleus | 0.020663 | Genome-1 | cluster 1 | G | 9383 | A |
| Frontal Cortex 2 | 0 | Genome-1 | cluster 1 | G | 9383 | A |
| Internal Capsule | 0 | Genome-1 | cluster 1 | G | 9383 | A |
| Frontal Cortex 3 | 0.109 | Genome-1 | cluster 1 | G | 9383 | A |
| Hippocampus | 0 | Genome-1 | cluster 1 | G | 9383 | A |
| Temporal Lobe | 0 | Genome-1 | cluster 1 | G | 14246 | A |
| Parietal Lobe | 0 | Genome-1 | cluster 1 | G | 14246 | A |
| Upper Brain Stem | 0 | Genome-1 | cluster 1 | G | 14246 | A |
| Midbrain | 0 | Genome-1 | cluster 1 | G | 14246 | A |
| Frontal Cortex 1 | 0.0979 | Genome-1 | cluster 1 | G | 14246 | A |
| SSPE 2 | 0.574 | Genome-1 | cluster 1 | G | 14246 | A |
| Brain Stem | 0 | Genome-1 | cluster 1 | G | 14246 | A |
| Occipital Lobe | 0 | Genome-1 | cluster 1 | G | 14246 | A |
| SSPE 1 | 0.4623 | Genome-1 | cluster 1 | G | 14246 | A |
| Cerebellum | 0 | Genome-1 | cluster 1 | G | 14246 | A |
| Cerebellum Nucleus | 0 | Genome-1 | cluster 1 | G | 14246 | A |
| Frontal Cortex 2 | 0 | Genome-1 | cluster 1 | G | 14246 | A |
| Internal Capsule | 0 | Genome-1 | cluster 1 | G | 14246 | A |
| Frontal Cortex 3 | 0.0984 | Genome-1 | cluster 1 | G | 14246 | A |
| Hippocampus | 0 | Genome-1 | cluster 1 | G | 14246 | A |
| Temporal Lobe | 0 | Genome-1 | Genome-FC2 | T | 949 | A |
| Parietal Lobe | 0 | Genome-1 | Genome-FC2 | T | 949 | A |
| Upper Brain Stem | 0 | Genome-1 | Genome-FC2 | T | 949 | A |
| Midbrain | 0 | Genome-1 | Genome-FC2 | T | 949 | A |
| Frontal Cortex 1 | 0 | Genome-1 | Genome-FC2 | T | 949 | A |
| SSPE 2 | 0 | Genome-1 | Genome-FC2 | T | 949 | A |
| Brain Stem | 0 | Genome-1 | Genome-FC2 | T | 949 | A |
| Occipital Lobe | 0.020136 | Genome-1 | Genome-FC2 | T | 949 | A |
| SSPE 1 | 0 | Genome-1 | Genome-FC2 | T | 949 | A |
| Cerebellum | 0 | Genome-1 | Genome-FC2 | T | 949 | A |
| Cerebellum Nucleus | 0 | Genome-1 | Genome-FC2 | T | 949 | A |
| Frontal Cortex 2 | 0.5092 | Genome-1 | Genome-FC2 | T | 949 | A |
| Internal Capsule | 0 | Genome-1 | Genome-FC2 | T | 949 | A |
| Frontal Cortex 3 | 0 | Genome-1 | Genome-FC2 | T | 949 | A |
| Hippocampus | 0 | Genome-1 | Genome-FC2 | T | 949 | A |
| Temporal Lobe | 0 | Genome-1 | Genome-FC2 | C | 3740 | T |
| Parietal Lobe | 0 | Genome-1 | Genome-FC2 | C | 3740 | T |
| Upper Brain Stem | 0 | Genome-1 | Genome-FC2 | C | 3740 | T |
| Midbrain | 0 | Genome-1 | Genome-FC2 | C | 3740 | T |
| Frontal Cortex 1 | 0 | Genome-1 | Genome-FC2 | C | 3740 | T |
| SSPE 2 | 0 | Genome-1 | Genome-FC2 | C | 3740 | T |
| Brain Stem | 0 | Genome-1 | Genome-FC2 | C | 3740 | T |
| Occipital Lobe | 0.020656 | Genome-1 | Genome-FC2 | C | 3740 | T |
| SSPE 1 | 0 | Genome-1 | Genome-FC2 | C | 3740 | T |
| Cerebellum | 0 | Genome-1 | Genome-FC2 | C | 3740 | T |
| Cerebellum Nucleus | 0 | Genome-1 | Genome-FC2 | C | 3740 | T |
| Frontal Cortex 2 | 0.4931 | Genome-1 | Genome-FC2 | C | 3740 | T |
| Internal Capsule | 0 | Genome-1 | Genome-FC2 | C | 3740 | T |
| Frontal Cortex 3 | 0 | Genome-1 | Genome-FC2 | C | 3740 | T |
| Hippocampus | 0 | Genome-1 | Genome-FC2 | C | 3740 | T |
| Temporal Lobe | 0 | Genome-1 | Genome-FC2 | A | 4249 | G |
| Parietal Lobe | 0 | Genome-1 | Genome-FC2 | A | 4249 | G |
| Upper Brain Stem | 0 | Genome-1 | Genome-FC2 | A | 4249 | G |
| Midbrain | 0 | Genome-1 | Genome-FC2 | A | 4249 | G |
| Frontal Cortex 1 | 0 | Genome-1 | Genome-FC2 | A | 4249 | G |
| SSPE 2 | 0 | Genome-1 | Genome-FC2 | A | 4249 | G |
| Brain Stem | 0 | Genome-1 | Genome-FC2 | A | 4249 | G |
| Occipital Lobe | 0 | Genome-1 | Genome-FC2 | A | 4249 | G |
| SSPE 1 | 0 | Genome-1 | Genome-FC2 | A | 4249 | G |
| Cerebellum | 0 | Genome-1 | Genome-FC2 | A | 4249 | G |
| Cerebellum Nucleus | 0 | Genome-1 | Genome-FC2 | A | 4249 | G |
| Frontal Cortex 2 | 0.4774 | Genome-1 | Genome-FC2 | A | 4249 | G |
| Internal Capsule | 0 | Genome-1 | Genome-FC2 | A | 4249 | G |
| Frontal Cortex 3 | 0 | Genome-1 | Genome-FC2 | A | 4249 | G |
| Hippocampus | 0 | Genome-1 | Genome-FC2 | A | 4249 | G |
| Temporal Lobe | 0 | Genome-1 | Genome-FC2 | T | 5312 | C |
| Parietal Lobe | 0 | Genome-1 | Genome-FC2 | T | 5312 | C |
| Upper Brain Stem | 0 | Genome-1 | Genome-FC2 | T | 5312 | C |
| Midbrain | 0 | Genome-1 | Genome-FC2 | T | 5312 | C |
| Frontal Cortex 1 | 0 | Genome-1 | Genome-FC2 | T | 5312 | C |
| SSPE 2 | 0 | Genome-1 | Genome-FC2 | T | 5312 | C |
| Brain Stem | 0 | Genome-1 | Genome-FC2 | T | 5312 | C |
| Occipital Lobe | 0 | Genome-1 | Genome-FC2 | T | 5312 | C |
| SSPE 1 | 0 | Genome-1 | Genome-FC2 | T | 5312 | C |
| Cerebellum | 0 | Genome-1 | Genome-FC2 | T | 5312 | C |
| Cerebellum Nucleus | 0 | Genome-1 | Genome-FC2 | T | 5312 | C |
| Frontal Cortex 2 | 0.3745 | Genome-1 | Genome-FC2 | T | 5312 | C |
| Internal Capsule | 0 | Genome-1 | Genome-FC2 | T | 5312 | C |
| Frontal Cortex 3 | 0 | Genome-1 | Genome-FC2 | T | 5312 | C |
| Hippocampus | 0 | Genome-1 | Genome-FC2 | T | 5312 | C |
| Temporal Lobe | 0 | Genome-1 | Genome-FC2 | C | 6810 | T |
| Parietal Lobe | 0 | Genome-1 | Genome-FC2 | C | 6810 | T |
| Upper Brain Stem | 0 | Genome-1 | Genome-FC2 | C | 6810 | T |
| Midbrain | 0 | Genome-1 | Genome-FC2 | C | 6810 | T |
| Frontal Cortex 1 | 0 | Genome-1 | Genome-FC2 | C | 6810 | T |
| SSPE 2 | 0 | Genome-1 | Genome-FC2 | C | 6810 | T |
| Brain Stem | 0 | Genome-1 | Genome-FC2 | C | 6810 | T |
| Occipital Lobe | 0 | Genome-1 | Genome-FC2 | C | 6810 | T |
| SSPE 1 | 0 | Genome-1 | Genome-FC2 | C | 6810 | T |
| Cerebellum | 0 | Genome-1 | Genome-FC2 | C | 6810 | T |
| Cerebellum Nucleus | 0 | Genome-1 | Genome-FC2 | C | 6810 | T |
| Frontal Cortex 2 | 0.3894 | Genome-1 | Genome-FC2 | C | 6810 | T |
| Internal Capsule | 0 | Genome-1 | Genome-FC2 | C | 6810 | T |
| Frontal Cortex 3 | 0 | Genome-1 | Genome-FC2 | C | 6810 | T |
| Hippocampus | 0 | Genome-1 | Genome-FC2 | C | 6810 | T |
| Temporal Lobe | 0 | Genome-1 | Genome-FC2 | G | 7825 | A |
| Parietal Lobe | 0 | Genome-1 | Genome-FC2 | G | 7825 | A |
| Upper Brain Stem | 0 | Genome-1 | Genome-FC2 | G | 7825 | A |
| Midbrain | 0 | Genome-1 | Genome-FC2 | G | 7825 | A |
| Frontal Cortex 1 | 0 | Genome-1 | Genome-FC2 | G | 7825 | A |
| SSPE 2 | 0 | Genome-1 | Genome-FC2 | G | 7825 | A |
| Brain Stem | 0 | Genome-1 | Genome-FC2 | G | 7825 | A |
| Occipital Lobe | 0 | Genome-1 | Genome-FC2 | G | 7825 | A |
| SSPE 1 | 0 | Genome-1 | Genome-FC2 | G | 7825 | A |
| Cerebellum | 0 | Genome-1 | Genome-FC2 | G | 7825 | A |
| Cerebellum Nucleus | 0 | Genome-1 | Genome-FC2 | G | 7825 | A |
| Frontal Cortex 2 | 0.4002 | Genome-1 | Genome-FC2 | G | 7825 | A |
| Internal Capsule | 0 | Genome-1 | Genome-FC2 | G | 7825 | A |
| Frontal Cortex 3 | 0 | Genome-1 | Genome-FC2 | G | 7825 | A |
| Hippocampus | 0 | Genome-1 | Genome-FC2 | G | 7825 | A |
