# Supplementary figures and images for: Brain tropism acquisition: The spatial dynamics and evolution of a measles virus collective infectious unit that drove lethal subacute sclerosing panencephalitis
Source: PLoS Pathog. 2023 Dec 21;19(12):e1011817. doi: 10.1371/journal.ppat.1011817 (PMC10735034; doi:10.1371/journal.ppat.1011817)

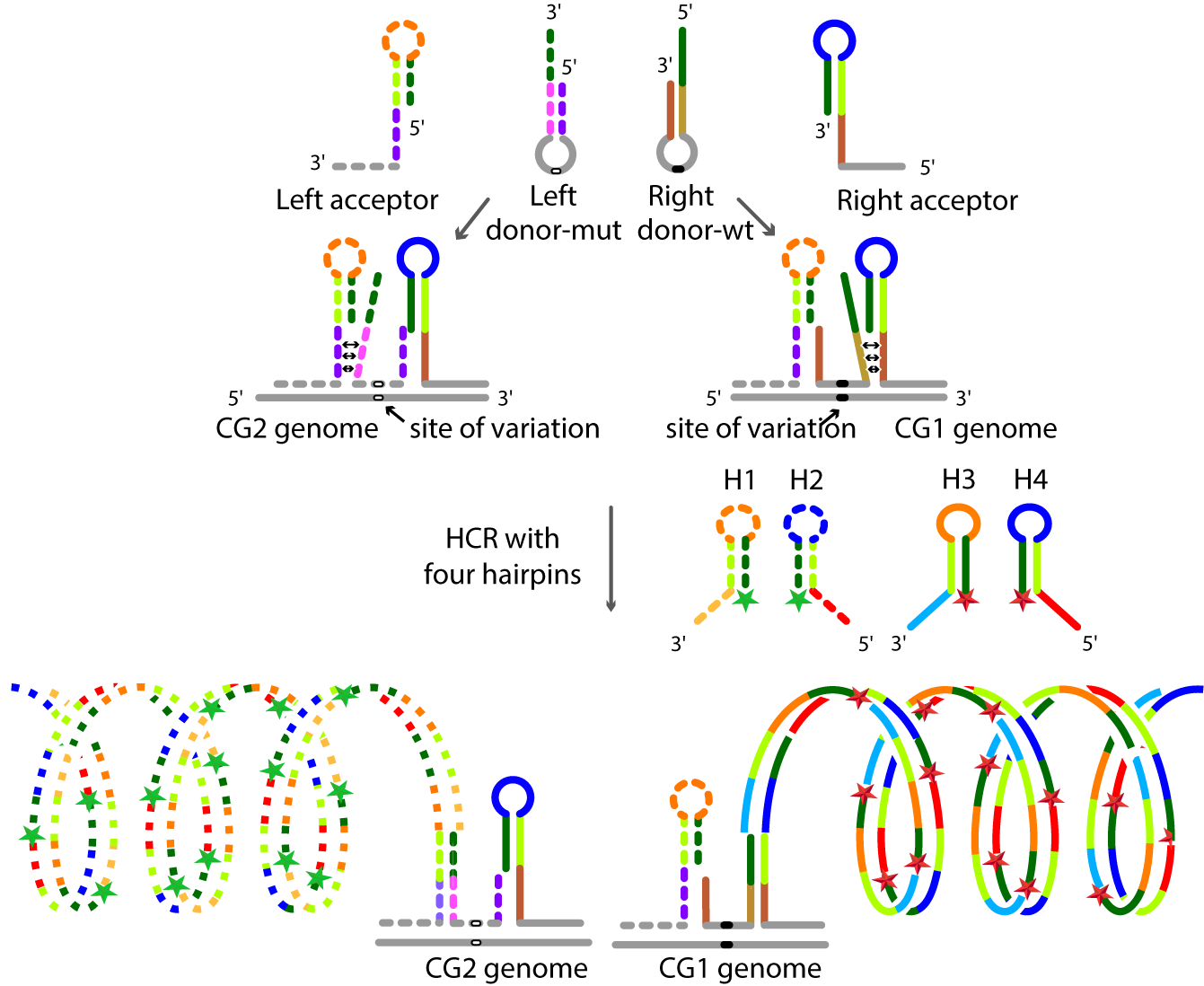

Supplement: S1 Fig — (Top) Probes used simultaneously to discriminate CG1 from CG2. The grey regions of the probes bind to the targets. The right and left acceptor probes bind on either side of the region encompassing the SNV. Only one of the donor probes can bind to the SNV region depending on the SNV that is present in the genome. To improve signal strength, we targeted a total of 10 SNVs using four sets of probes for each genome, where all SNVs in the CG1 gave rise to red signals and all SNVs in the CG2 gave rise to green signals. Sequence of grey regions for CG1 probes for SNVs 3907, 3908 and 3912: 5’atatGaacGGcacggaac3’ (SNV are capitalized); for SNV 3139 and 3140: 5’gctcaccTTtttcccgat3’; for SNVs 4087, 4088 and 4090: 5’gtggaccGtGGatgttgc3’; and for SNVs 4309 and 4310: 5’accgattGGggtcttc3’. Sequence of grey regions for CG2 probes for SNVs 3907, 3908 and 3912: 5’atatAaacAAcacggaac3’; for SNVs 3139 and 3140: 5’gctcaccCCtttcccgat3’; for SNVs 4087, 4088 and 4090: 5’gtggaccAtAAatgttgc3’ and for SNVs 4309 and 4310: 5’accgattAAggtctt3’. (Center and bottom) The binding of the left donor-mut to the CG2 target sequence initiates a strand displacement reaction in the left acceptor that leads to generation of a green HCR signal using Cy3-labeled HCR hairpins H1 and H2. The binding of the right donor-wt to the CG1 genome target sequence initiates a strand-displacement reaction in the right acceptor that leads to generation of a red HCR signal using Cy5-labeled HCR hairpins H3 and H4. (TIF) [file ppat.1011817.s003.tif]

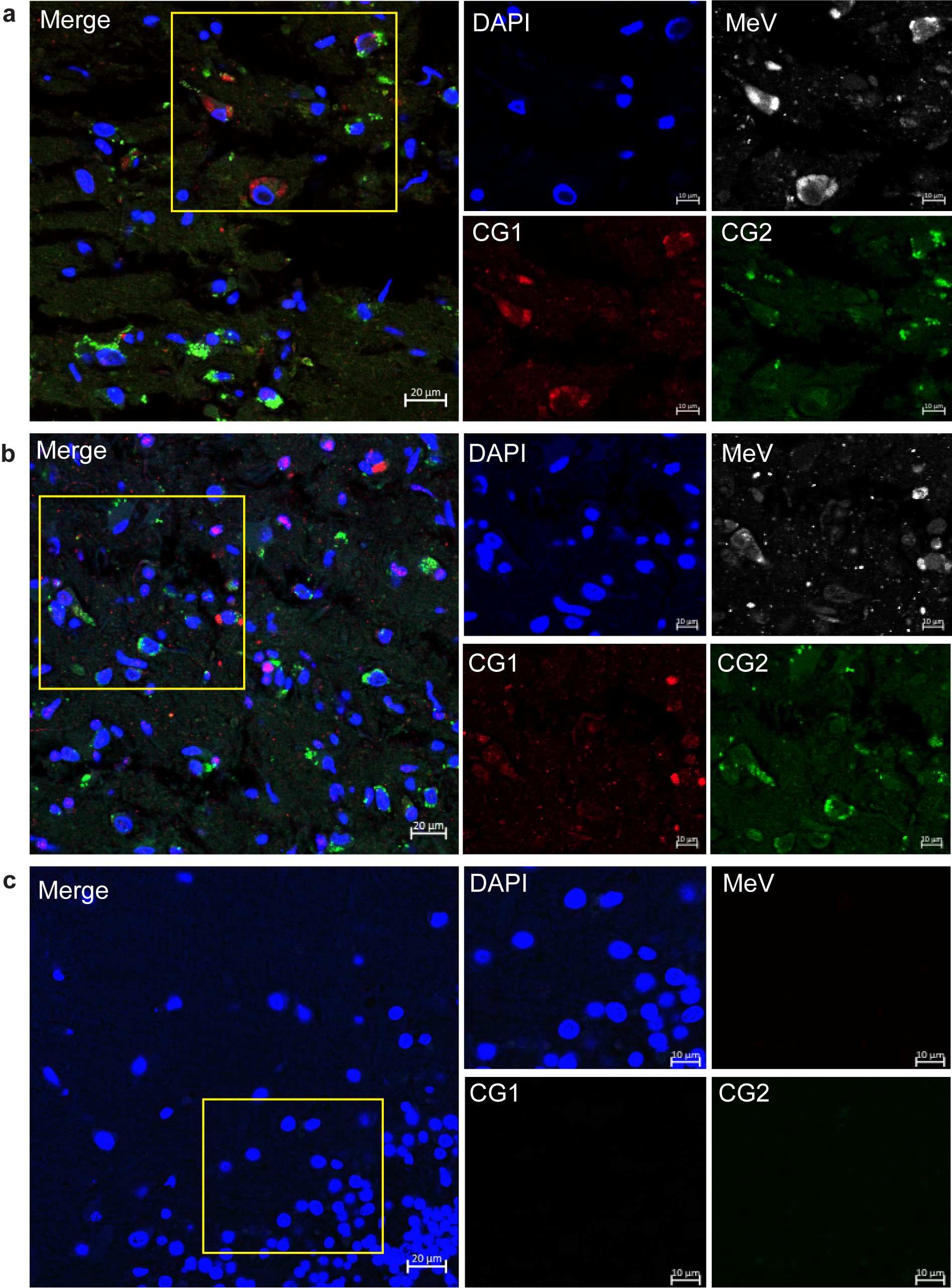

Supplement: S2 Fig — (A-C) Confocal images showing nuclei in blue, MeV M mRNA in grey, CG1 in red and CG2 in green. (A) SSPE temporal lobe, (B) SSPE occipital lobe and (C) healthy human cerebral cortex. Individual channels for the yellow boxed areas are shown in the right panels. (TIF) [file ppat.1011817.s004.tif]

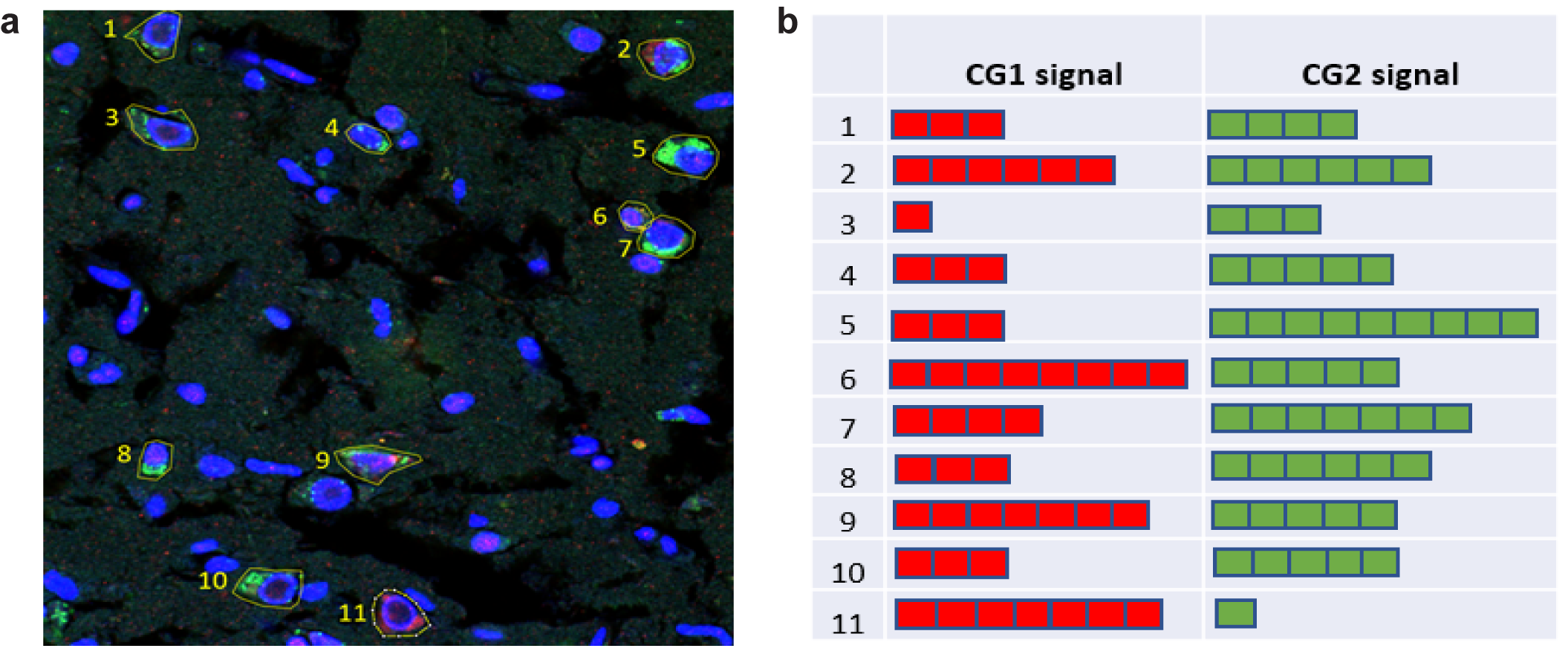

Supplement: S3 Fig — (A) Eleven infected cells marked for ImageJ analysis; yellow outlines identify the areas analyzed. CG1 signals are in red, CG2 signals in green. Nuclei are counterstained with DAPI (blue). (B) Table reporting intensity levels of the CG1 (red squares) and CG2 (green squares) signals in the 11 cells marked in panel (A). Each square represents 5 intensity units. For calculating the signal intensity of CG1 and CG2 in each cell, we divided either CG1 or CG2 signal by the total signal from both probes. As an example, for cell number 1, CG1 signal intensity is 15 units and CG2 is 20 units so the percentage of CG1 signal in that cell will be 15/35*100 i.e., 42.8% and the percentage of CG2 will be 57.2%. (TIF) [file ppat.1011817.s005.tif]

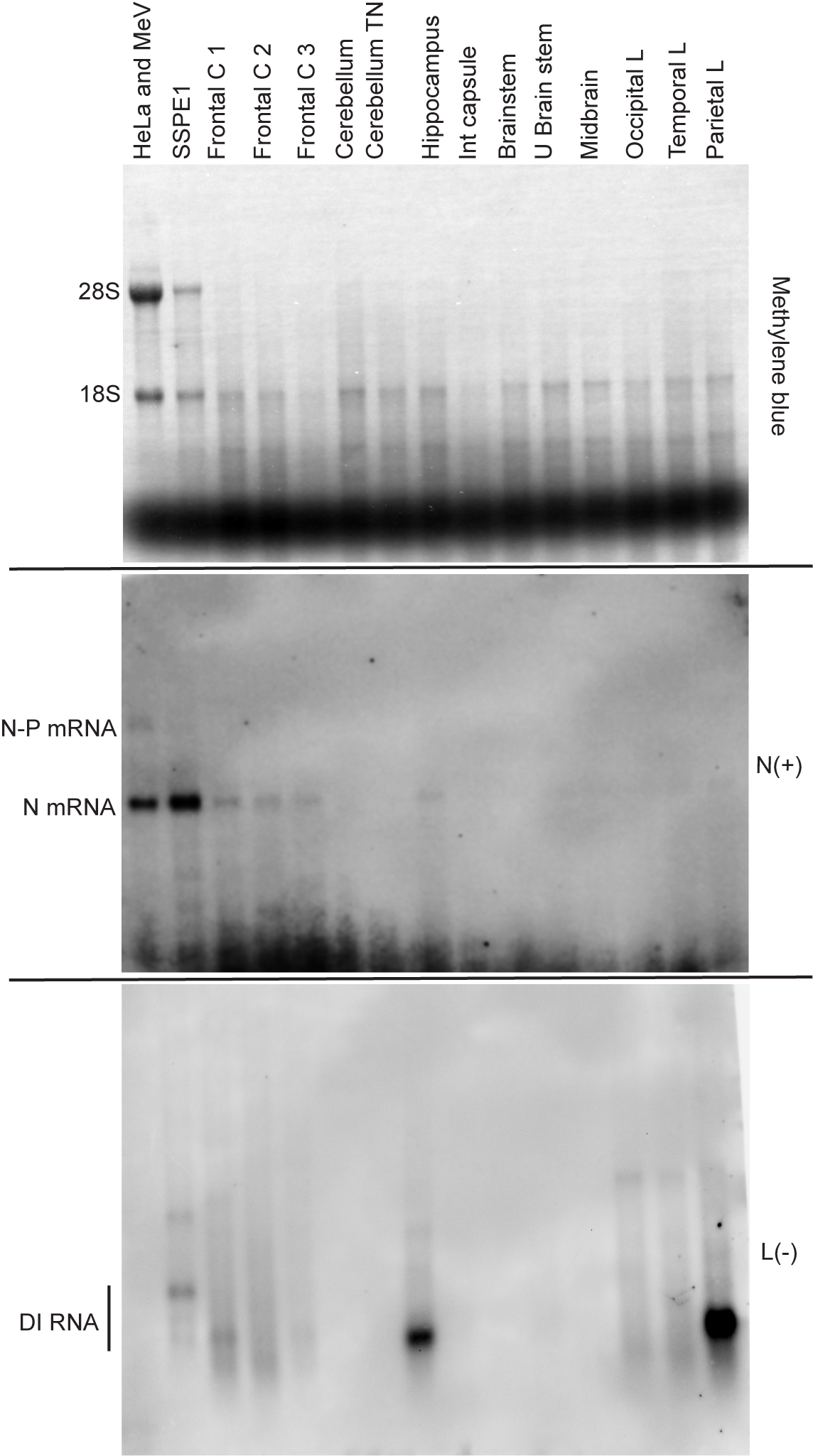

Supplement: S4 Fig — Specimens analyzed are listed above each lane. C, cortex; TN, towards nucleus, L, lobe. Top panel: methylene blue stained RNA gel. The ribosomal 28S and 18S RNA positions are indicated. Middle panel: Northern blot probed with N(+) probe detecting positive strand RNA. The N and N-P mRNAs are indicated. Bottom panel: Northern blot probed with L(-) probe detecting genomic RNA. DI RNA: short defective RNAs. (TIF) [file ppat.1011817.s006.tif]

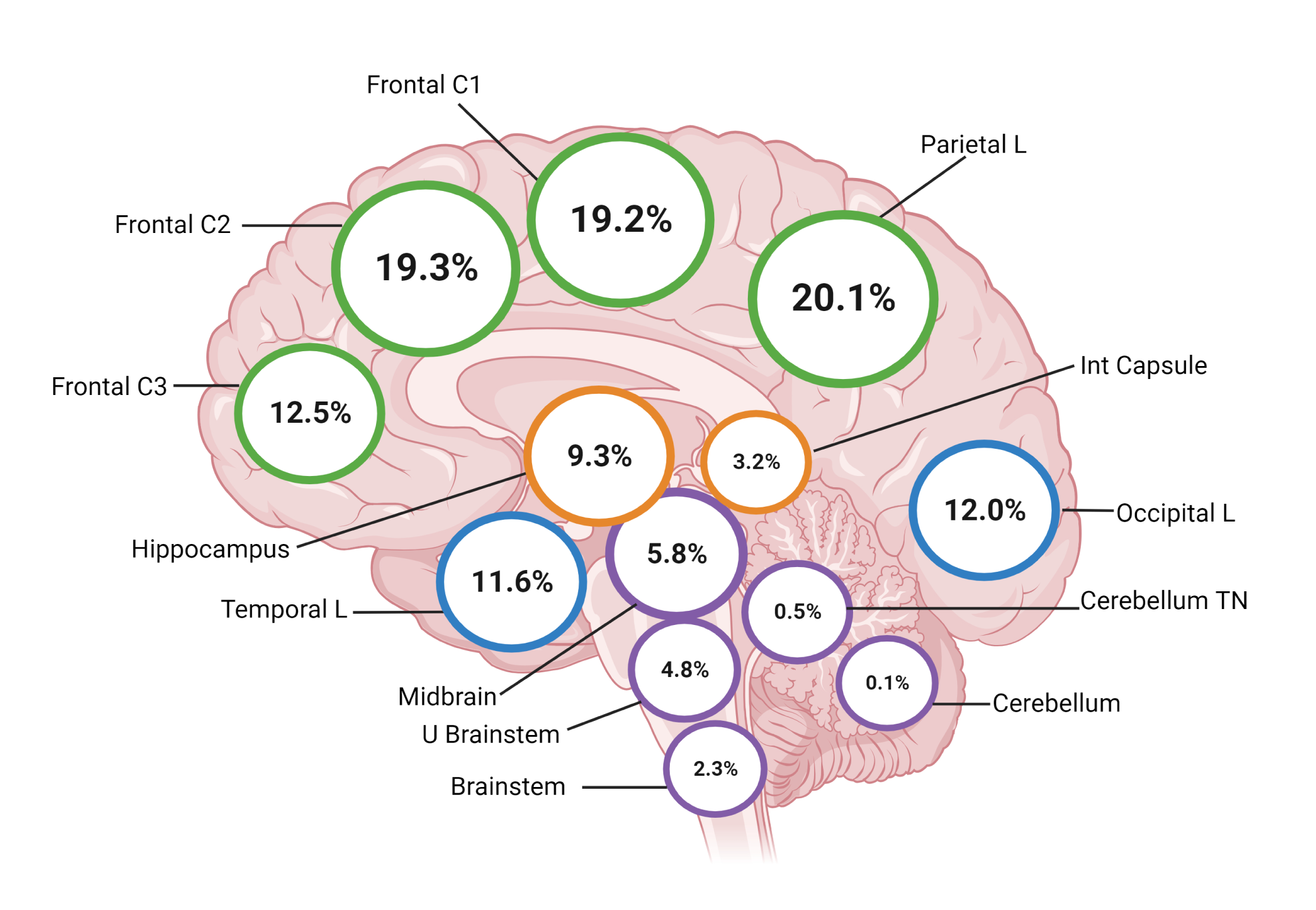

Supplement: S5 Fig — Large, intermediate, or small circles represent specimens with >13%, 5–13% or less than 5% MeV reads, respectively. Anatomically closer brain regions are indicated with the same color circle outlines. L = lobe, C = cortex, U = Upper, Int = Internal and TN = Towards nucleus. Image was generated in BioRender. (TIF) [file ppat.1011817.s007.tif]

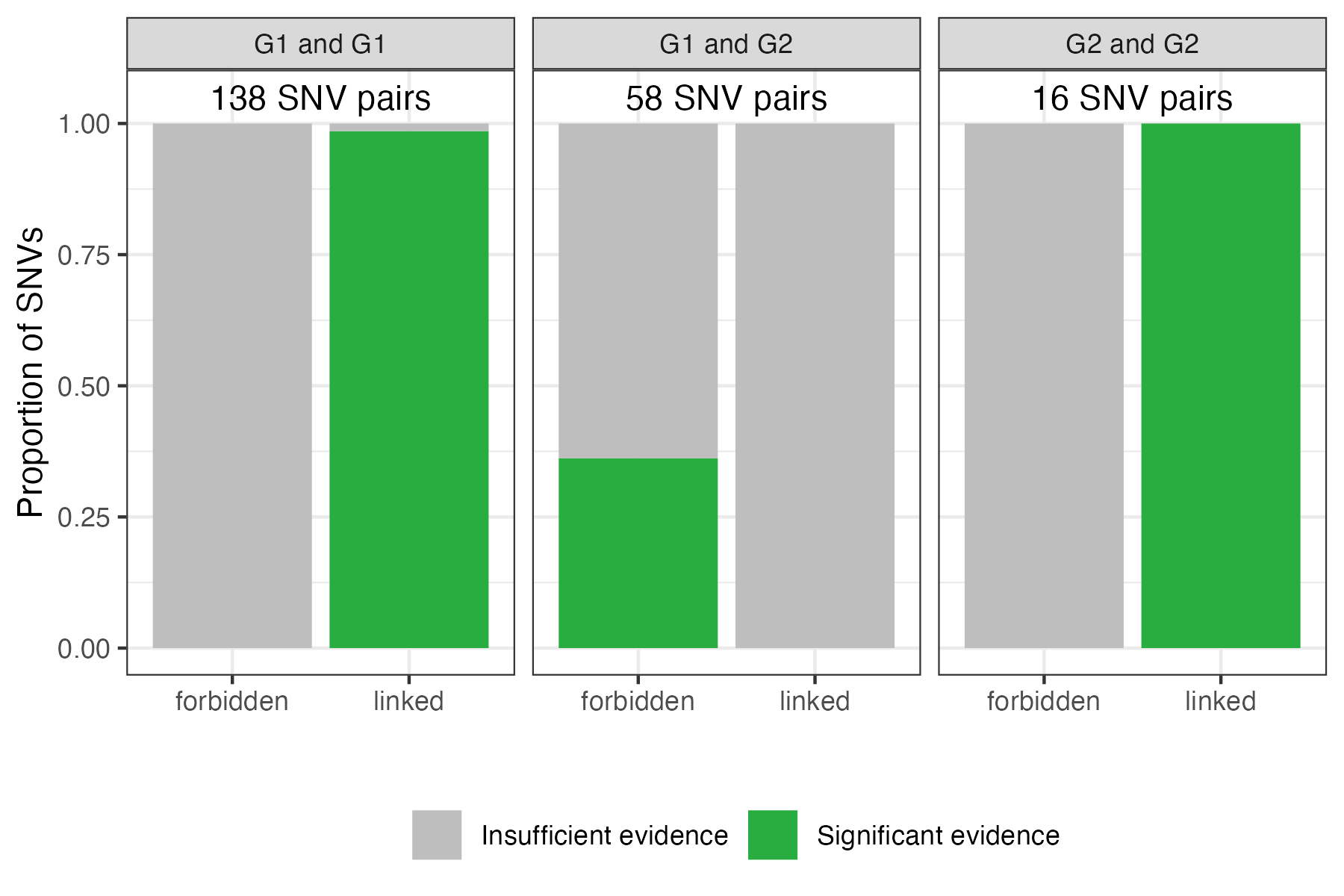

Supplement: S6 Fig — Y-axis: proportion of SNV pairs with bridging reads showing a statistically significant effect or not for a given test; x-axis: statistical test determining whether a SNV pair is linked (part of the same haplotype) or forbidden (mutually exclusive). Green indicates statistically significant evidence, whereas gray represents the lack of evidence. The absence of evidence for linkage does not imply that a pair of SNVs is forbidden. The converse is also true, that the absence of evidence for two SNVs being forbidden does not mean that they are linked. 138 G1/G1 pairs were tested, 16 G1/G2 pairs were tested, and 58 G2/G2 pairs were tested. (TIFF) [file ppat.1011817.s008.tiff]

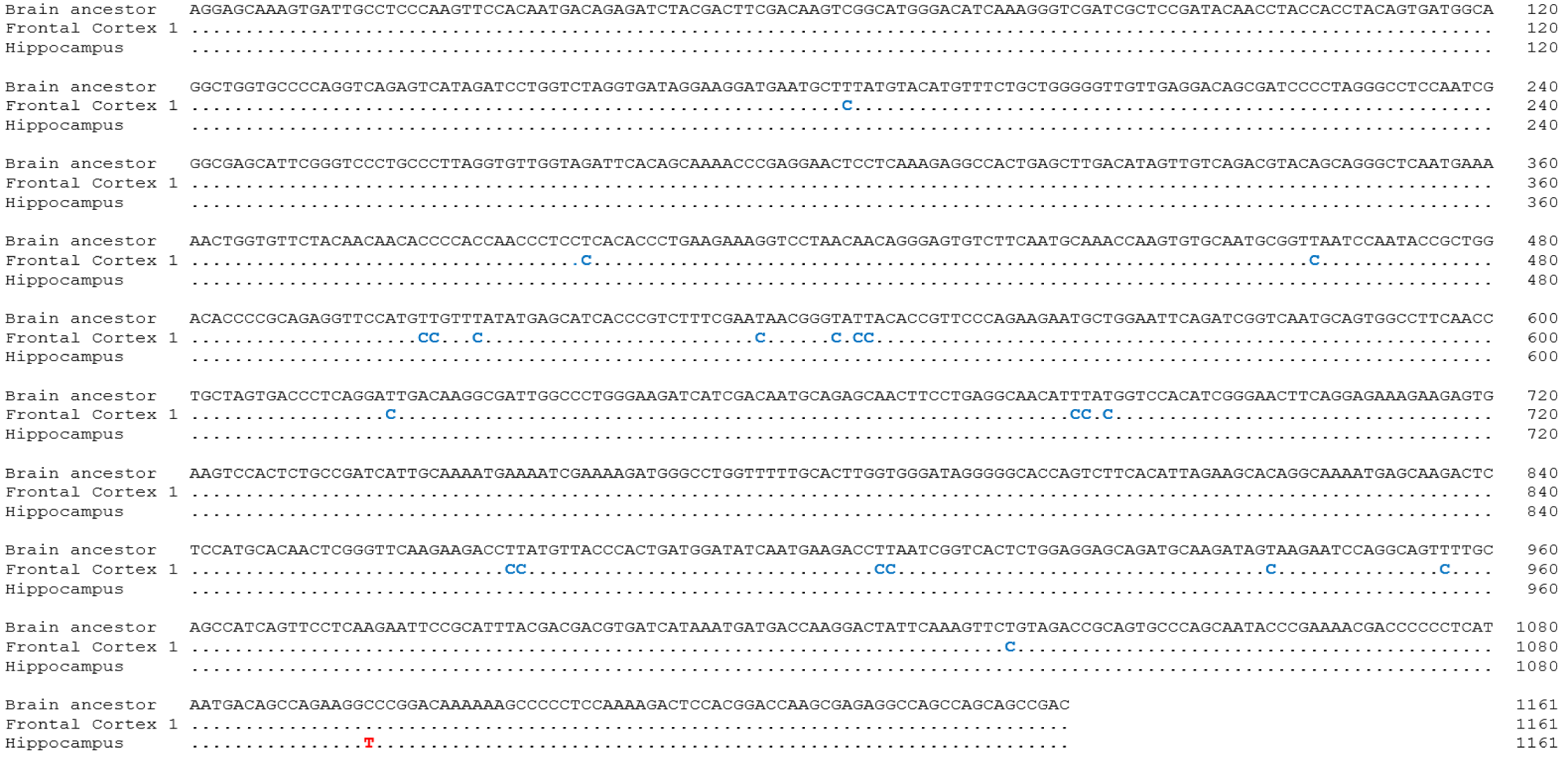

Supplement: S7 Fig — RNA from Frontal Cortex 1 (G1 high) and Hippocampus (G2 high) was used for cDNA synthesis using the template switching RT enzyme mix (New England Biolabs) with an N6 TS modified random primer [81]. A single library was generated (samples were barcoded and pooled) using the native barcoding SQK-NBD-114-96 Q20+ sequencing kit (Oxford Nanopore Technologies, ONT). The ONT library was sequenced in a single sequencing run using the high-accuracy base-calling model with a minimum Q score of 10 set on an ONT GridION device using one MinION Flow Cell R10.4.1. Using default parameters for all software, the corrected reads obtained (Frontal Cortex 1: 8,585; Hippocampus: 7,380) were aligned against the M gene using Muscle 3.8.425 in Geneious Prime 2021.1.1. The M-gene mapped reads (Frontal Cortex 1: 477; Hippocampus: 352), were further selected based on coverage of >95% of the M protein coding sequence. The longest reads, namely 57a2eac3-d91e-465f-b823-5cc9c757327f (Frontal Cortex 1) and 25e1cdcf-007f-4260-843b-abd1f4230a30 (Hippocampus) are shown. These reads correspond to the dominant haplotypes in each specimen. Blue SNVs are G1 and red SNVs are G2. (TIF) [file ppat.1011817.s009.tif]

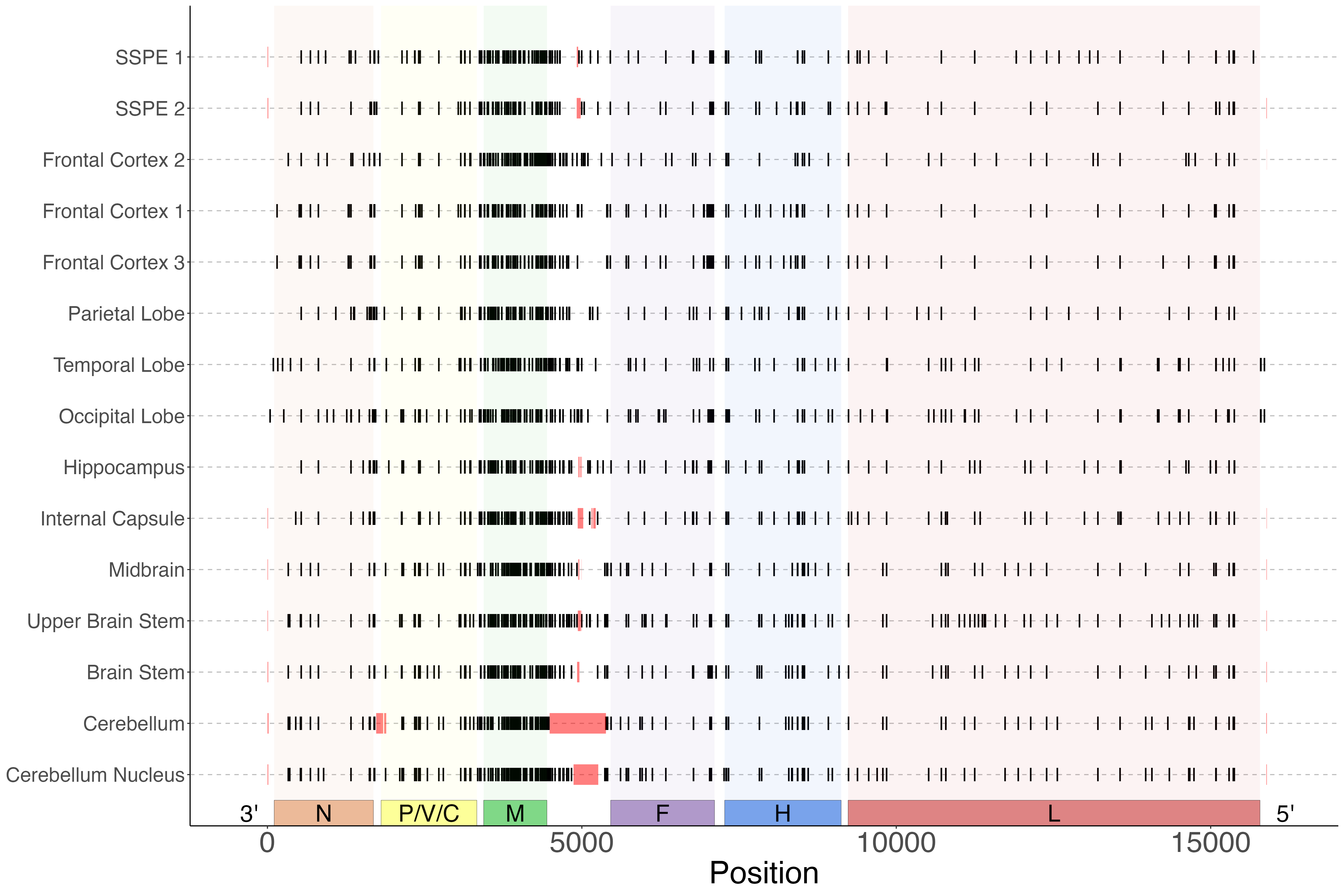

Supplement: S8 Fig — Mutations were called relative to BA. Y-axis: specimen names; x-axis: position of each mutation. Pink blocks show areas where the read depth was too low to confidently call variants. (TIFF) [file ppat.1011817.s010.tiff]

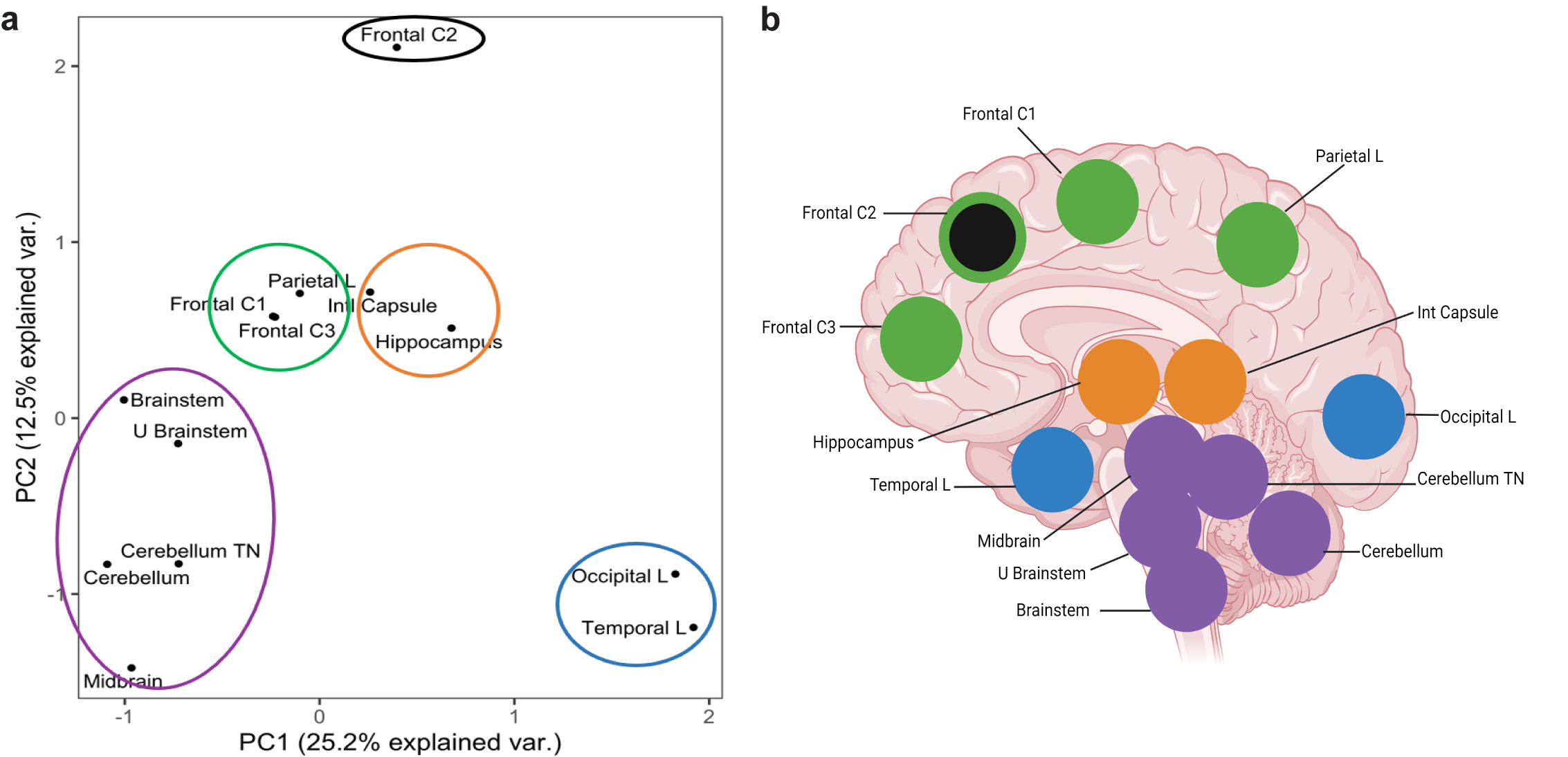

Supplement: S9 Fig — (A) Principal components PC1 (x-axis) and PC2 (y-axis) analysis of MeV genome populations. Five groups of genetically similar specimens are encircled by color-coded lines. (B) Brain drawing with superimposed circles of the same color for anatomically close locations. The center of Frontal cortex 2 specimen is indicated in black to mark that its PC analysis position does not reflect its anatomical position. C, cortex; L, lobe; U, upper; Int, internal; TN, towards nucleus. (B) was generated in BioRender. (TIF) [file ppat.1011817.s011.tif]

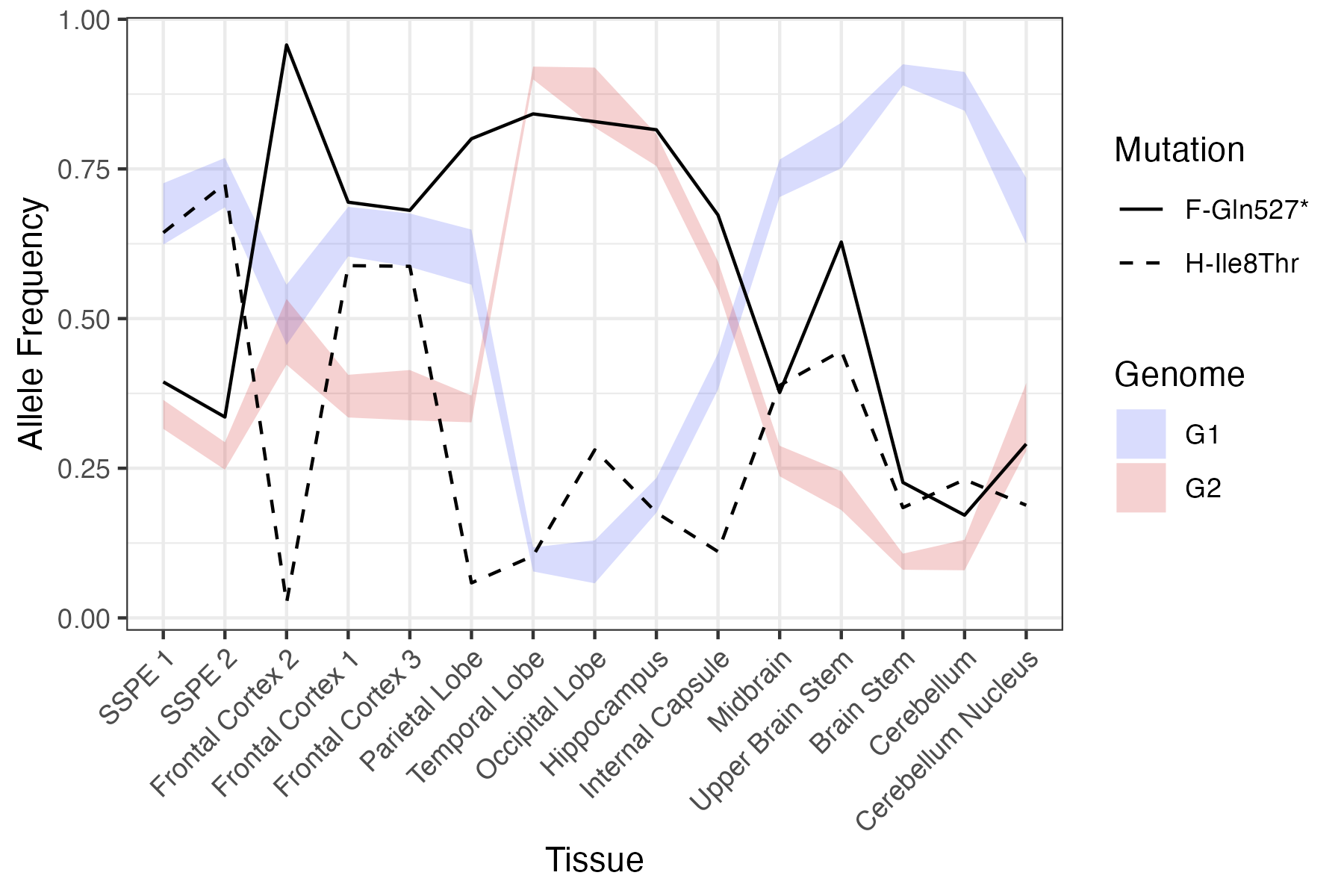

Supplement: S10 Fig — X-axis: brain specimens; y-axis; allele frequencies. The mean frequency of G1 mutations +/- the standard deviation in G1 frequency in each tissue is shown in blue. The same is shown in red for G2 mutations. The solid black line shows the frequencies of F-Q527* in each tissue and the black dashed line shows frequencies of H-I8T. (TIFF) [file ppat.1011817.s012.tiff]

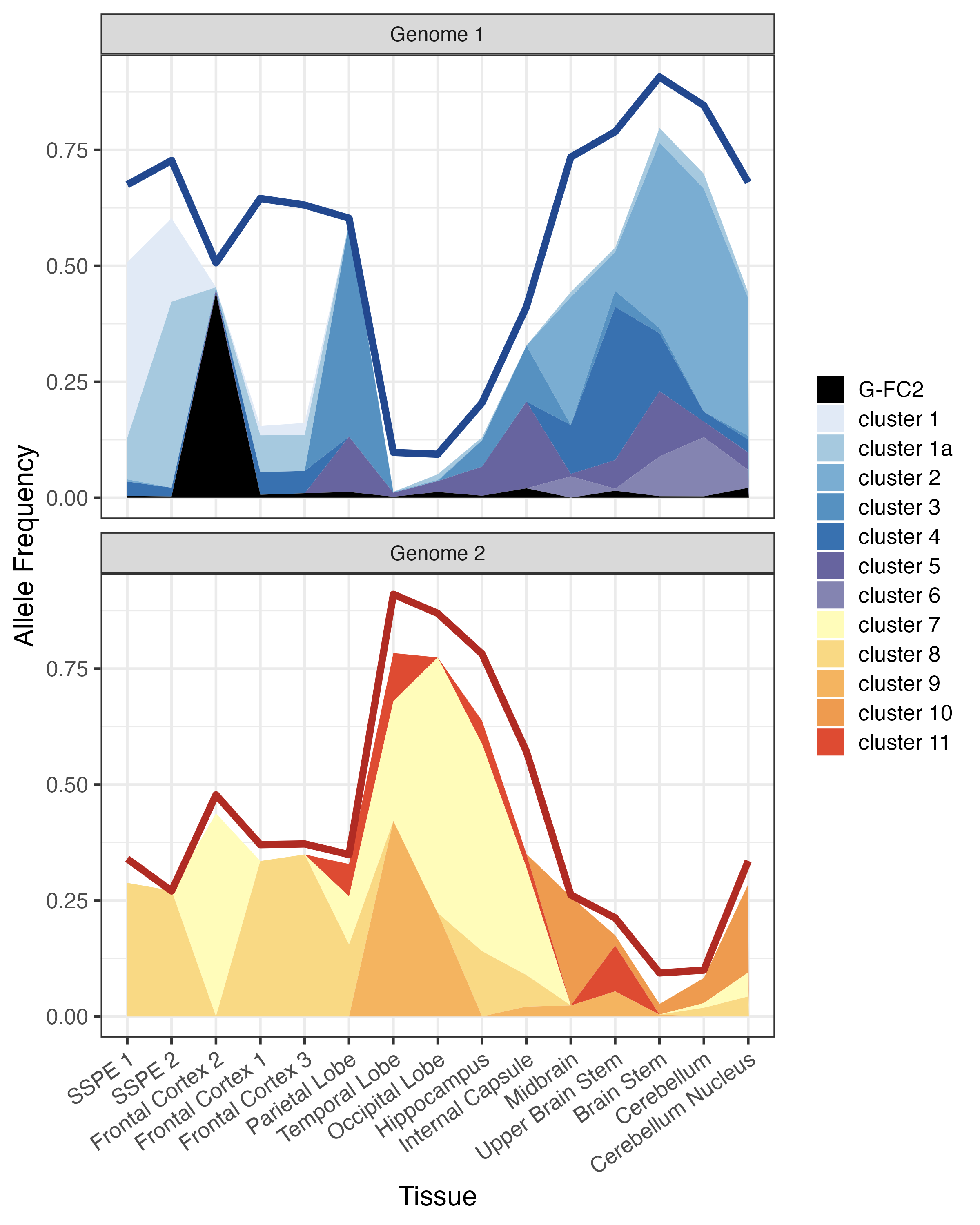

Supplement: S12 Fig — x-axis, brain specimens; y-axis, allele frequencies. Top panel: frequencies of G-01 (blue line) and its descendants (shaded areas color-coded according to the key on the right). Bottom panel: frequencies of G2 (red line) and its descendants (shaded areas color-coded according to the key on the right). (TIFF) [file ppat.1011817.s014.tiff]
