# Supplementary material for: Brain tropism acquisition: The spatial dynamics and evolution of a measles virus collective infectious unit that drove lethal subacute sclerosing panencephalitis
Source: PLoS Pathog. 2023 Dec 21;19(12):e1011817. doi: 10.1371/journal.ppat.1011817 (PMC10735034; doi:10.1371/journal.ppat.1011817)
Supplement: Attachment — Submitted filename: Rebuttal 231005.pdf [file ppat.1011817.s015.pdf]

Part I - Summary
Please use this section to discuss strengths/weaknesses of study, novelty/significance, general execution and scholarship.

Reviewer #1: In “Spatial dynamics and evolution of a measles virus genome collective that drove lethal human brain disease” Yousaf et al. use high-throughput metagenomic sequencing data to infer the dynamics of a measles virus infection in the brain of one individual. Overall, I found the study to be thoughtfully conducted and well-presented. Although this is just a single case study, and therefore impossible to generalize across SSPE cases, it is a unique dataset that offers several interesting insights into potential trajectories of chronic measles virus infections. However, I would like to see some methodological clarifications, some additional analysis to support the haplotype phasing and some tempering of the way conclusions are being presented.

Reviewer #2: In this study, the authors conducted a comprehensive analysis of MeV RNA from 15 distinct brain regions of an individual who sadly succumbed to SSPE. Utilizing deep sequencing and single-cell level techniques, they unveiled extensive replication occurring in most regions.

The researchers postulated that the initiation of brain spread originates in the frontal cortex. Moreover, they identified two distinct major subpopulations of the MeV genome across all 15 brain specimens. However, the proportions of these subpopulations varied among the different brain samples.

The study also discovered the presence of specific mutations affecting the cytoplasmic tails of the envelope proteins, namely F and hemagglutinin (H). Interestingly, the frequency of these mutations fluctuated across brain regions. The authors argue that the modulation of fusogenicity, crucial for brain spread, is controlled by the proportion of these mutations. This phenomenon may allow the virus to achieve optimal fusogenicity. While the study holds significant importance, certain aspects require better clarification and/or additional supporting data.

Reviewer #3: In a remarkable experimental tour de force the authors have used deep sequencing to analyze patterns of measles RNA in the entire brain of a patient who died of SSPE 20 years after the initial measles virus infection. The availability of the entire brain allowed characterization of viral genomes present in different parts of the brain and reconstruction of a plausible series of events that occurred after the initial infection, leading to widespread movement of viral genomes throughout the brain.

The findings provide important insights into the processes that drove lethal pan encephalitis and establish an unprecedented framework for the pathogenesis of SSPE that was not possible to construct from previous limited analyses. This description of SSPE will no doubt make its way into textbooks.

The conclusions are generally supported by the data although a certain level of speculation is necessary on the sequence of events, given that what happened in the previous 20 years of incubation had to be inferred. The superb analysis of deep sequencing data from different regions of the brain, combined with single cell RNA analysis provides unique insight into SSPE.

We thank the reviewers for carefully reading the manuscript and recognizing its importance. We have addressed all the specific points raised in parts II and III.

Part II – Major Issues: Key Experiments Required for Acceptance
Please use this section to detail the key new experiments or modifications of existing experiments that should be absolutely required to validate study conclusions.

Generally, there should be no more than 3 such required experiments or major modifications for a "Major Revision" recommendation. If more than 3 experiments are necessary to validate the study conclusions, then you are encouraged to recommend "Reject".

Reviewer #1: 1. Establishing linkage between SNVs present in different parts of the virus genome is a non-trivial task using short read data, especially for viruses, for which there can be many distinct variants co-circulating. Yet, establishing these linkages is critical for many of the analyses presented in this paper and therefore, the authors need to convince readers (and reviewers) that the linkage they describe is accurate.

In general, I found the processes used for haplotype phasing (or at least their descriptions within the “Results” section) to be insufficient. For example, on lines 151-153 CG1 and CG2 seem to have been constructed by simply grouping together all SNVs with similar individual frequency estimates in these two samples, with no validation using within read linkage (as is used to some extent later in the paper). Also, on line 213-214, it is unclear how these composite sequences were generated and on lines 224-225, the reasons different SNVs were assigned to G1 vs. G2 is not clear.

We appreciate the reviewer's observations and the opportunity to clarify our methodology further. We understand that the distinction between the two sets of haplotypes—CG1/CG2 and G1/G2—was not adequately articulated. We updated the Results to reflect the following:

1. Initial Observations: We initially identified two clusters of mutations, termed CG1 and CG2, in two specific tissue samples, SSPE1 and SSPE2, during the pilot analysis. These mutations exhibited similar frequencies within these samples. Based on this preliminary data, we proposed the existence of two potential, ‘candidate’ viral haplotypes.
2. Expanding the Sample Size: Later, we had the opportunity to analyze an additional 13 tissue samples from the brain. Using variants identified across all 15 samples, we applied clustering based on their frequency distribution in each tissue (described in detail in the Methods). The logic behind this clustering was that if variants demonstrated consistent co-variation in frequency across tissues, they are likely on the same underlying molecule, or haplotype.
3. Additional Long Read Analyses: In a new experimental approach to validate linkage we performed nanopore long read sequencing of two samples that had acceptable RNA quality (thawing the brain did negatively impact RNA quality). The data in the new **Extended Data Figure 7** confirm the attribution of SNVs to either one or the other dominant haplotype.
4. Confirmation and Distinction: From the extended analysis, we derived the true identity of the two dominant haplotypes, named G1 and G2. These essentially validated our earlier hypothesis about the presence of CG1 and CG2 as two prominent haplotypes in the brain. The distinction between CG1/CG2 and G1/G2 rests in the methods that were used to define them, and in the specific mutations exclusive to the SSPE1 and SSPE2 samples. These CG1/CG2 specific mutations occurred against the background of the dominant haplotypes G1 or G2, and are consistent with the sub-haplotypes identified later in the manuscript.

The ”Methods” section includes a more detailed description of the later process for defining clusters, but this needs to be better reflected in the “Results” and the data used to try to deconvolute these clusters needs to be shown; for example, the correlations in the frequencies between variants in different samples and the actual frequencies of variants assigned to each cluster.

To address this point, we’ve **added a figure (Extended Data Fig. 11)** that shows the correlation between the frequency of SNVs in each tissue sample for each haplotype cluster. The plot is faceted by the cluster identity and each black line represents the frequency of a single SNV in that cluster. The colored ribbon represents the

mean frequency +/- the standard deviation around the mean frequency of each cluster. We've also **added a data table (Extended data Table 2**) that contains the frequency of the SNVs that make up each cluster in each tissue sample.

Also, physical linkage within individual sequencing reads should be used more broadly to test the linkage predictions made by the frequency correlations.

While we agree that using reads to provide evidence of physical linkage for SNVs in each haplotype would improve our analysis, the distance between SNVs often exceeds the maximum read length of our sequencing approach. However, we have made an effort to address this point as best as possible by including the following analyses:

Evidence for G1 and G2 using overlapping short reads:

We used a statistical framework adapted from the haplotyping approach CliqueSNV (Knyazev et al.,Nucleic Acids Res. 49, e102, 2021) to validate our frequency-based haplotyping approach for G1 and G2. CliqueSNV identifies haplotypes by checking the linkage of SNV pairs, creating a relational graph, merging graph cliques, and then partitioning reads to form haplotypes. However, due to the large degree of homoplasy complicating the graph structure, CliqueSNV isn't ideal for our dataset. Also, unlike our method, it doesn't use information from multiple specimens. We were, however, able to leverage CliqueSNV's statistical framework to assess whether G1 and G2 SNVs are "linked" or "forbidden" in the reads that overlap multiple SNVs.

To determine whether two SNVs, *A* and *B*, are "linked", CliqueSNV estimates the probability that there are no haplotypes simultaneously containing both *A* and *B*. If this probability is low, *A* and *B* are classified as "linked". Essentially, CliqueSNV is determining whether the observations of the *AB* haplotype (reads with both *A* and *B*) are more than would be expected due to chance.

On the other hand, to determine whether two SNVs, *A* and *B*, are mutually exclusive, or "forbidden", CliqueSNV checks that the probability of observing reads containing *A* and *B* is sufficiently low given that the true frequency of the haplotype is greater than what's expected by chance.

We used these two statistical tests (described in more detail in the methods) to calculate the proportion of SNVs representing the following *AB* haplotypes:

(1) *A* = G1 and *B* = G1
(2) *A* = G2 and *B* = G2
(3) *A* = G2 and *B* = G1 or *A* = G1 and *B* = G2

We chose 0.05 as the frequency that we expect a given *AB* haplotype to occur by chance. We believe this value is a reasonable choice given that the high rate of homoplasy throughout the genome could result in a low frequency presence of most possible *AB* haplotypes, particularly in the mutation-dense Matrix protein.

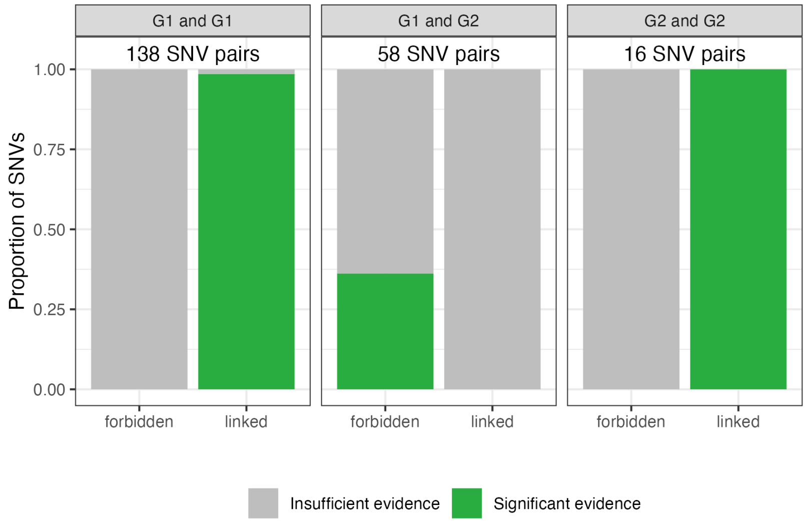

We used bridging reads over SNVs in each *AB* haplotype described above to calculate whether that pair of SNVs was linked, forbidden, or whether there wasn't sufficient evidence to determine the relationship. It's important to note that the absence of evidence for linkage does not imply that a pair of SNVs is forbidden and the absence of evidence that two SNVs are forbidden does not imply that they're linked.

As illustrated in the Figure adobe (same as the new **Extended Figure 6**) observed that nearly **all** SNV pairs were linked in cases where the SNVs were both G1 or both G2. Additionally, there were **no** forbidden pairs where both SNVs were G1 or both SNVs were G2. We also observed evidence that many SNVs pairs with one G1 and one G2 SNV were forbidden, and none of them showed a signature of linkage. Note - the finding that some G1/G2 SNV pairs show insufficient evidence for being "forbidden" does not mean that they're linked (indeed, we tested that they showed no evidence of linkage), merely that the read depth and allele frequencies do not permit us to confidently assess that they do not belong to the same haplotypes. We believe that this analysis provides convincing evidence that the G1 and G2 haplotypes exist and are mutually exclusive. We've included this analysis in the results and described how we did the analysis in the methods.

Evidence for G1 and G2 using overlapping long reads:

We performed Oxford Nanopore sequencing on a subset of samples to provide additional support for G1 and G2 using physical linkage over longer distances than possible with short read sequencing.

The new **Extended Data Figure 7** shows that even over longer distances, the SNVs within either G1 or G2 are present on the same molecules, and SNVs from G1 and G2 are mutually exclusive.

In summary, we have performed additional analyses that proved the existence of the dominant haplotypes, validated our frequency-based clustering methodology, and provided additional evidence for the existence of the sub-haplotypes.

On a related topic, it is unclear to me how and why SPRUCE/MACHINA was used for the generation of the phylogeny in Figure 7a. Line 570: "find all phylogenetic trees consistent with the average haplotype frequencies across the specimens." What exactly does this mean? What assumptions are being made regarding the relationship between haplotype frequency and phylogeny, and why is this needed, as opposed to simply using the inferred haplotype sequences alone? Has this approach ever been validated for use with viruses?

The challenge with directly using standard phylogenetic methods to build a tree based on haplotypes is that we do not have full haplotype information until we have inferred the phylogeny. We must therefore jointly infer the

phylogeny and the haplotypes, which is exactly what SPRUCE does. SPRUCE makes three assumptions in order to infer the phylogenies based on haplotype frequencies:

1) If a partial haplotype A descends from a different partial haplotype B, the frequency of A cannot by greater than the frequency of B in any compartment
2) The frequency of all haplotypes may not add up to a number greater than 1 in any compartment
3) The phylogeny is shared across all compartments.

Note, we have now explicitly added these assumptions to the last section of the Materials & Methods section where we introduce SPRUCE.

To give a concrete example of how this works in practice, we may observe a partial haplotype based on mutational correlations and observe that it contains mutations 1, 2 and 3. To construct a complete haplotype, we must determine whether it falls on the background of G1 (containing mutations 4, 5), G2 (containing mutations 6, 7, 8, 9), or neither. Therefore, the haplotype might be 1, 2, 3, 4, 5 OR it might be 1, 2, 3, 6, 7, 8, 9 OR it might simply be 1, 2, 3, but until we have assigned the partial haplotype to a background, we do not know which it is. Adjudicating between these options is what we mean by inferring the phylogeny, because determining which of these haplotypes could exist implies three different options for phylogenies:

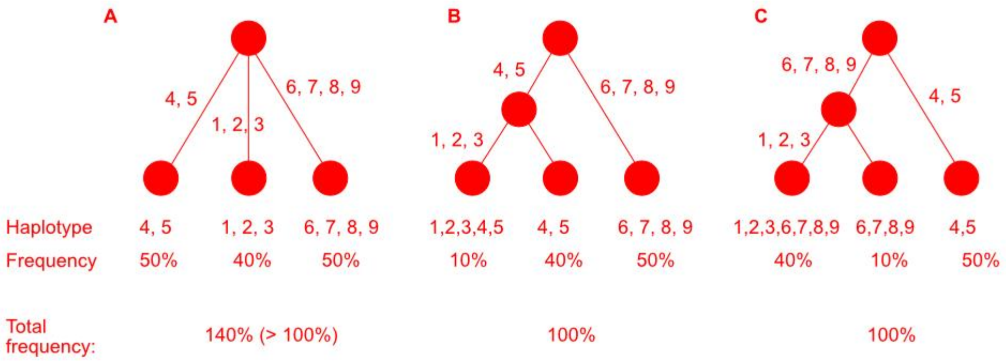

SPRUCE evaluates all three potential possibilities with the essential constraint that all haplotypes must not sum to a frequency greater than 1 in any compartment. [Note - we do not show even further options, like (1, 2, 3) descending from (6, 7, 8, 9) descending from (4, 5), but SPRUCE considers *all* tree topologies]. To make this more concrete, if, in a given compartment, we observe (1, 2, 3) at frequency 40%, (4, 5) at frequency 50% and (6, 7, 8, 9) at frequency 50%, we can deduce that (1, 2, 3) must fall on the background of either (4, 5) or (6, 7, 8, 9), because if it doesn't, the total frequency of haplotypes in each compartment will be greater than 100% (40% + 50% + 50%). Thus, we would write that this phylogenetic tree is *not consistent* with the haplotype frequencies across compartments, and we would not consider it moving forward (R1.A). However, it could be possible that haplotype (1, 2, 3, 4, 5) is at 40%, haplotype (4, 5) without mutations 1, 2, 3 is at frequency 10% and haplotype (6, 7, 8, 9) is at frequency 50% (Figure R1.B). This would explain all of the allele frequencies that we've observed AND it implies a specific set of phylogenetic relationships. On the other hand, if (1, 2, 3) falls on (6, 7, 8, 9), this also is a possible explanation of the allele frequencies but implies a different phylogeny (Figure R1.C). How can we determine which is the true underlying relationship?

In the measles dataset, we gain considerable power to further winnow the field of potential explanations for the allele frequencies because we have measured allele frequencies *across multiple compartments* and we assume that the phylogenetic tree is shared in each of those compartments (assumption 3). Therefore, if we observe in a separate compartment that (1, 2, 3) is at frequency 20%, (4, 5) is at frequency 90% and (6, 7, 8, 9) is at frequency 10%, this eliminates the potential explanation shown in Fig C, because of assumption 1: the frequency of (6, 7, 8, 9) is less than the frequency of (1, 2, 3) in this compartment, and thus this is not a valid haplotype arrangement.

Note, while it is possible to look at all of the trees and the frequencies in this example, in the measles dataset, we are balancing many, many more partial haplotypes, and enumerating all of the potential arrangements among these can no longer be done by hand. SPRUCE takes these collections of mutational frequencies across these different compartments and returns all possible haplotypes and trees that can generate these frequencies across all compartments. When we ran SPRUCE, we found that among the very large number of potential trees, 36 of them could plausibly explain all of the data. These 36 trees were mostly extremely similar, but differed in their placement of some of the clusters that were at relatively low frequency across all compartments.

To adjudicate between the remaining trees, we performed the linkage based analysis described in the Materials & Methods under phylogenetic reconstruction. In doing so, we were able to assign each subcluster to a specific background - either G1 or G2. In addition to weighing the evidence between the trees, this linkage based analysis acted as an internal validation that our inferred tree was self-consistent with all of the short-read linkage data. When we did this, 34 of the trees were eliminated (because they, for example, had hypothesized that a cluster belonged to G1 when read evidence suggested it belonged to G2). This left two remaining trees, one in which clusters 2 and 6 were linked, and one in which clusters 2 and 6 descended directly from G1 separately. Bridging reads again permitted us to eliminate one of these hypotheses - of nearly 5500 reads that spanned at least one cluster 2 SNV locus and one cluster 6 SNV locus across three spatial locations, 1928 reads contained cluster 2 SNVs, 435 contained cluster 6 SNVs, but 0 reads contained SNVs from both 2 and 6.

While to our knowledge, this approach has not been used in viral data, it is one of the most frequent analyses of cancer genomic data. It is called clonal deconvolution, and there are dozens of methods for performing it (most of the variation among these methods grapples with the cancer specific-issue of copy number alterations, but the core principles are always the same). We believe that SPRUCE is appropriate in this instance because none of its assumptions are cancer-specific, but rely on fundamental mathematical constraints on mutational frequencies in different populations.

However, the reviewer is certainly correct that this is an uncommon approach in the analysis of viral data with which most of our readers will be unfamiliar. **We therefore have expanded our description of the approach in both the Results and the last section of the Materials & Methods** (and the challenge with directly inferring the haplotypes and building a tree using more standard phylogenetic approaches).

2. It is unclear whether the SNV frequencies being reported were calculated using both genomic (- sense) and mRNA + antigenomic (+ sense) reads. This should be clarified and the authors should include an analysis to test whether frequency estimates of SNVs are consistent between - sense and + sense reads.

We appreciate the reviewer's insightful comment. We computed the reported SNV frequencies by considering both genomic (- sense) and mRNA (+ sense) reads separately. To determine if the origin of the reads (genomic or anti-genomic) influenced the SNV frequency, we categorized the aligned reads based on their + sense or - sense orientation before identifying SNVs. Subsequently, we visualized this by plotting the SNV frequencies from genomic reads on the y-axis against those from anti-genomic reads on the x-axis.

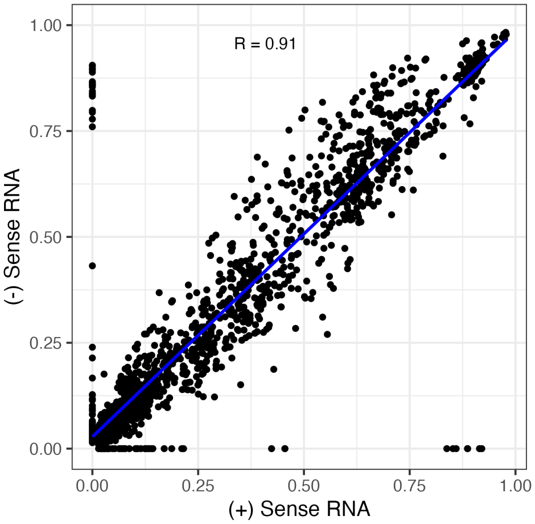

While some SNVs were exclusive to either genomic or anti-genomic reads (seen along the plot's periphery), there was a strong correlation in frequencies across the two types of reads, indicated by a Pearson's correlation coefficient of 0.91. We also investigated if the variances in SNV frequencies between the genomic and anti-genomic reads might carry biological significance. It's possible that the observed differences arise from sampling discrepancies rather than genuine biological causes. Upon filtering out SNVs from lower depth sites and increasing the depth threshold, we observed that the outlier SNVs disappeared and the correlation grew stronger, reaching a Pearson's correlation coefficient of 0.98.

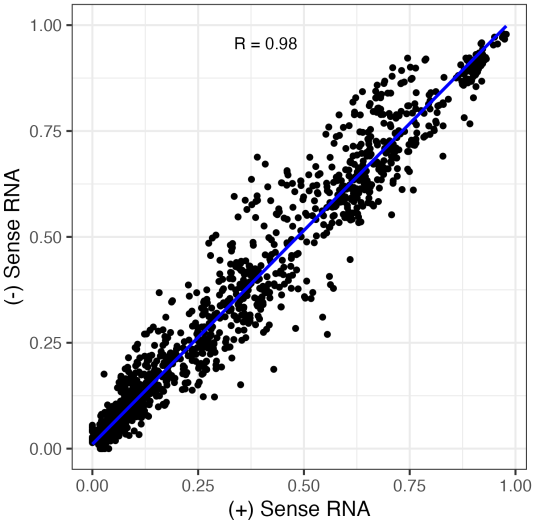

To enhance clarity, we've updated the manuscript to specify that the SNV frequencies were derived from both genomic and anti-genomic RNA. However, segregating these had a minimal effect on the SNV frequency outcomes.

3. There are many areas in which the authors overstate what they can infer from the data being presented. The phrasing needs to be modified throughout to better reflect what can be directly inferred from the data and the uncertainty associated with different interpretations.

a. The authors are clearly interested in the temporal dynamics of chronic measles virus infections, and they attempt to infer these dynamics through phylogenetic analyses. However, the fact remains that they only have samples from a single point in time. Therefore, in general, I found the conclusions regarding the temporal

dynamics of the infection to be overstated. Speculation about the temporal dynamics should be saved for the “Discussion” and presented with an appropriate level of uncertainty.

We have revised Abstract, Author Summary, and the last three sections of the Results to remove statements regarding temporal dynamics. In the Discussion, our interpretations are addressed with more uncertainty, and two paragraphs addressing limitations of the work were added.

b. It is also critical to keep in mind that this paper reports a single case study and therefore the results cannot be generalized to all SSPE cases. Therefore, the authors need to modify language in several places to reflect this. For example:

Line 398: “MeV replication is ubiquitous” to “can be ubiquitous”
Lines 398-399:: “is driven by multiple” to “can be driven by multiple”
Line 408: “the genome collective is an important” to “can be an important”

The text was changed as suggested.

c. Similarly, this paper does not include any functional studies to confirm the roles of specific mutations (or genome collectives) in allowing for spread within the brain. Therefore, conclusions regarding these aspects need to similarly be tempered.

We have tempered conclusions and have indicated which systems we have selected to perform functional studies to assess the role of specific mutations in infection spread.

Reviewer #2: The study's findings could benefit from greater clarity and elaboration. The report's strength is notably undermined by the use of non-specific language. Enhancing the report's robustness and clarity can be achieved by incorporating more details on the experimental design.

The study involved the extraction of two tissue samples from unidentified brain regions of a deceased individual aged 24. However, there is a significant lack of information regarding the donor, particularly in terms of relevant background details. It would be advantageous to provide specific information about the timing and location of the individual's infection, if available.

We agree that the information provided about this patient was minimal. This is due to ethical considerations. With rare diseases like SSPE, the CDC is hesitant to provide too much information because cases can be identified from other sources. We have re-structured the first two sections of the methods to include a statement indicating that disclosure of additional patient information is not possible.

To assess RNA quality, the researchers detected 18s and 28s. It would be beneficial to include the results obtained from running samples on an Agilent Bioanalyzer, if possible. This additional information would offer a more comprehensive understanding of the RNA's integrity.

To assess RNA quality (Figure 1a-b) we used methylene blue, which stains all types of RNA (not only 18S and 28S ribosomal RNA). In addition, we used Northern blots with specific probes, which can reveal broad smears due to degradation. We believe these techniques are at least as informative about the quality of the RNA than Agilent data. Nevertheless, we’ve included additional data below from Agilent fragment analyser to show that these results are consistent with Figure 1 a and b. This analysis was done at Mayo sequencing core to test the quality of our samples before sequencing. All samples had passed the standard quality control.

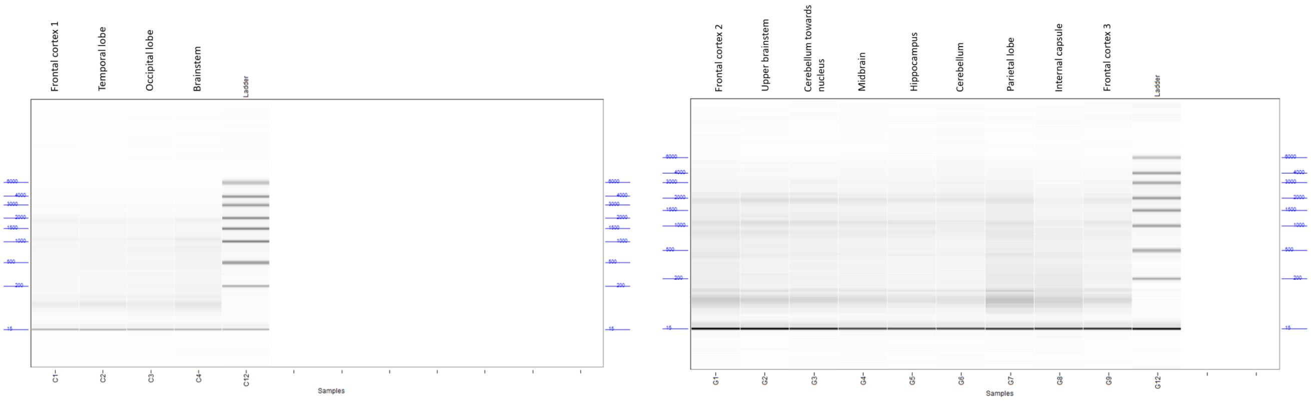

They then sequenced the RNA after depletion of rRNA and assessed the distribution of reads finding a huge reduction of reads towards the 5' end. My concern with this is that any conclusion that can be made from the RNA sequencing can only confidently be made for the 3' end as there are tens of thousands more data points than the 5' end. I would argue that the number of reads on the 5' are insufficient. The authors go on to make claims about RNA encoding envelope protein F and hemagglutinin which are in this low coverage region, which to me is not convincing. However, this problem doesn't seem to be the issue for the samples used later in the study.

Progressive reduction of mRNA levels towards the 5' end reflects the expected transcription gradient. To illustrate this gradient, in Figure 1c we used a linear scale for the vertical axis. As a consequence of this choice, reads per nucleotide for the F, H and L genes could be perceived as "insufficient". However, most reads are in the hundreds. The extent of coverage over the genome is better appreciated In Figure 4, where we used a logarithmic scale for the vertical axis. In most of the 13 samples examined, coverage exceeded 1000 over almost the entire genome length. We consider this coverage sufficient, and agree with the reviewer that this is not an issue for most data. Additionally, conclusions about the Fusion and Hemagglutinin protein are derived from the samples used later in the study, which the reviewer doesn't perceive a problem with.

In line 103 the authors mention an average coverage of .89 million reads/base but it is unclear if this is for what dataset.

We clarified in line 103 (Introduction) that "the combined deep sequencing data from all brain specimens covers the 15,894 bases MeV genome 0.89 million times". The Extended Data Table 1 (available in both original and revised manuscript) lists the MeV reads numbers (in millions) for the 15 specimens examined.

The authors compare the sequenced RNA to a composite sequence consisting of 13 isolates of MeV genotype D, which was the only known genotype circulating in Central America at the time of infection. If the date of infection is known, it would be helpful to include that information.

The date of infection is not known. However, we did infer that it occurred in Central America in the late 1980s, well before the Pan American Health Organization (PAHO) laboratory network was formed and started archiving and sequencing samples. As to the reference sequence, we regretfully misstated its identity, as explained in the answer to minor point 15 of reviewer 1.

Additionally, reporting an estimation of viral RNA in each sample is crucial to determine whether the sequencing analyses could have been affected by the jackpot effect.

We agree that indicating the number of viral RNA reads in each sample is crucial. We wonder whether the reviewer overlooked Extended Data Table 1 that lists the MeV reads numbers (in millions) from the 15 specimens examined.

Regarding lines 230 to 240, while the use of a composite (PCA) makes sense to some extent, it is necessary to compare the sequenced RNA to each individual sequence genome used in the PCA. Conducting pairwise comparisons would provide insights into the patient's relationship with each genome, highlighting the most and least related ones. Supplementary material could be utilized for presenting this information.

In this analysis, we sought to determine if the genetic composition of samples nearby in space was similar. A standard approach in the field would be to use PCA, as it flattens a high dimensional dataset into a lower dimensional representation. In this case, the high number of dimensions comes from the frequency of each sampled allele from each tissue. Therefore, the PCA is not performed on individual sequenced genomes, but rather on a matrix in which every row represents a spatial location and each column represents the frequency of an allele in that location. These approaches have been used fruitfully to show that genetic data sometimes permits a remarkably accurate reconstruction of spatial layout (November et al, Nature 456, 98-101, 2008).

In this way, we do not fully understand the suggestion made by the reviewer, but, as we understand it, the reviewer is asking for these comparisons in distinct spatial locations to be broken down by individual genome sequences presented in each of those regions. This is only possible to do after we have inferred which individual genome sequences are present in each location and at what proportion. This information is plotted in **Fig 7b**.

Because we did not properly communicate the point of this analysis, we have included more description of what we did and why in the first paragraph of the last section of the Results (Spatial dynamics of the collective infectious unit).

Furthermore, for Figure 2, it is important to include the SSPE2 data alongside the shown SSPE1 data, especially considering its discussion in line 146. It would also be beneficial to provide the raw counts used in Figure 2, in addition to the frequency percentages.

We have added the SSPE2 data to Figure 2 as the reviewer suggests. To address the second point, we have updated Figure 1 to include viral reads percentages from both samples.

When discussing the Candidate Brain Ancestor and Candidates Genotypes 1 and 2, it is crucial to include metadata on the circulating strains at the infection location.

What we know is that this infection occurred in the late 1980s, well before the Pan American Health Organization laboratory network was formed and began collecting metadata.

For in situ analysis, it would be beneficial to have the breakdown of CG2 cells in different brain regions, preferably presented in a table format like Table 1.

The breakdown of CG2 cells is presented in Extended Data Figure 3 (ED figure 2 in the original version). We have added a footnote to Table 1 to indicate where this information is found.

I have reservations about the conclusion that MeV spreads from the frontal cortex to other regions without sufficient evidence. The discussion on viral spread within the brain is lacking. It is possible that MeV starts in the cerebellum and spreads from there. This speculative theory could explain why there are fewer reads from the cerebellum, indicating more cell death and suggesting an earlier infection timepoint. This timeline would be contrary to what is shown in Figure 8. It is important to explore alternative hypotheses or provide clarification to attenuate the conclusion.

We regret that the initially reviewed manuscript overstated the certainty of its conclusions regarding the probable site where the MeV spread started. The revised manuscript states that we cannot definitively chart the history of spread, as the reviewer correctly notes. However, we have a number of observations that are challenging to explain under alternative models. The revised Discussion considers alternative hypotheses, and

we explain why we do not think the data is as consistent with these hypotheses. We address the suggestion that low viral RNA counts in the cerebellum could be due to more cell death In the second paragraph of the Discussion.

Additionally, in Figure 6, they demonstrate the reads from 13 different brain regions, raising the question of why they used SSPE1 reads for comparison with the compound genome.

We organized the paper keeping in mind the order of our findings in an effort to help guide the reader. In the initial pilot experiments with SSPE1 and SSPE2, we didn't include the patient-specific consensus sequence because we couldn't have made this sequence without access to the other 13 tissue samples. The conclusions that are derived from the initial pilot analysis with SSPE1 and SSPE2 are not dependent on the reference sequence used to identify SNVs.

When an additional 13 tissue samples became available, we used these samples (in addition to SSPE1 and SSPE2) to create the patient-specific reference. We did use this reference to identify SNVs in not only the 13 tissue samples collected later in the paper, but also SSPE1 and SSPE2, as is reflected in Figure 6.

We modified the title of Figure 6 and modified the corresponding section of the Results to enhance clarity.

Lastly, in Extended Figure 4, the numbers do not add up to 100%.

In Extended Figure 4, we plot the percentage of all reads that map to the MeV genome in each region, and therefore, they do not need to add up to 100%. We have edited the figure legend to make this point clear and explain our methods better.

Reviewer #3: (No Response)

Part III – Minor Issues: Editorial and Data Presentation Modifications
Please use this section for editorial suggestions as well as relatively minor modifications of existing data that would enhance clarity.

Reviewer #1: 1. It is unclear to me why the authors are not able to provide an approximate location in the brain from which the pilot samples (SSPE1 and SSPE2) were taken. If the brain was frozen intact, which seems to have been the case, then it should have been clear from which region the samples were collected. This needs to be addressed.

We have changed the text to indicate that the samples were collected from the surface of the frontal lobe from the frozen brain by using a scalpel and tissue punch.

2. I would recommend that the authors reconsider the naming scheme that they use throughout the paper and consider whether all of the abbreviations are needed. It is not very easy for a reader to keep track of the differences between CG1, CG2, G1, G2, G01, G01-a, G01-b, etc.

We agree that the nomenclature is complex. However, we have considered alternative options, but concluded that it is best to proceed with the current nomenclature.

3. Lines 210-211: "Robust MeV replication facilitated extensive data collection:" I would recommend cutting this statement. It is a somewhat strange, and perhaps misleading, way to introduce the fact that they generated a lot of MeV sequence reads. But an equal number of MeV reads could also be generated from lower titer samples, it would just require more total sequencing depth.

The sentence was deleted as suggested.

4. Similarly, “because we adopted a more powerful sequencing technology” (Line 217) is not needed. The key is that they generated more sequencing reads, not the technology.

The words were deleted as suggested.

5. Line 232, “similarly” should be “similarity”

This has been corrected.

6. Line 270: “unlinked to F-Q527*, F-E526*” – please clarify what this conclusion is based on.

Thank you for noticing this oversight. The following sentence has been added after line 302: “Among 11941 reads overlapping F-Q527* and F-E526* in the Internal Capsule and Brain Stem, 5634 reads contained just F-Q527*, 1361 contained just F-E526*, and 1 read contained both SNVs.”

7. In general, the “Results” is really a mixture of “Results” and “Discussion”, but lines 272-277 are particularly speculative and would be a better fit for the “Discussion.”

We have broadly reorganized the Results and Discussion for a better separation of displaying the data and interpreting it. In particular, now there is no mention of frequency-dependent selection in the Results.

8. Lines 290-291: If you are going to report an insignificant correlation (p=0.36), then you should show the correlation to allow the reader to judge the relevance of the trend.

The frequencies of the two mutations in the same spatial locations are shown in Extended data Figure 10 (previously ED Figure 7). We have added a pointer to the Results to make this easier for the reader to find.

9. Line 303: Clarify the meaning of “flat phylogeny.” Do you mean that it includes a large polytomy?

The reviewer is correct that this is what we intended. We have changed the language to be more direct: “reflecting an almost flat phylogeny” has been changed to “reflecting few shared SNVs beyond those on the G1 and G2 clusters”

10. Please clarify the relevance of “no reports of measles cases in the country of residence since 2009” (Line 427) given that the patient developed symptoms in 2008.

Sorry for the lack of clarity. This sentence addresses the fact that measles can cause acute encephalitis, albeit with a clinical presentation that is different from SSPE. Nevertheless, to rule out acute encephalitis in this patient it is important to note the lack of measles activity since 2009 in the country in which the SSPE case was detected. In retrospect, the statement “no reports of measles cases in the country of residence since 2009” is somewhat irrelevant because the sequence obtained from the SSPE case was closely matched to the D3 genotype sequences from the late 1980s and early 1990s and quite different from the sequences of the wild-type MeVs circulating in 2009. We have re-structured the two first sections of the Materials and Methods to clarify our thinking.

11. Line 430: “a standard assay” – please provide a brief description, not just a citation.

A brief description was added.

12. Line 449: “comprising MeV nucleotides 5-254” – provide a GenBank (or similar) accession # so that readers can unambiguously find the relevant sequence.

We have provided the GenBank accession number.

13. Lines 454 and 455: spell out smFISH and ampFISH when first used.

These abbreviations are defined in lines 179 and 182.

14. Illumina sequencing – please clarify the lengths of the reads being generated. Are they 2 x 150 nt? Please also indicate the average length of the sequenced fragment (which can be inferred from the mapping data). This is important for understanding the limitations of analyses examining the physical linkage of SNVs.

The reads were 2 x 150 nts, and the fragment length averaged across all 15 samples was 194 bases with a standard deviation of 87 bases. We've added this to the description of the Illumina sequencing, lines 562-563.

15. Line 486: Why 13 isolates? Is this everything that is available?

We regretfully misstated the identity of the reference sequence. We initially aligned the sequences from the SSPE1 and SSPE2 specimens to a consensus sequence of 13 isolates. However, after CDC informed us that the sequence obtained from this SSPE case was closely matched to genotype D3 sequences from the late 1980s and early 1990s, we created a reference genome based on the best characterized D3 strains known to have circulated then. We have updated the methods describing how this chimeric reference genome was made.

16. The methods described on Lines 491-501 appear to have only been used for generating read count tables, and then a different, but similar pipeline was used for the rest of the analyses. Can these read count tables not simply be re-generated using the alignment files from the Snakemake pipeline? This would simplify the methods section and help avoid later confusion.

The reviewer is correct that these could be combined into a single pipeline for simplicity. We've updated the pipeline accordingly.

17. "The raw BAM files from Illumina sequencing" – This statement is confusing. Raw data from an Illumina machine should be in fastq format. A bam file is only generated once the reads have been aligned against a reference. Please clarify what reference was used to generate this BAM and whether there was any filtering based on whether the reads did or did not align to that reference.

Thank you for pointing this out. The files initially came from the core aligned to the human genome. We then extracted all the reads from these BAM files to generate unaligned FASTQ files. This was the starting point for our analyses described in the paper. We've updated the methods to reflect this.

18. Please report the breadth and depth of the sequencing data for each sample.

The read depth by position is illustrated in Figure 4 for each sample. The total number of reads is indicated in Supplementary Table 1 for each sample.

19. Lines 535-536: "we did not benchmark our approach to detect these." Did you benchmark your approach to detect SNVs?

The language used here isn't quite accurate. While we didn't explicitly benchmark our approach for SNVs, our approach is consistent with approaches used to identify SNVs in other studies. The main reason for not including insertions or deletions was that SNVs were sufficient for our haplotype analysis. We've removed the highlighted text from the manuscript.

20. Line 541: "to resolve the SNVs" – how were they resolved?

We employed two distinct variant callers to detect mutations in the measles virus genome. In consolidating the data from both callers, our intention was to eliminate variants found by only one method to reduce potential false positives. However, we observed cases where a variant was detected by both methods in one tissue, but only by one method in another tissue. Given that these variants were recognized by both callers in certain tissues, they are likely genuine variants. Excluding them based solely on their absence in one method for a specific tissue could lead to false negatives. To address this, we retained all variants identified by both callers in any tissue, even if they were detected by only one method in another tissue. We have updated our documentation to reflect this methodology.

21. Lines 553-554: Why did you choose 3 as the number of haplotype clusters?

This number of clusters was initially determined by visual inspection. For the SNVs that were identified in every tissue sample, we calculated the mean correlation coefficient across all pairs of SNVs. We noticed that most SNVs were either strongly correlated or anti-correlated. However, there was a subset of SNVs that weren't strongly correlated with any other SNVs.

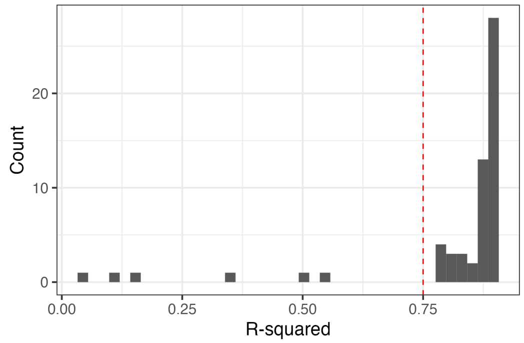

This observation is easily visualized by a histogram of the mean R-squared for each SNV. We took the most correlated SNVs (R-squared > 0.75) and clustered these using a k-medoids clustering approach.

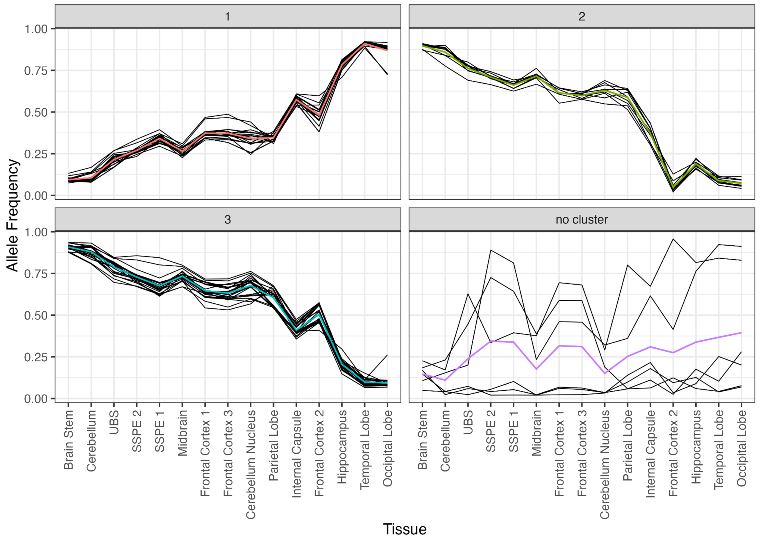

Although two clusters explained the data fairly well in most tissues, this wasn't the case for the Frontal Cortex 2 sample. In this sample, mutations in cluster 3 (what would eventually be called Genome 01) are nearly 50% frequency, while the mutations in cluster 2 (Genome 1) are nearly absent from the Frontal Cortex 2 sample. This led us to choose three clusters as the most parsimonious explanation of the data. This choice was later confirmed with additional evidence from read overlap. We've included language in the manuscript to explain our choice.

22. Line 555: “Following the success…” – what metric was used to establish success?

This language was insufficiently precise and we have edited the sentence as follows:
Old: “Following the success of exclusively using SNVs present in every specimen,”
New: “After identifying and clustering SNVs present in every specimen,”

23. Line 578: “Because G1 and G2 are mutually exclusive” – what is the evidence to support this? Did you look for G1 and G2 mutations within the same reads to show that this doesn’t occur?

We have now added statistical linkage (**Extended Data Fig. 6**) and long-read based analyses (**Extended Data Fig. 7**) to support that G1 and G2 are mutually exclusive.

24. Figure 1c – why are results only being shown for SSPE1? It would be better to switch to a stacked bar graph and show the SSPE1 and SSPE2 results side by side.

The figure has been updated according to the reviewer’s suggestion.

25. Figure 2 legend – “dots” should be “circles”

This has been updated in the text.

26. Figure 3 legend is too brief. Please provide more info about the methods and interpretation of this figure.

We’ve added more information to the legend.

27. Figure 4: “upper right” should be “lower right”

We’ve updated the text.

28. It would be useful to have a main figure that highlights the locations of the different regions of the brain, similar to ED Fig 4.

We’ve added this information to **Fig. 7b**.

29. Figure 6 – why is the term “allele” used instead of SNV?

Thank you for pointing this out. We’ve replaced the term allele with SNV in the text for consistency.

30. Figure 8 – The figure title needs to make it clear that this is a proposed model for what happened, but that the data presented cannot be used to unambiguously infer everything being shown in this figure.

We’ve changed the title to “Hypothetical reconstruction of the evolution of a MeV collective infectious unit in a human brain” to reflect the reviewer’s point. We’ve also supplemented the discussion of this figure to make it clear that it represents a hypothesis.

31. Figure 8 - Please do not include “For details see main text”. Figures (and legends) should be able to stand on their own.

We’ve removed this and extended the description present in the figure legend itself.

32. ED Figure 1a needs more explanation. It is not intuitive for someone without experience with ampFISH.

We’ve updated the description of this figure with more details; we also split the original Figure 1 in two to make space for additional control data requested in point 33.

33. Associated with ED Figure 1, are there controls that can be shown to demonstrate that this experiment worked as expected?

We have added a healthy human sample (ED Figure 2c) as a negative control to show that AmpFISH probes are specific to MeV RNA.

34. ED Figure 5 – "Frequency is relative to a patient-specific reference" – do you mean that mutations were called relative to the reference? Frequency should not depend on the reference. Also, has this reference sequence been deposited in a public repository?

The patient specific reference is available on GitHub along with the code for the study. We've added the link (https://github.com/jbloomlab/MeV_SSPE_Dynamics/blob/main/config/ref/MeVChiTok-SSPE.fa) to the "Data Availability" section of the manuscript.

You're correct. We mean that mutations were called relative to the "BA" patient-specific reference. We've updated the text accordingly.

35. ED Figure 7 – specify the meaning of the width of the red and blue ribbons

The width of the ribbon is the mean +/- the standard deviation around the mean allele frequency for G1 and G2 in each tissue. We've added a description of this to the figure legend (now ED Fig. 10).

Reviewer #2: (No Response)

Reviewer #3: I would like to ask the authors to please define 'collective infectious units' and 'RNA genome collective' as early as possible in the manuscript.

Thank you for pointing out this issue. In the original manuscript we were using "collective infectious units" and "RNA genome collectives" interchangeably. For clarity, in the revised manuscript we define "collective infectious units" and "genome collectives" in the first sentence of the Abstract, and use only the first term in the rest of the manuscript.

Would it be possible to determine the effects of amino acid changes observed here in F and M proteins on trans-synaptic spread in neuronal cell cultures? It would be of interest for the authors to mention this possibility in the discussion.

We have generated recombinant MeV with individual amino acid changes proposed to drive brain tropism acquisition, either by enhancing intercellular spread between neurons or intracellular transport of viral components. We are assessing the effects of these mutations in different experimental systems as specified in the updated Discussion (lines 444-448).
